# Supplementary material for: Disease-specific tau filaments assemble via polymorphic intermediates
Source: Nature. 2023 Nov 29;625(7993):119–25. doi: 10.1038/s41586-023-06788-w (PMC10764278; doi:10.1038/s41586-023-06788-w)
Supplement: Supplementary file 1 — This file contains Supplementary Figs. 1–53 and Tables 1–29. [file 41586_2023_6788_MOESM1_ESM.pdf]

nature portfolio

https://doi.org/10.1038/s41586-023-06788-w

Supplementary information

Disease-specific tau filaments assemble via polymorphic intermediates

In the format provided by the authors and unedited

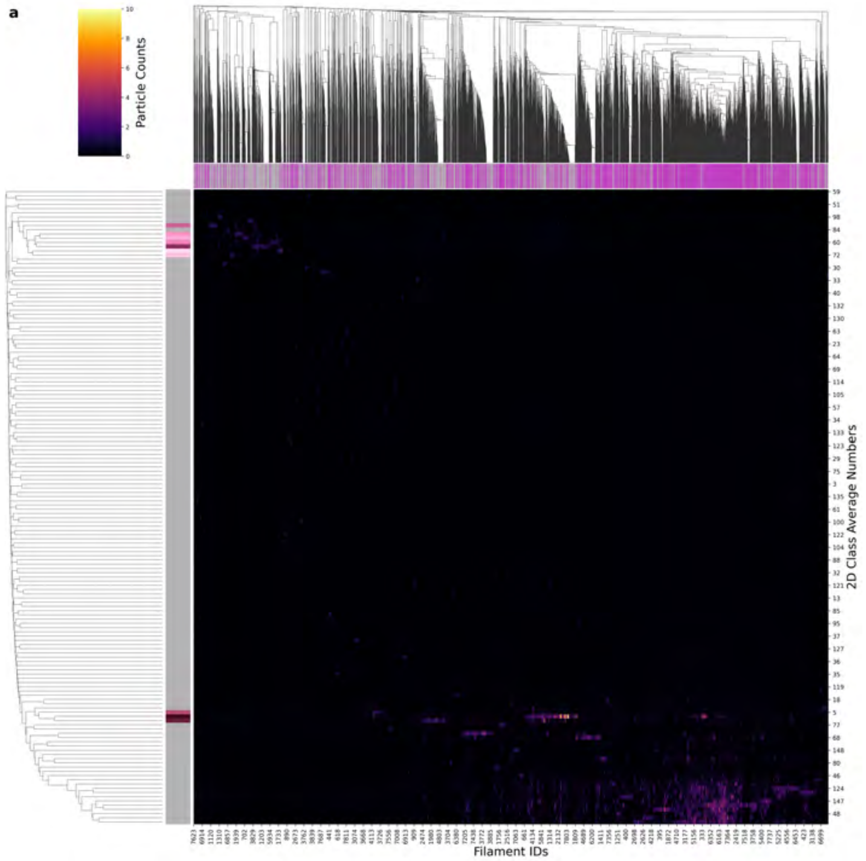

**Condition**	PHF
**Timepoint**	120 minutes
**Replicate**	1
**Picking**	Auto
**Micrographs**	9293
**Extracted Particles**	1714140
**Pixel Size**	0.727 Å
**Classified Particles**	60982

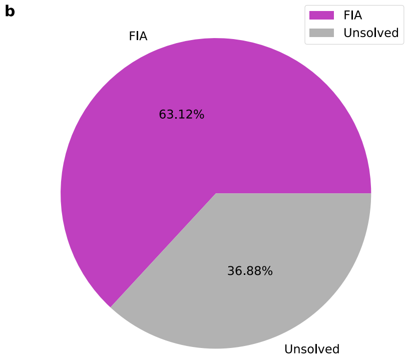

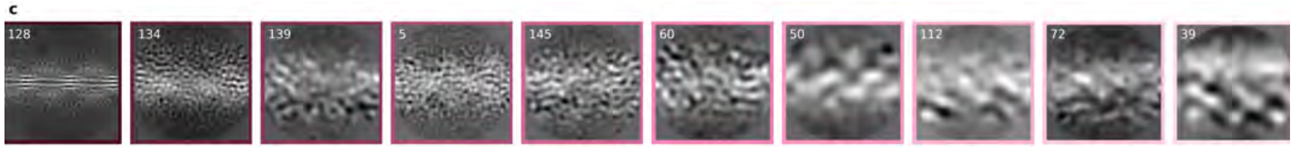

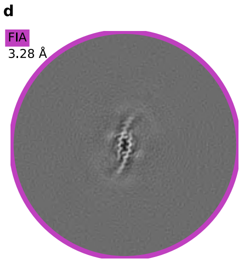

Supplementary Figure 1: Cryo-EM data set summary 1. Data set characteristics are specified in the table on the top right. **a** Hierarchical classification of individual filament segments according to their assigned 2D class average (vertical) and the picked filament ID(horizontal). **b** Pie chart with the relative amounts of different filament types. Grey represents unsolved filaments. Filament types are the same as in Figure 4 of the main text. Different colours represent different time points (120 min in purples; 180 min in blues; 240 min in greens; 300 min in yellows; 360 min in oranges and 720 min in reds). Structures are coloured according to the time point at which they are most abundant, averaged across all replicates. Unique names of filament types are indicated and the same names are used throughout this document. **c** 2D class averages of unsolved filaments. **d** XY-cross-sections, with a projected depth of approximately 4.7 Angstrom or each filament type.

**Condition**	PHF
**Timepoint**	120 minutes
**Replicate**	2
**Picking**	Auto
**Micrographs**	9340
**Extracted Particles**	1680613
**Pixel Size**	0.876 Å
**Classified Particles**	1680613

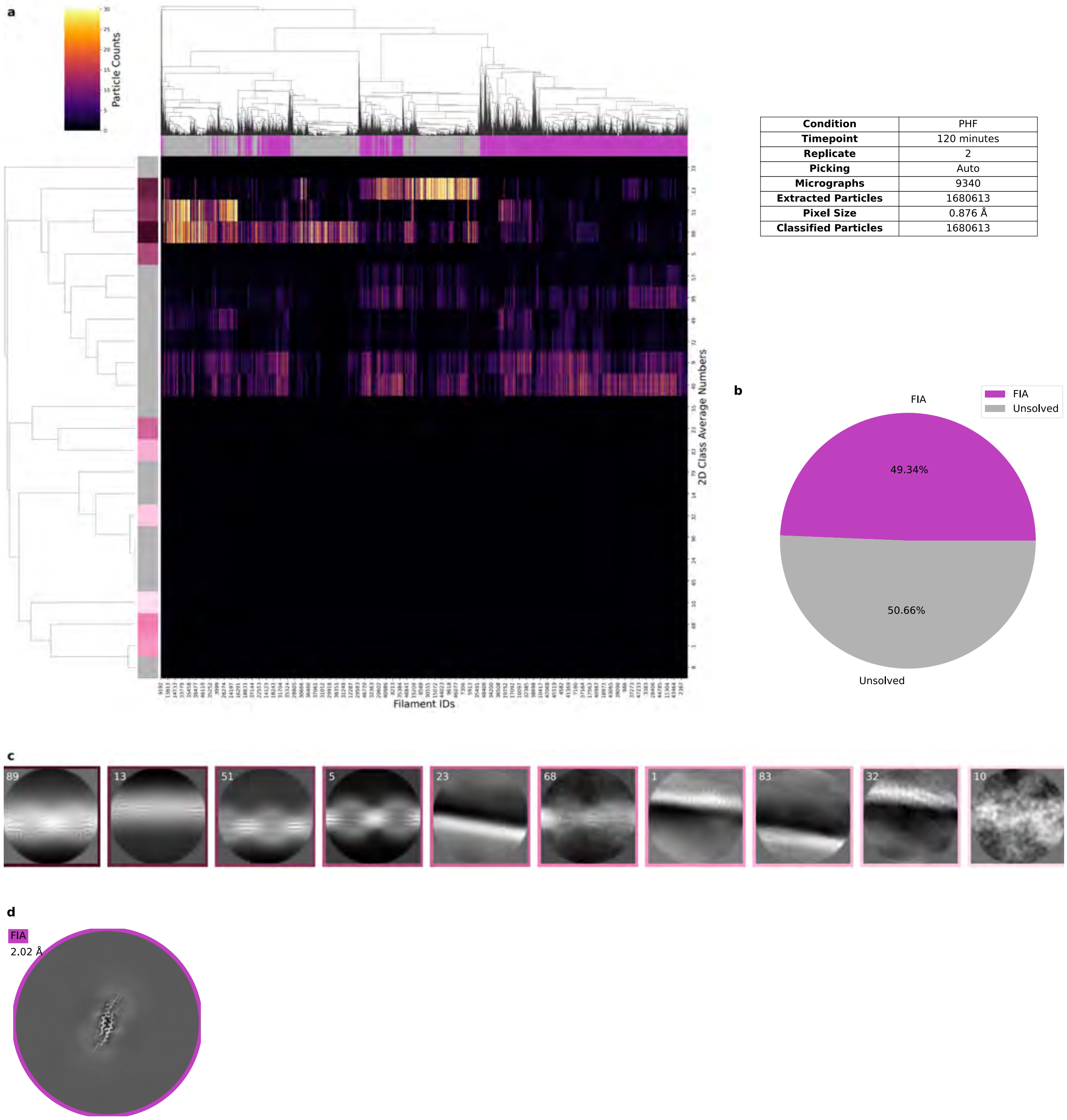

Supplementary Figure 2: Cryo-EM data set summary 2. Data set characteristics are specified in the table on the top right. **a** Hierarchical classification of individual filament segments according to their assigned 2D class average (vertical) and the picked filament ID(horizontal). **b** Pie chart with the relative amounts of different filament types. Grey represents unsolved filaments. Filament types are the same as in Figure 4 of the main text. Different colours represent different time points (120 min in purples; 180 min in blues; 240 min in greens; 300 min in yellows; 360 min in oranges and 720 min in reds). Structures are coloured according to the time point at which they are most abundant, averaged across all replicates. Unique names of filament types are indicated and the same names are used throughout this document. **c** 2D class averages of unsolved filaments. **d** XY-cross-sections, with a projected depth of approximately 4.7 Angstrom or each filament type.

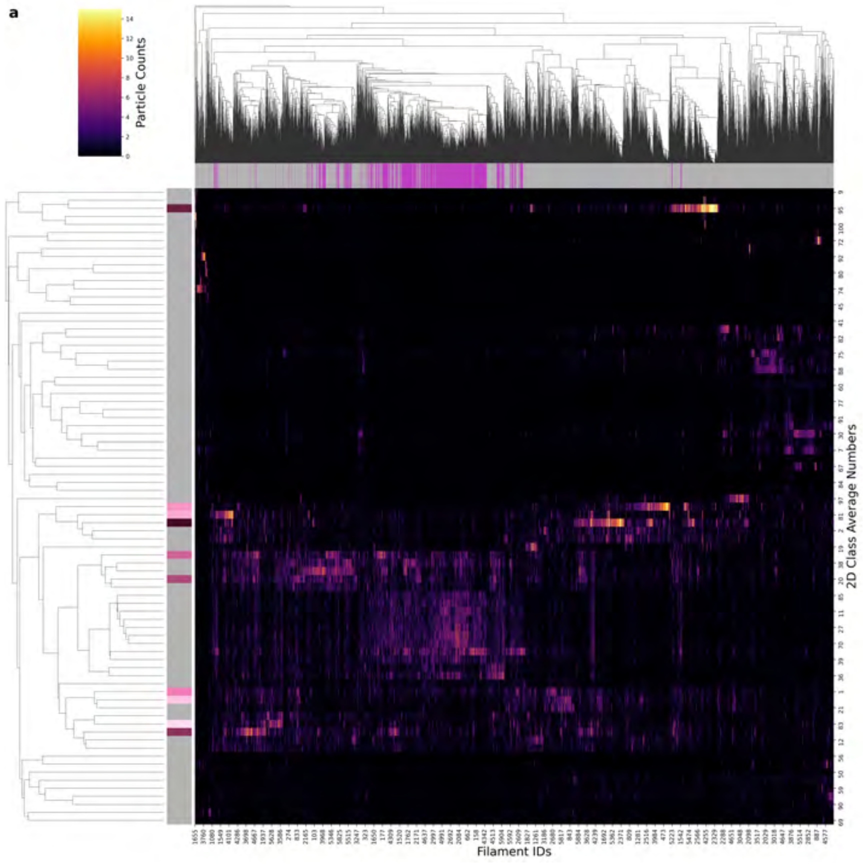

Condition	PHF
Timepoint	120 minutes
Replicate	4
Picking	Auto
Micrographs	1503
Extracted Particles	197780
Pixel Size	0.727 Å
Classified Particles	197780

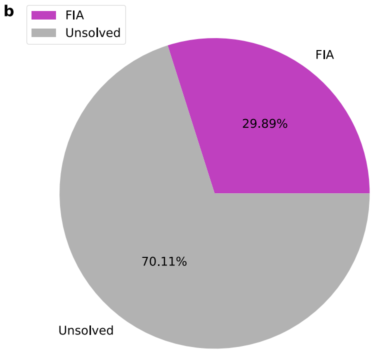

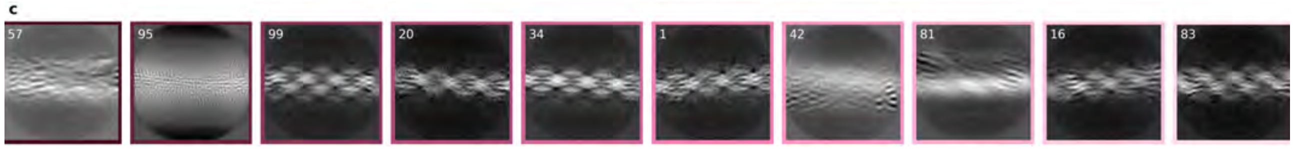

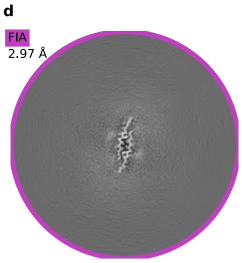

Supplementary Figure 3: Cryo-EM data set summary 3. Data set characteristics are specified in the table on the top right. **a** Hierarchical classification of individual filament segments according to their assigned 2D class average (vertical) and the picked filament ID(horizontal). **b** Pie chart with the relative amounts of different filament types. Grey represents unsolved filaments. Filament types are the same as in Figure 4 of the main text. Different colours represent different time points (120 min in purples; 180 min in blues; 240 min in greens; 300 min in yellows; 360 min in oranges and 720 min in reds). Structures are coloured according to the time point at which they are most abundant, averaged across all replicates. Unique names of filament types are indicated and the same names are used throughout this document. **c** 2D class averages of unsolved filaments. **d** XY-cross-sections, with a projected depth of approximately 4.7 Angstrom or each filament type.

Condition	PHF
Timepoint	180 minutes
Replicate	2
Picking	Manual
Micrographs	2430
Extracted Particles	122414
Pixel Size	0.727 Å
Classified Particles	122414

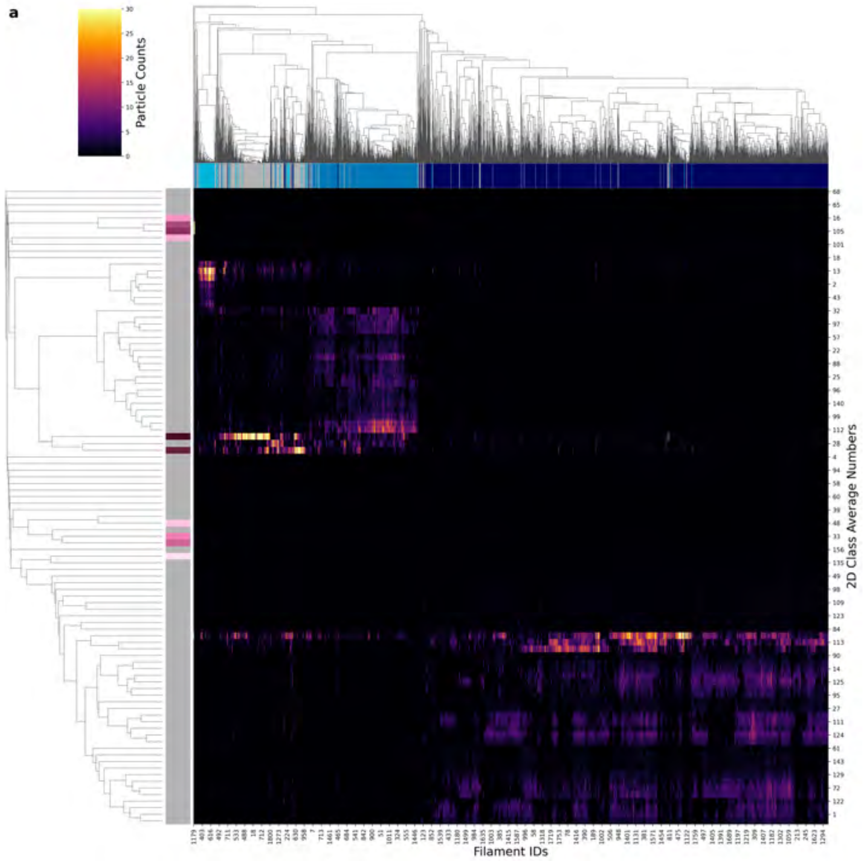

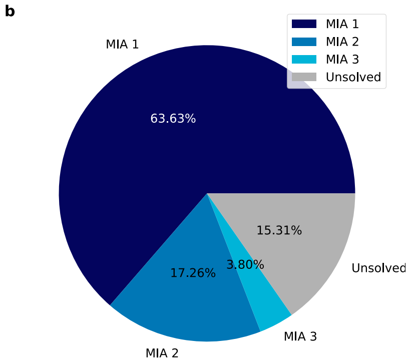

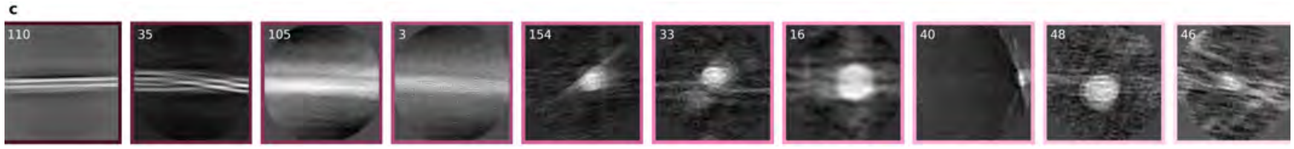

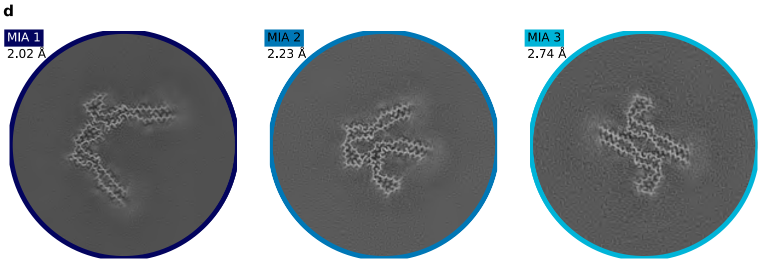

Supplementary Figure 4: Cryo-EM data set summary 4. Data set characteristics are specified in the table on the top right. **a** Hierarchical classification of individual filament segments according to their assigned 2D class average (vertical) and the picked filament ID(horizontal). **b** Pie chart with the relative amounts of different filament types. Grey represents unsolved filaments. Filament types are the same as in Figure 4 of the main text. Different colours represent different time points (120 min in purples; 180 min in blues; 240 min in greens; 300 min in yellows; 360 min in oranges and 720 min in reds). Structures are coloured according to the time point at which they are most abundant, averaged across all replicates. Unique names of filament types are indicated and the same names are used throughout this document. **c** 2D class averages of unsolved filaments. **d** XY-cross-sections, with a projected depth of approximately 4.7 Angstrom or each filament type.

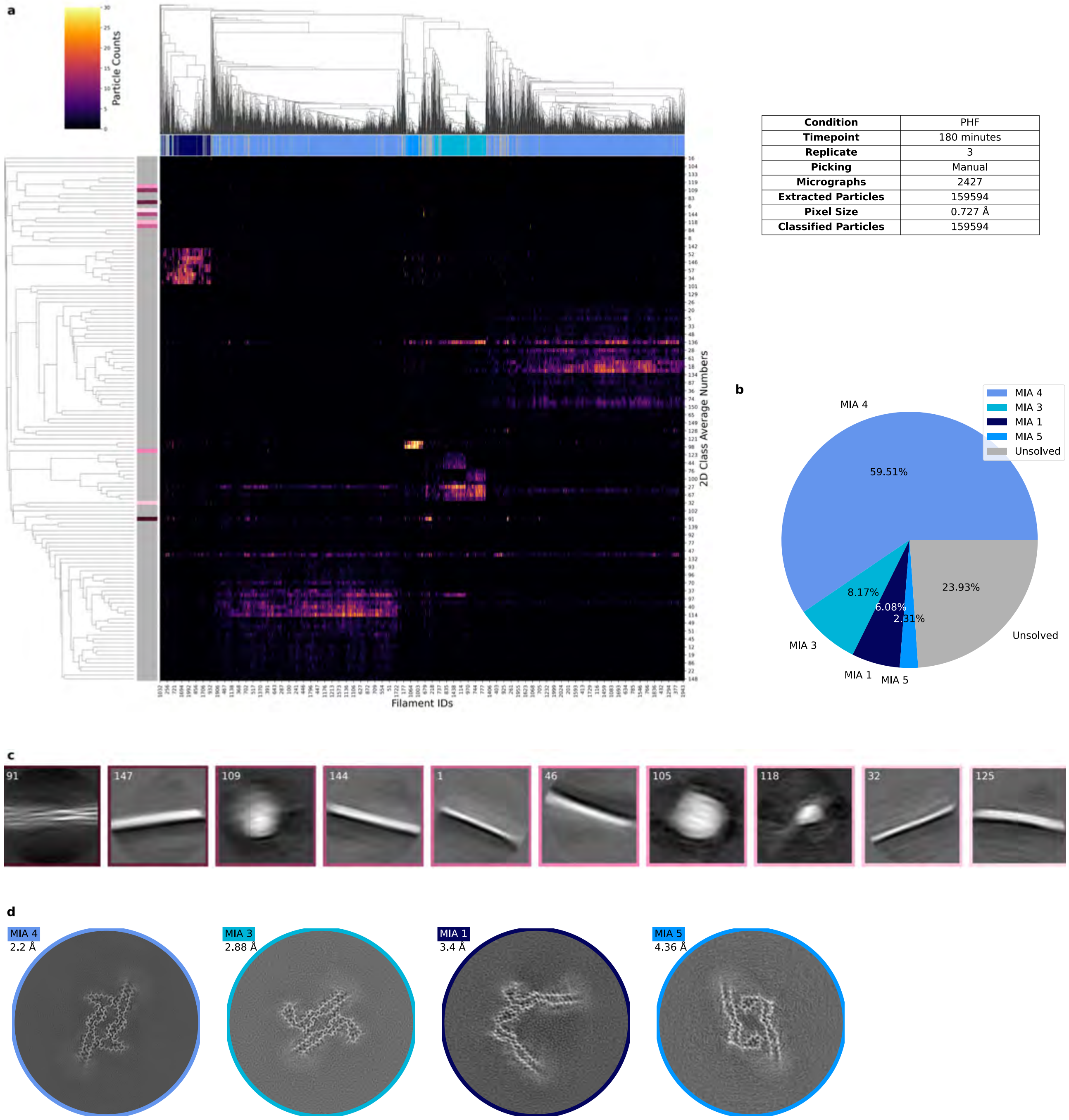

Condition	PHF
Timepoint	180 minutes
Replicate	3
Picking	Manual
Micrographs	2427
Extracted Particles	159594
Pixel Size	0.727 Å
Classified Particles	159594

Supplementary Figure 5: Cryo-EM data set summary 5. Data set characteristics are specified in the table on the top right. **a** Hierarchical classification of individual filament segments according to their assigned 2D class average (vertical) and the picked filament ID(horizontal). **b** Pie chart with the relative amounts of different filament types. Grey represents unsolved filaments. Filament types are the same as in Figure 4 of the main text. Different colours represent different time points (120 min in purples; 180 min in blues; 240 min in greens; 300 min in yellows; 360 min in oranges and 720 min in reds). Structures are coloured according to the time point at which they are most abundant, averaged across all replicates. Unique names of filament types are indicated and the same names are used throughout this document. **c** 2D class averages of unsolved filaments. **d** XY-cross-sections, with a projected depth of approximately 4.7 Angstrom or each filament type.

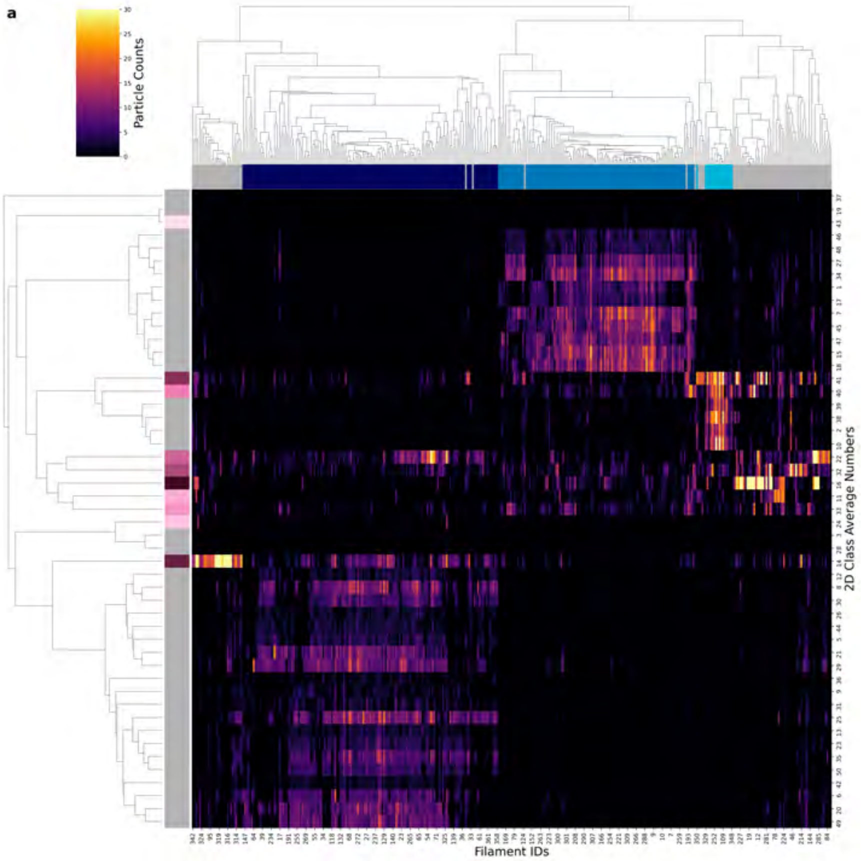

Condition	PHF
Timepoint	180 minutes
Replicate	4
Picking	Manual
Micrographs	596
Extracted Particles	30133
Pixel Size	0.727 Å
Classified Particles	30133

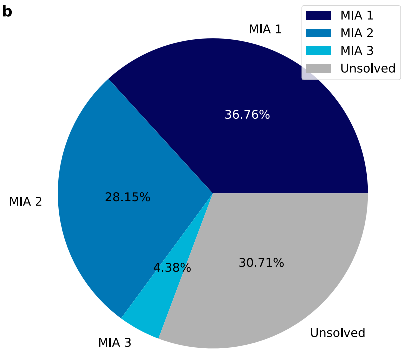

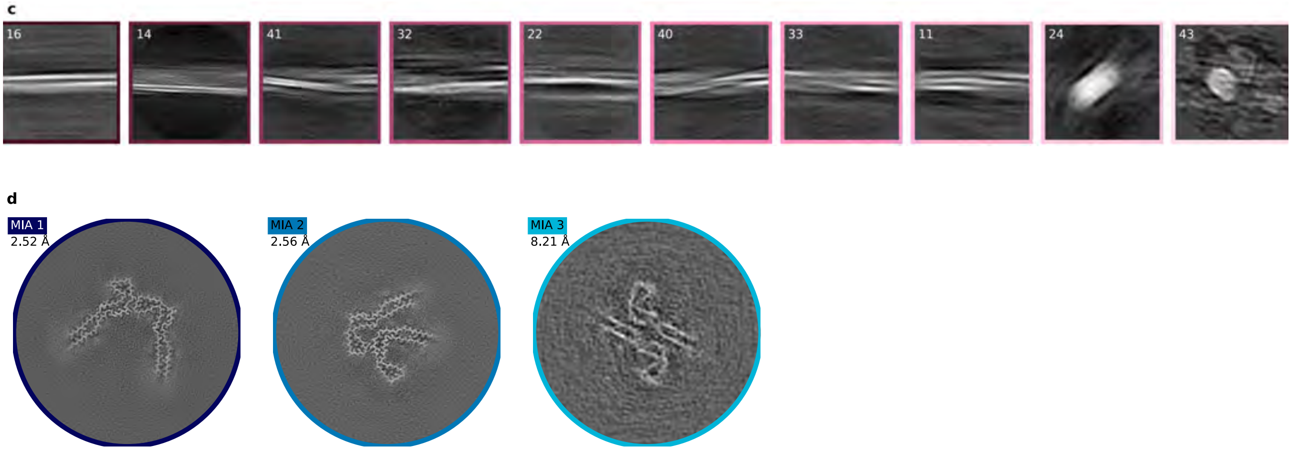

Supplementary Figure 6: Cryo-EM data set summary 6. Data set characteristics are specified in the table on the top right. **a** Hierarchical classification of individual filament segments according to their assigned 2D class average (vertical) and the picked filament ID(horizontal). **b** Pie chart with the relative amounts of different filament types. Grey represents unsolved filaments. Filament types are the same as in Figure 4 of the main text. Different colours represent different time points (120 min in purples; 180 min in blues; 240 min in greens; 300 min in yellows; 360 min in oranges and 720 min in reds). Structures are coloured according to the time point at which they are most abundant, averaged across all replicates. Unique names of filament types are indicated and the same names are used throughout this document. **c** 2D class averages of unsolved filaments. **d** XY-cross-sections, with a projected depth of approximately 4.7 Angstrom or each filament type.

Condition	PHF
Timepoint	240 minutes
Replicate	1
Picking	Manual
Micrographs	7262
Extracted Particles	352893
Pixel Size	0.824 Å
Classified Particles	352893

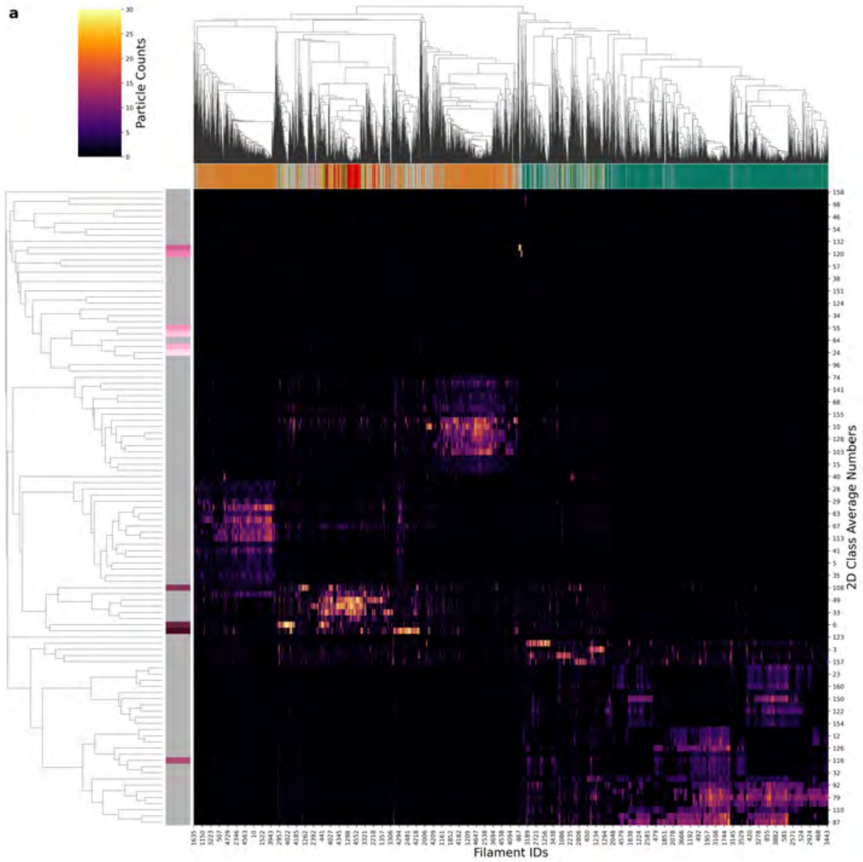

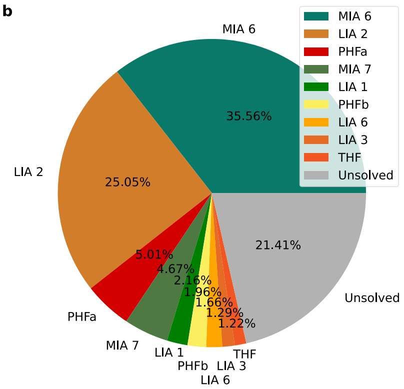

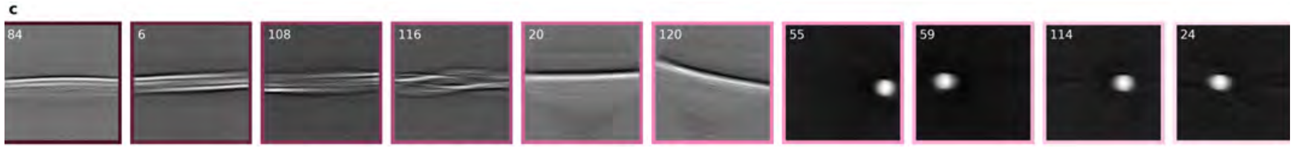

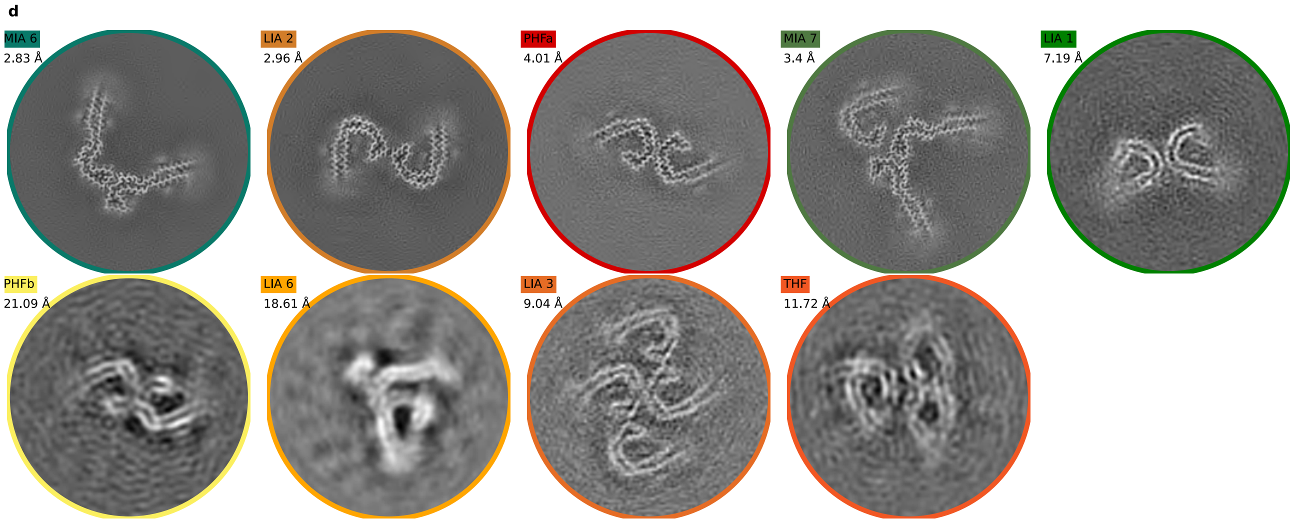

Supplementary Figure 7: Cryo-EM data set summary 7. Data set characteristics are specified in the table on the top right. **a** Hierarchical classification of individual filament segments according to their assigned 2D class average (vertical) and the picked filament ID(horizontal). **b** Pie chart with the relative amounts of different filament types. Grey represents unsolved filaments. Filament types are the same as in Figure 4 of the main text. Different colours represent different time points (120 min in purples; 180 min in blues; 240 min in greens; 300 min in yellows; 360 min in oranges and 720 min in reds). Structures are coloured according to the time point at which they are most abundant, averaged across all replicates. Unique names of filament types are indicated and the same names are used throughout this document. **c** 2D class averages of unsolved filaments. **d** XY-cross-sections, with a projected depth of approximately 4.7 Angstrom or each filament type.

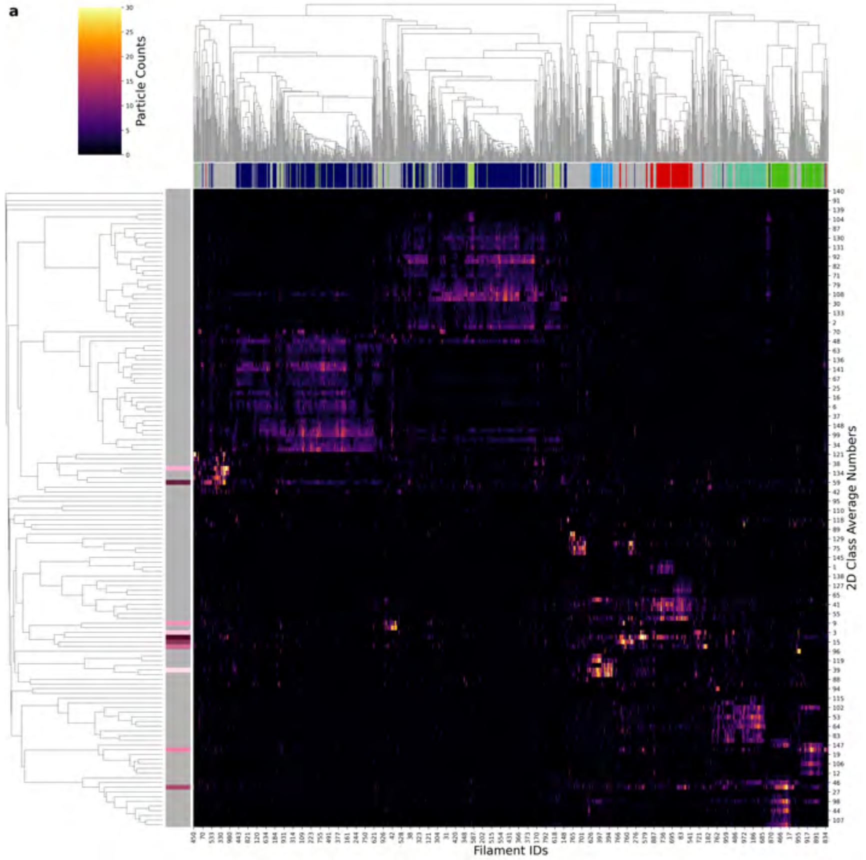

Condition	PHF
Timepoint	240 minutes
Replicate	3
Picking	Manual
Micrographs	2320
Extracted Particles	80292
Pixel Size	0.727 Å
Classified Particles	80292

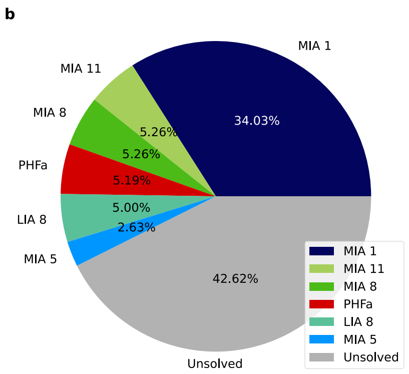

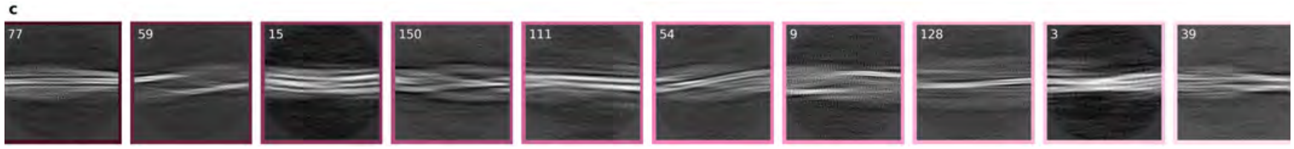

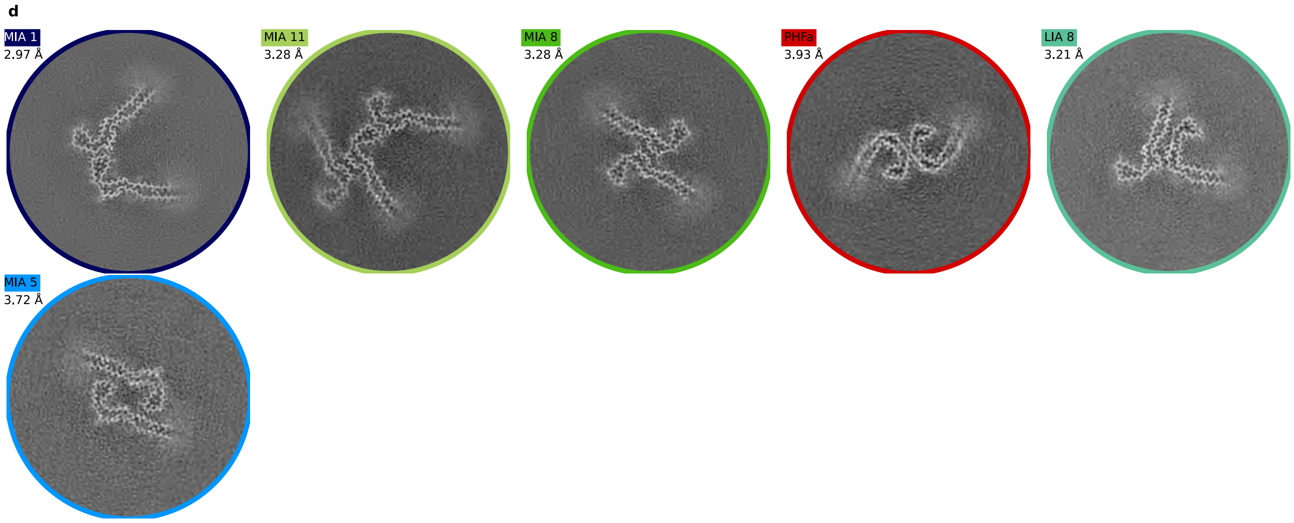

Supplementary Figure 8: Cryo-EM data set summary 8. Data set characteristics are specified in the table on the top right. **a** Hierarchical classification of individual filament segments according to their assigned 2D class average (vertical) and the picked filament ID(horizontal). **b** Pie chart with the relative amounts of different filament types. Grey represents unsolved filaments. Filament types are the same as in Figure 4 of the main text. Different colours represent different time points (120 min in purples; 180 min in blues; 240 min in greens; 300 min in yellows; 360 min in oranges and 720 min in reds). Structures are coloured according to the time point at which they are most abundant, averaged across all replicates. Unique names of filament types are indicated and the same names are used throughout this document. **c** 2D class averages of unsolved filaments. **d** XY-cross-sections, with a projected depth of approximately 4.7 Angstrom or each filament type.

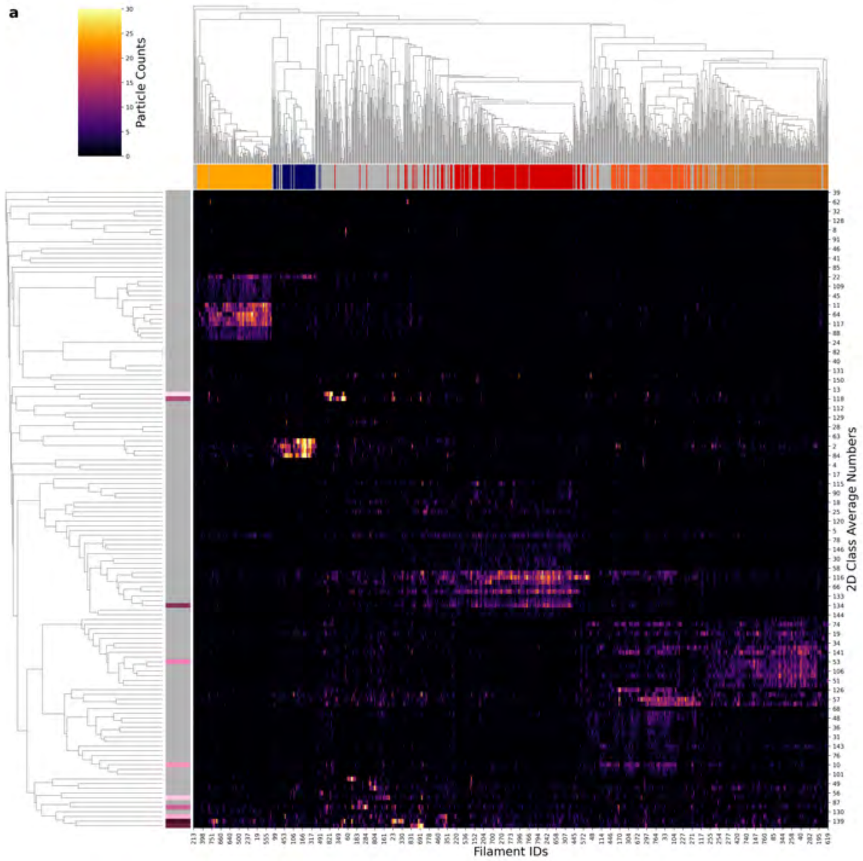

Condition	PHF
Timepoint	240 minutes
Replicate	4
Picking	Manual
Micrographs	2560
Extracted Particles	72343
Pixel Size	0.727 Å
Classified Particles	72343

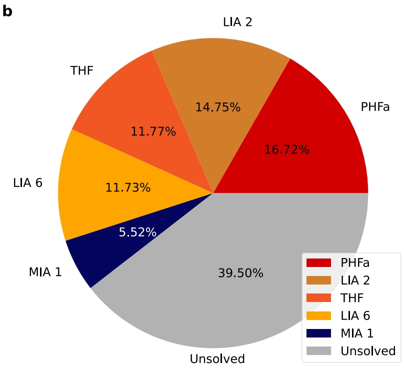

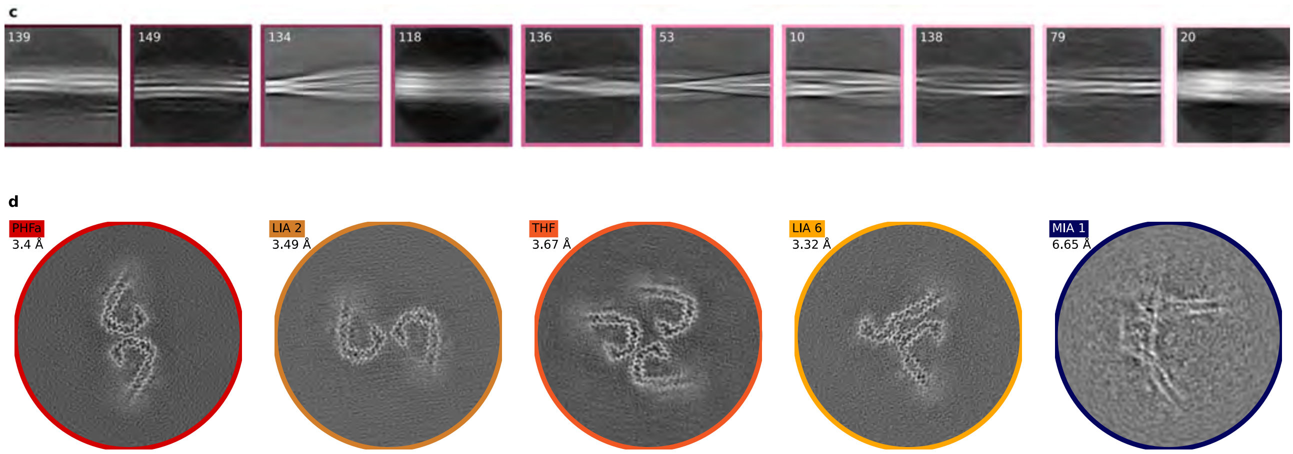

Supplementary Figure 9: Cryo-EM data set summary 9. Data set characteristics are specified in the table on the top right. **a** Hierarchical classification of individual filament segments according to their assigned 2D class average (vertical) and the picked filament ID(horizontal). **b** Pie chart with the relative amounts of different filament types. Grey represents unsolved filaments. Filament types are the same as in Figure 4 of the main text. Different colours represent different time points (120 min in purples; 180 min in blues; 240 min in greens; 300 min in yellows; 360 min in oranges and 720 min in reds). Structures are coloured according to the time point at which they are most abundant, averaged across all replicates. Unique names of filament types are indicated and the same names are used throughout this document. **c** 2D class averages of unsolved filaments. **d** XY-cross-sections, with a projected depth of approximately 4.7 Angstrom or each filament type.

a

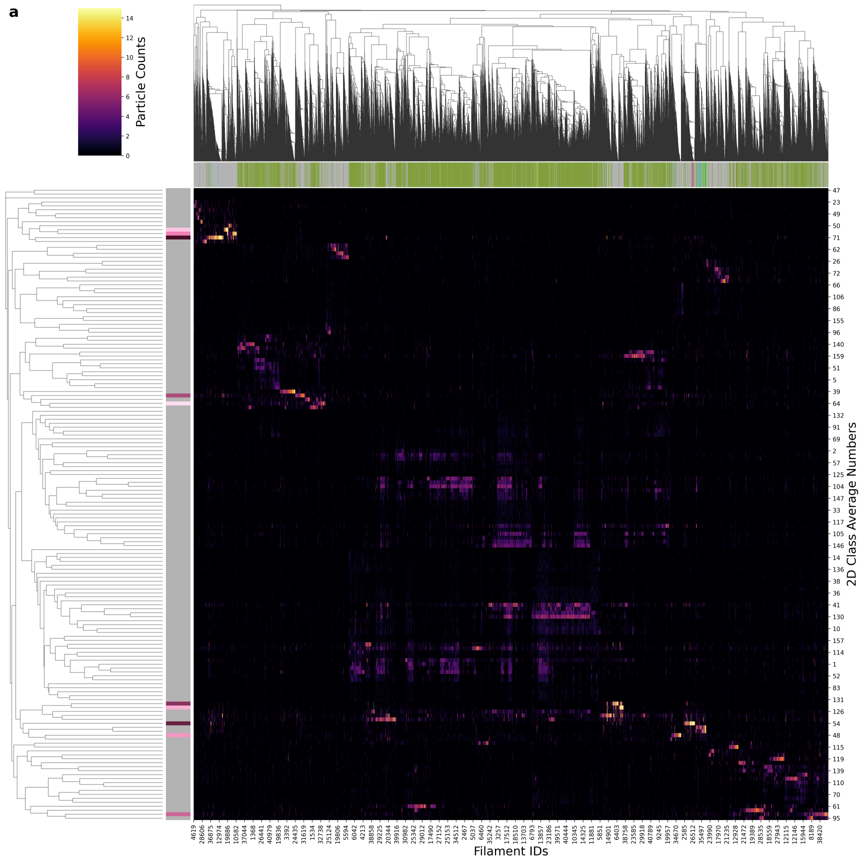

**Condition**	PHF
**Timepoint**	240 minutes
**Replicate**	5
**Picking**	Auto
**Micrographs**	2228
**Extracted Particles**	1178114
**Pixel Size**	0.727 Å
**Classified Particles**	1178114

b

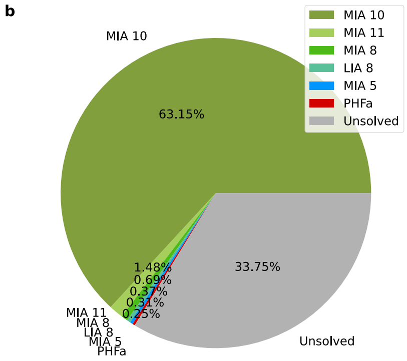

c

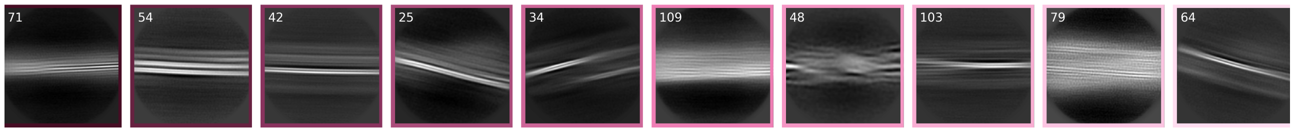

d

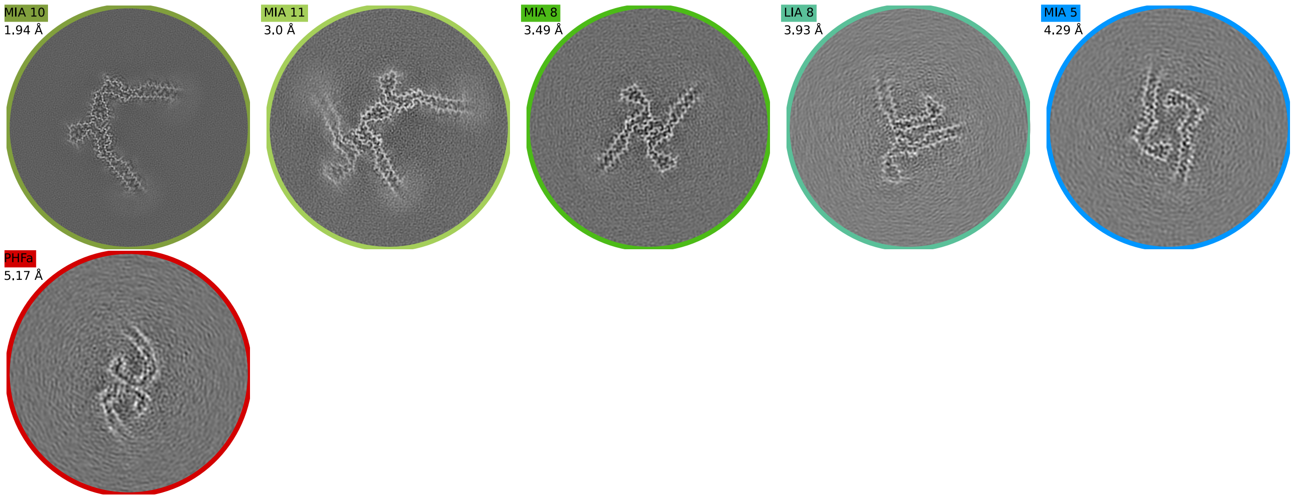

Supplementary Figure 10: Cryo-EM data set summary 10. Data set characteristics are specified in the table on the top right. **a** Hierarchical classification of individual filament segments according to their assigned 2D class average (vertical) and the picked filament ID(horizontal). **b** Pie chart with the relative amounts of different filament types. Grey represents unsolved filaments. Filament types are the same as in Figure 4 of the main text. Different colours represent different time points (120 min in purples; 180 min in blues; 240 min in greens; 300 min in yellows; 360 min in oranges and 720 min in reds). Structures are coloured according to the time point at which they are most abundant, averaged across all replicates. Unique names of filament types are indicated and the same names are used throughout this document. **c** 2D class averages of unsolved filaments. **d** XY-cross-sections, with a projected depth of approximately 4.7 Angstrom or each filament type.

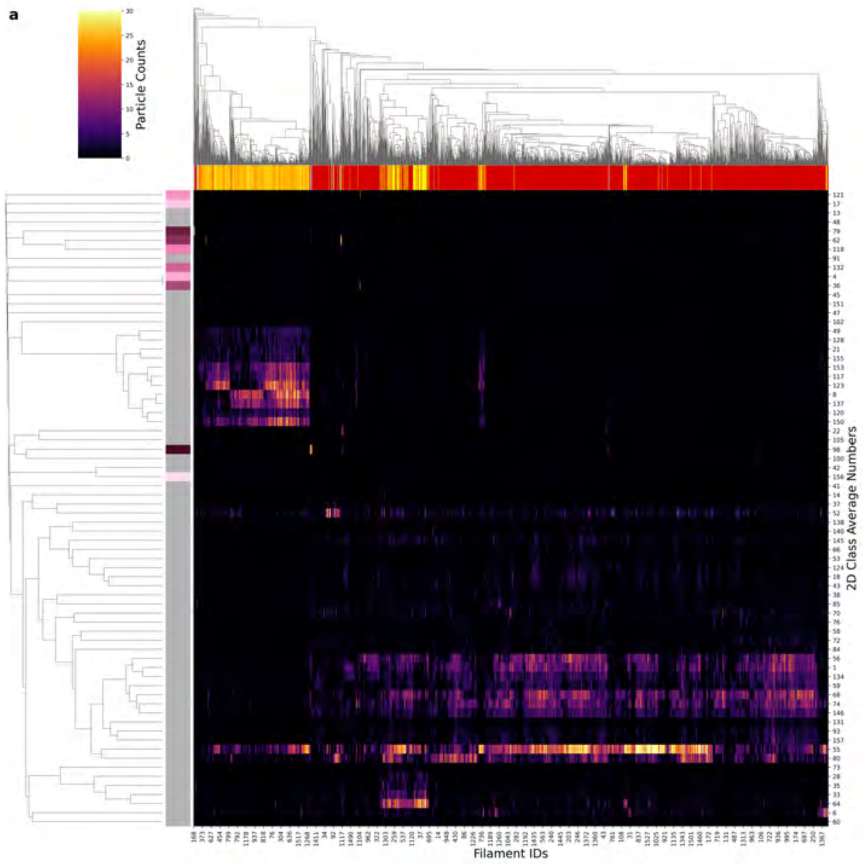

Condition	PHF
Timepoint	300 minutes
Replicate	2
Picking	Manual
Micrographs	4083
Extracted Particles	103645
Pixel Size	0.824 Å
Classified Particles	103645

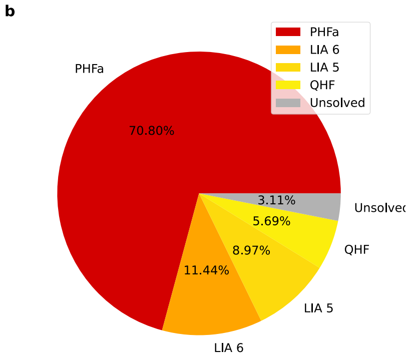

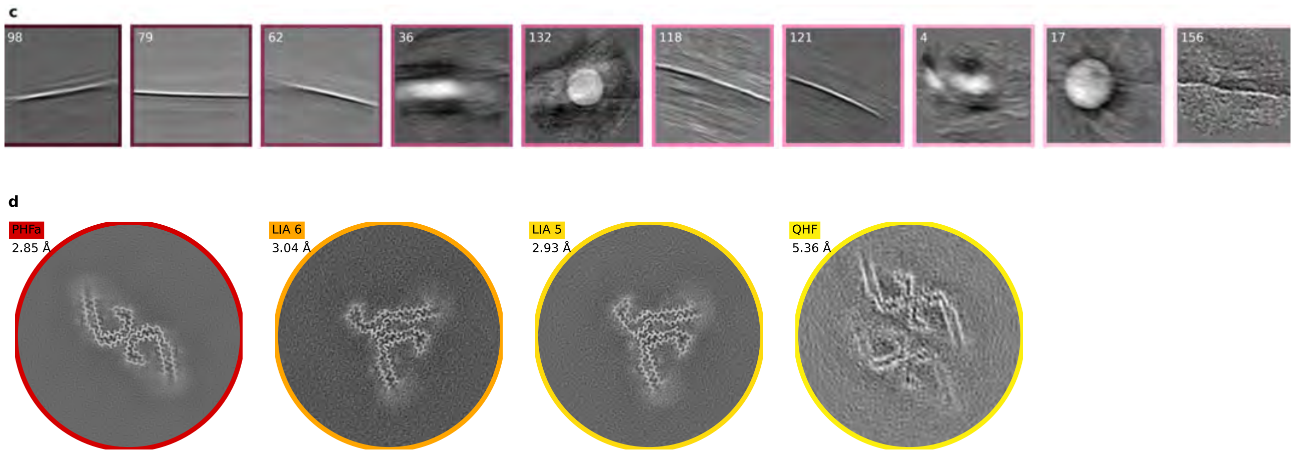

Supplementary Figure 11: Cryo-EM data set summary 11. Data set characteristics are specified in the table on the top right. **a** Hierarchical classification of individual filament segments according to their assigned 2D class average (vertical) and the picked filament ID(horizontal). **b** Pie chart with the relative amounts of different filament types. Grey represents unsolved filaments. Filament types are the same as in Figure 4 of the main text. Different colours represent different time points (120 min in purples; 180 min in blues; 240 min in greens; 300 min in yellows; 360 min in oranges and 720 min in reds). Structures are coloured according to the time point at which they are most abundant, averaged across all replicates. Unique names of filament types are indicated and the same names are used throughout this document. **c** 2D class averages of unsolved filaments. **d** XY-cross-sections, with a projected depth of approximately 4.7 Angstrom or each filament type.

a

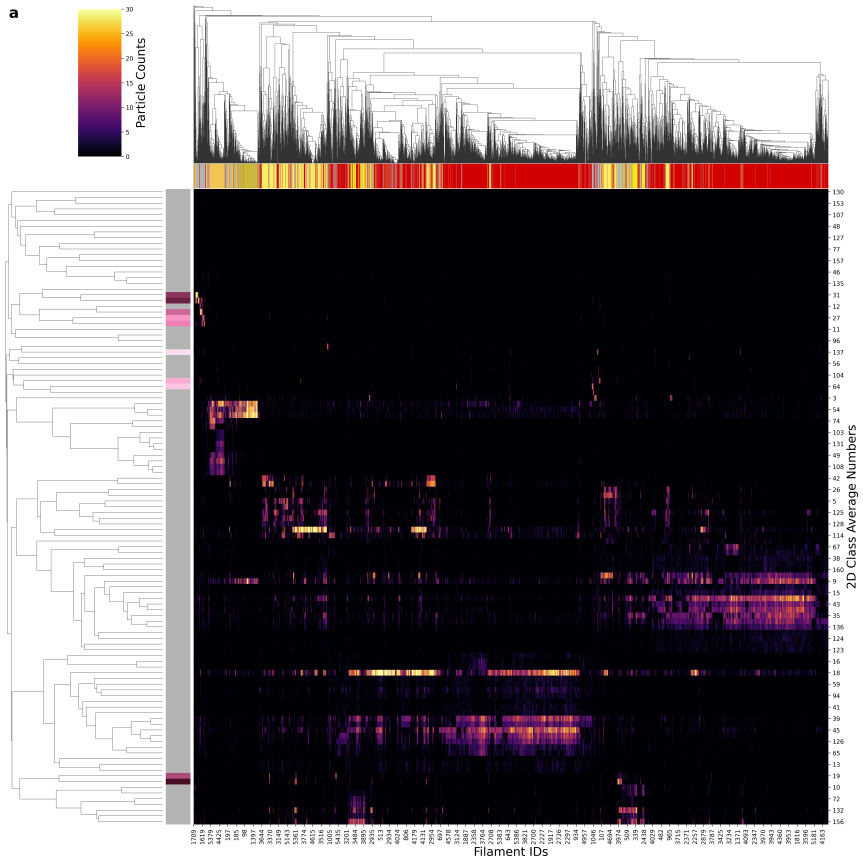

Condition	PHF
Timepoint	300 minutes
Replicate	3
Picking	Manual
Micrographs	3923
Extracted Particles	441748
Pixel Size	0.824 Å
Classified Particles	441748

b

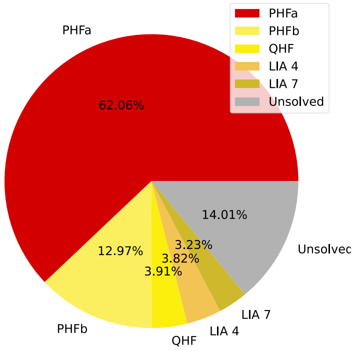

c

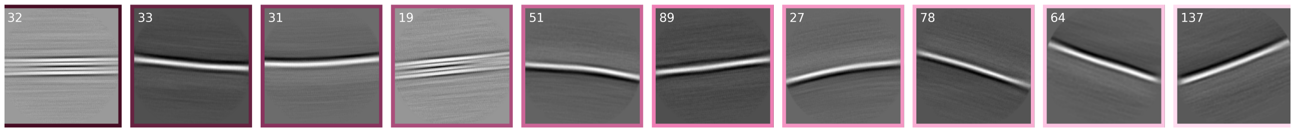

d

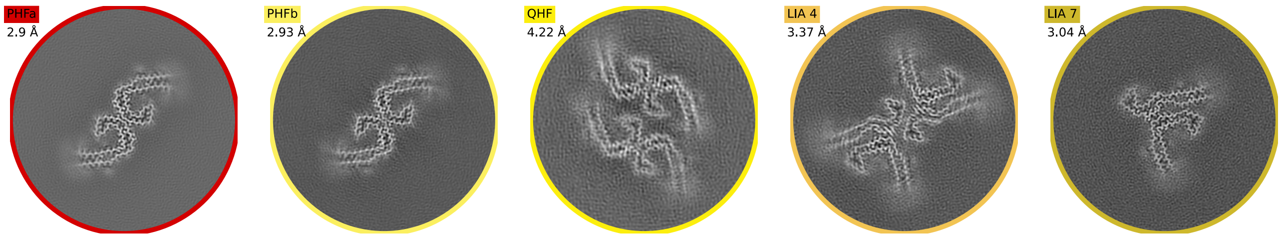

Supplementary Figure 12: Cryo-EM data set summary 12. Data set characteristics are specified in the table on the top right. **a** Hierarchical classification of individual filament segments according to their assigned 2D class average (vertical) and the picked filament ID(horizontal). **b** Pie chart with the relative amounts of different filament types. Grey represents unsolved filaments. Filament types are the same as in Figure 4 of the main text. Different colours represent different time points (120 min in purples; 180 min in blues; 240 min in greens; 300 min in yellows; 360 min in oranges and 720 min in reds). Structures are coloured according to the time point at which they are most abundant, averaged across all replicates. Unique names of filament types are indicated and the same names are used throughout this document. **c** 2D class averages of unsolved filaments. **d** XY-cross-sections, with a projected depth of approximately 4.7 Angstrom or each filament type.

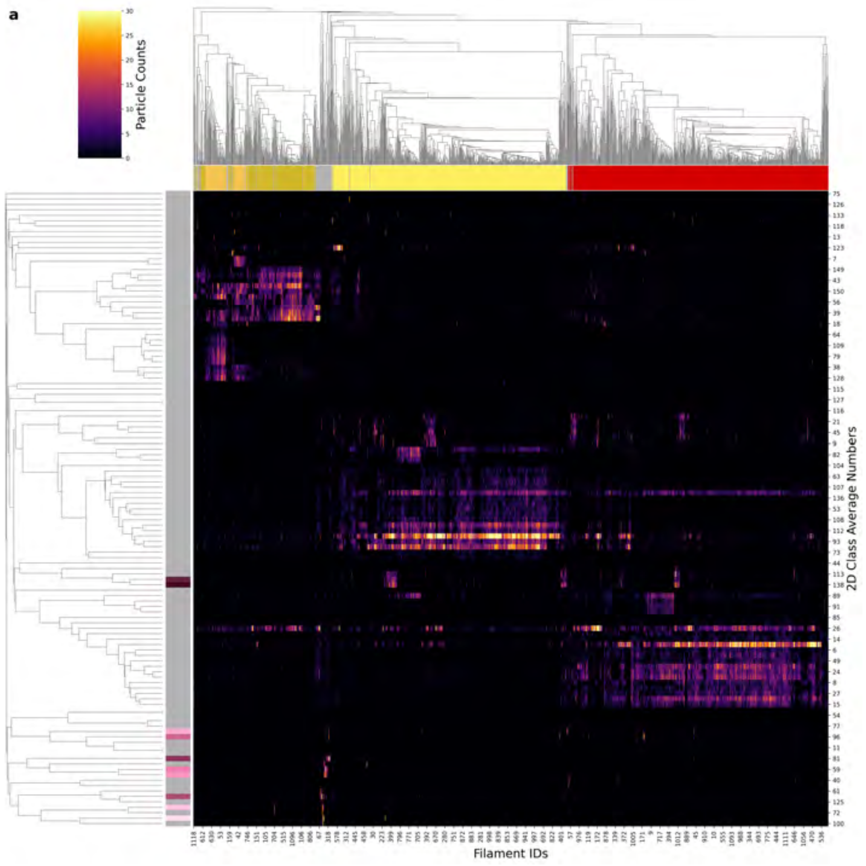

Condition	PHF
Timepoint	300 minutes
Replicate	4
Picking	Manual
Micrographs	3923
Extracted Particles	100371
Pixel Size	0.824 Å
Classified Particles	100371

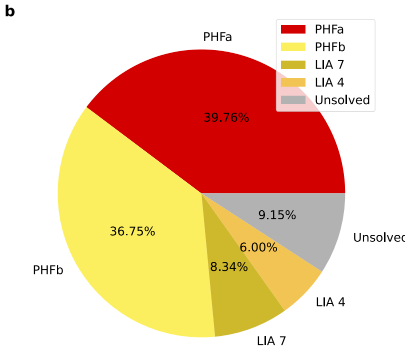

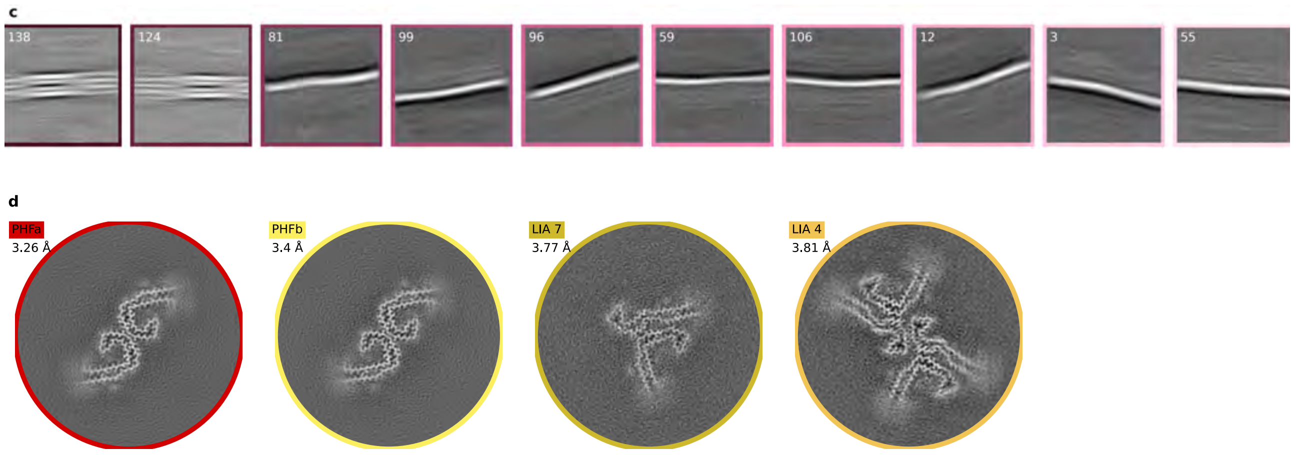

Supplementary Figure 13: Cryo-EM data set summary 13. Data set characteristics are specified in the table on the top right. **a** Hierarchical classification of individual filament segments according to their assigned 2D class average (vertical) and the picked filament ID(horizontal). **b** Pie chart with the relative amounts of different filament types. Grey represents unsolved filaments. Filament types are the same as in Figure 4 of the main text. Different colours represent different time points (120 min in purples; 180 min in blues; 240 min in greens; 300 min in yellows; 360 min in oranges and 720 min in reds). Structures are coloured according to the time point at which they are most abundant, averaged across all replicates. Unique names of filament types are indicated and the same names are used throughout this document. **c** 2D class averages of unsolved filaments. **d** XY-cross-sections, with a projected depth of approximately 4.7 Angstrom or each filament type.

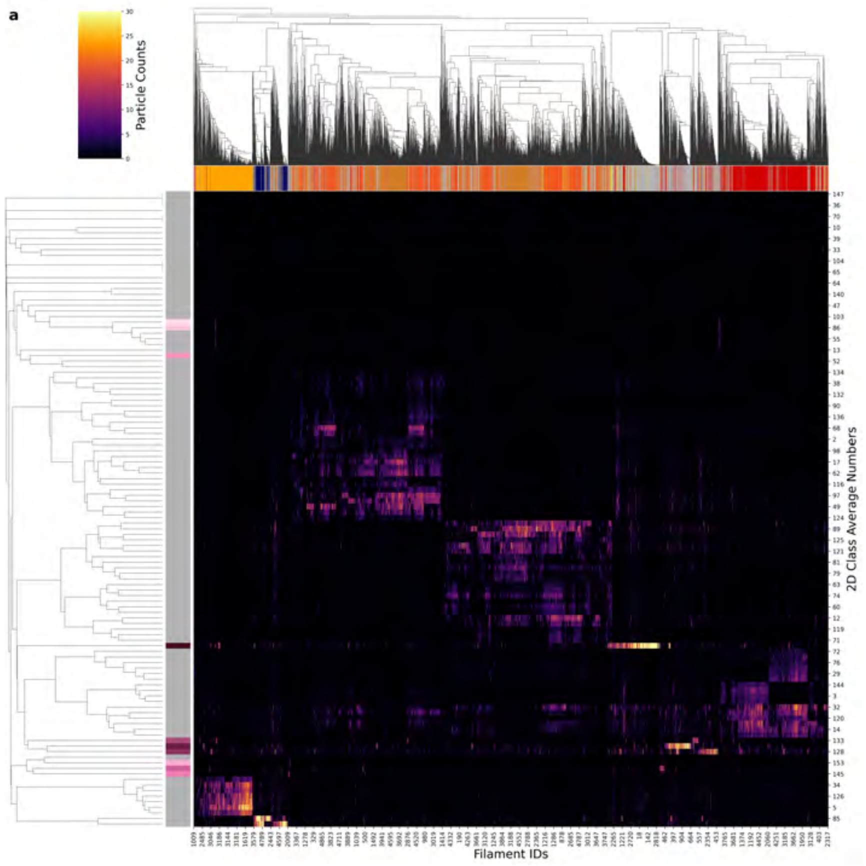

Condition	PHF
Timepoint	360 minutes
Replicate	1
Picking	Manual
Micrographs	2562
Extracted Particles	337152
Pixel Size	0.727 Å
Classified Particles	337152

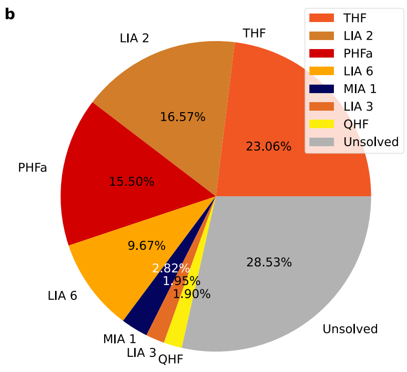

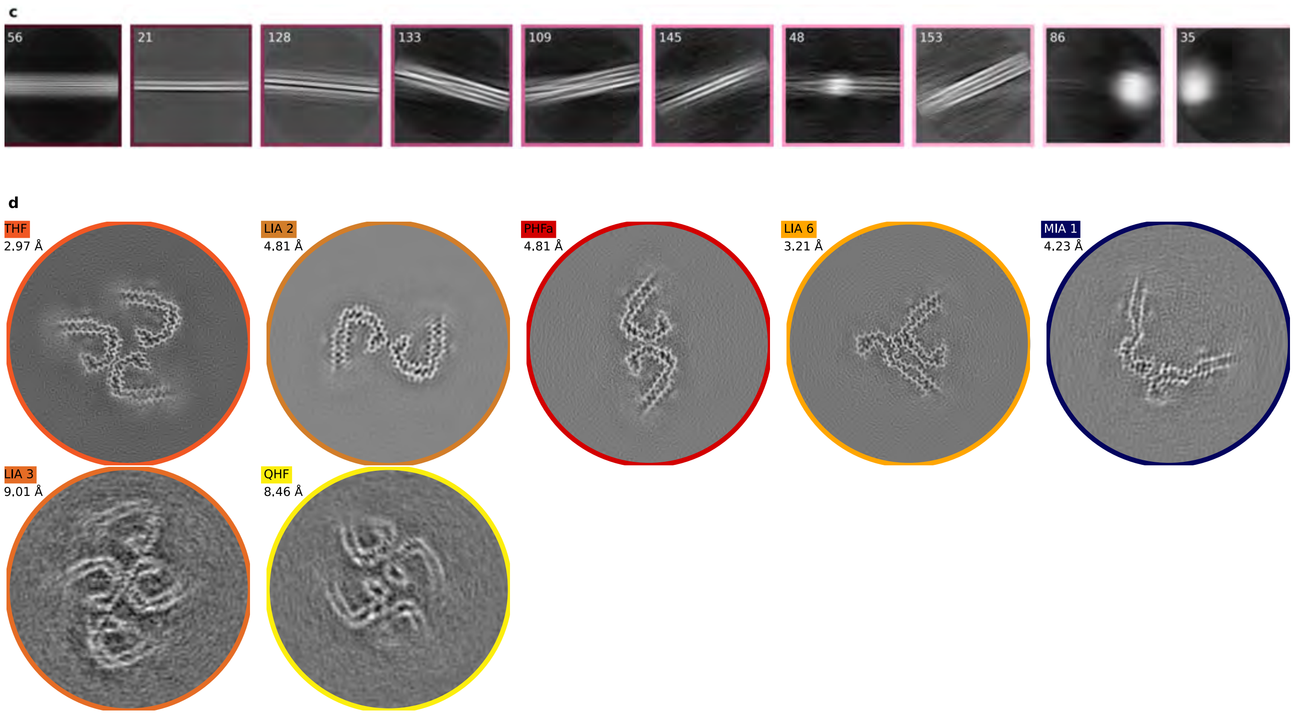

Supplementary Figure 14: Cryo-EM data set summary 14. Data set characteristics are specified in the table on the top right. **a** Hierarchical classification of individual filament segments according to their assigned 2D class average (vertical) and the picked filament ID(horizontal). **b** Pie chart with the relative amounts of different filament types. Grey represents unsolved filaments. Filament types are the same as in Figure 4 of the main text. Different colours represent different time points (120 min in purples; 180 min in blues; 240 min in greens; 300 min in yellows; 360 min in oranges and 720 min in reds). Structures are coloured according to the time point at which they are most abundant, averaged across all replicates. Unique names of filament types are indicated and the same names are used throughout this document. **c** 2D class averages of unsolved filaments. **d** XY-cross-sections, with a projected depth of approximately 4.7 Angstrom or each filament type. 14

Condition	PHF
Timepoint	360 minutes
Replicate	2
Picking	Manual
Micrographs	4519
Extracted Particles	178033
Pixel Size	0.824 Å
Classified Particles	178033

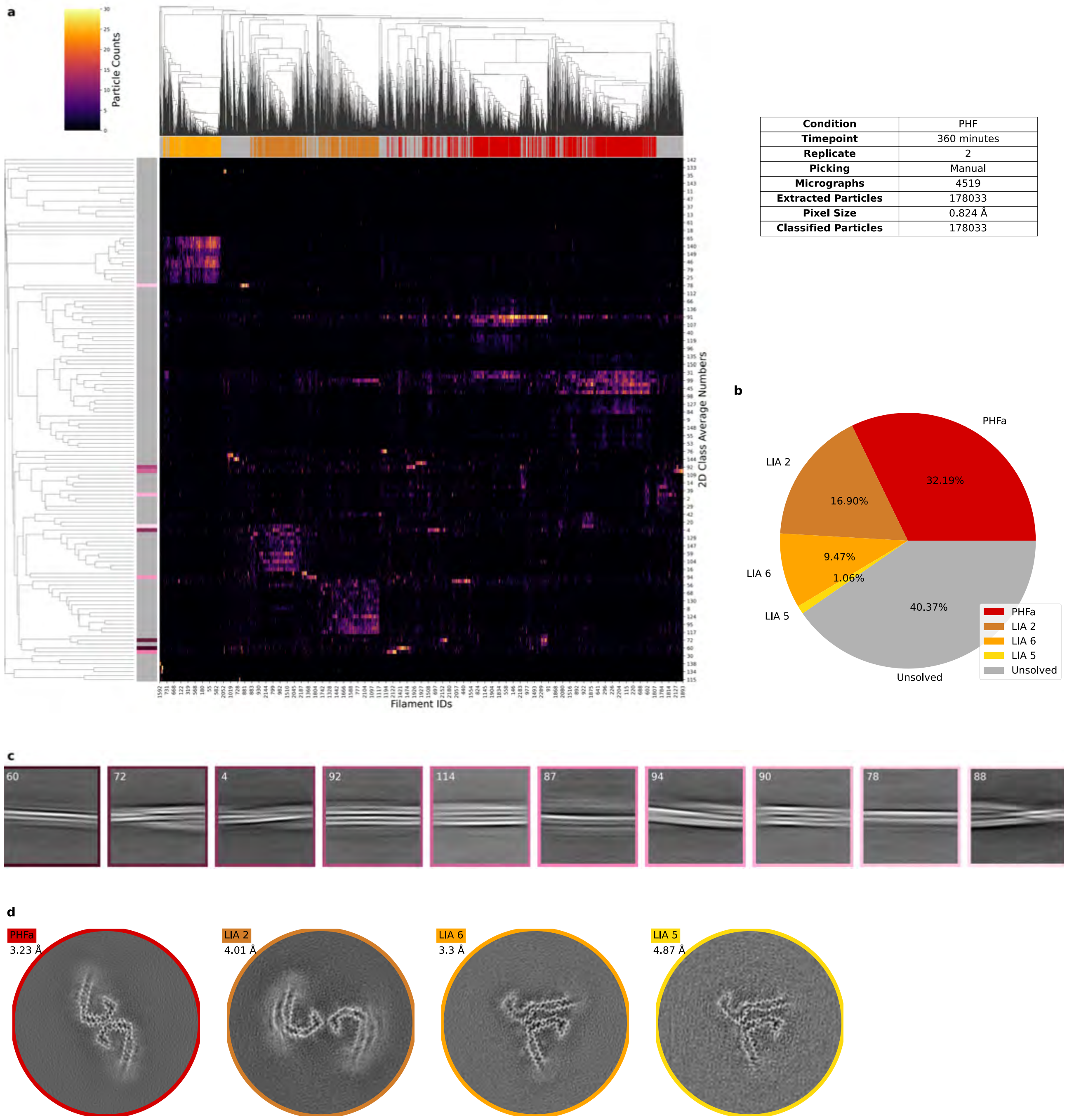

Supplementary Figure 15: Cryo-EM data set summary 15. Data set characteristics are specified in the table on the top right. **a** Hierarchical classification of individual filament segments according to their assigned 2D class average (vertical) and the picked filament ID(horizontal). **b** Pie chart with the relative amounts of different filament types. Grey represents unsolved filaments. Filament types are the same as in Figure 4 of the main text. Different colours represent different time points (120 min in purples; 180 min in blues; 240 min in greens; 300 min in yellows; 360 min in oranges and 720 min in reds). Structures are coloured according to the time point at which they are most abundant, averaged across all replicates. Unique names of filament types are indicated and the same names are used throughout this document. **c** 2D class averages of unsolved filaments. **d** XY-cross-sections, with a projected depth of approximately 4.7 Angstrom or each filament type.

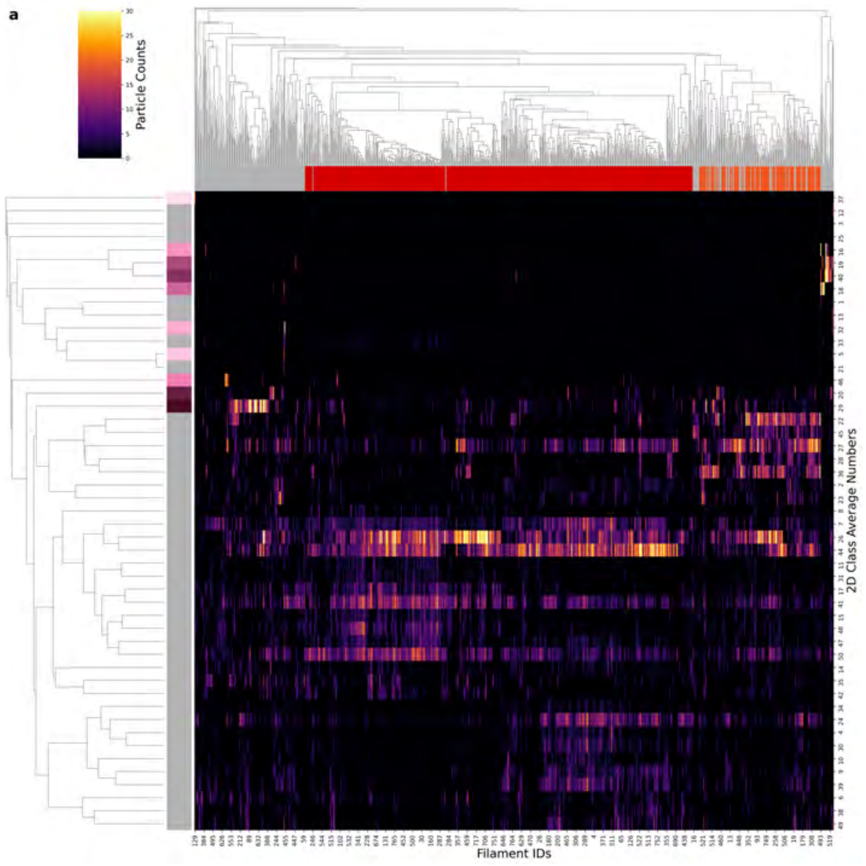

Condition	PHF
Timepoint	360 minutes
Replicate	3
Picking	Manual
Micrographs	1063
Extracted Particles	54253
Pixel Size	0.824 Å
Classified Particles	54253

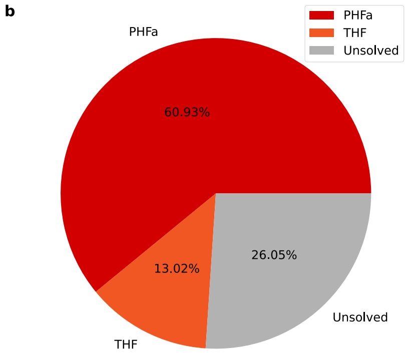

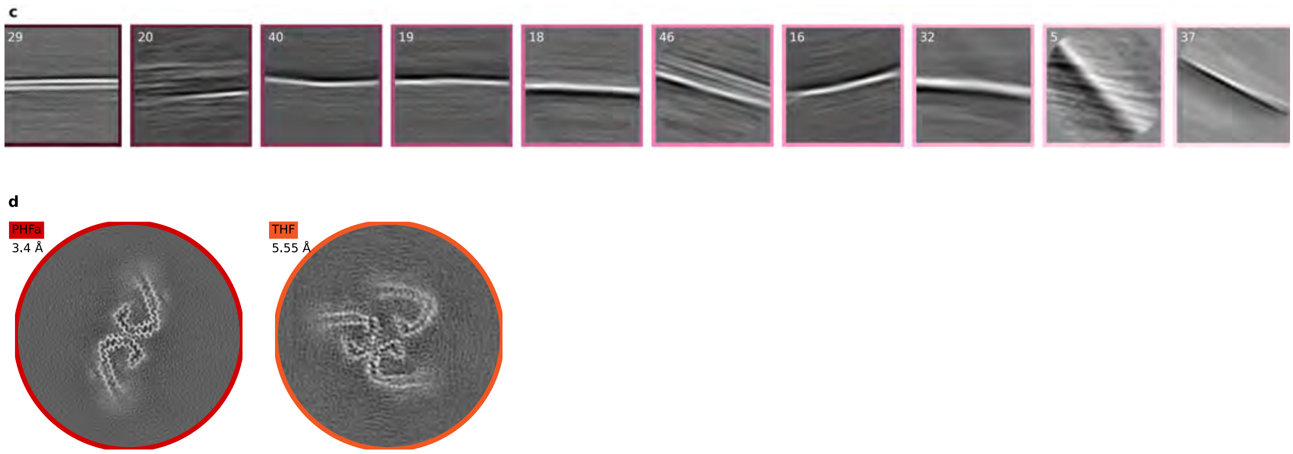

Supplementary Figure 16: Cryo-EM data set summary 16. Data set characteristics are specified in the table on the top right. **a** Hierarchical classification of individual filament segments according to their assigned 2D class average (vertical) and the picked filament ID(horizontal). **b** Pie chart with the relative amounts of different filament types. Grey represents unsolved filaments. Filament types are the same as in Figure 4 of the main text. Different colours represent different time points (120 min in purples; 180 min in blues; 240 min in greens; 300 min in yellows; 360 min in oranges and 720 min in reds). Structures are coloured according to the time point at which they are most abundant, averaged across all replicates. Unique names of filament types are indicated and the same names are used throughout this document. **c** 2D class averages of unsolved filaments. **d** XY-cross-sections, with a projected depth of approximately 4.7 Angstrom or each filament type.

a

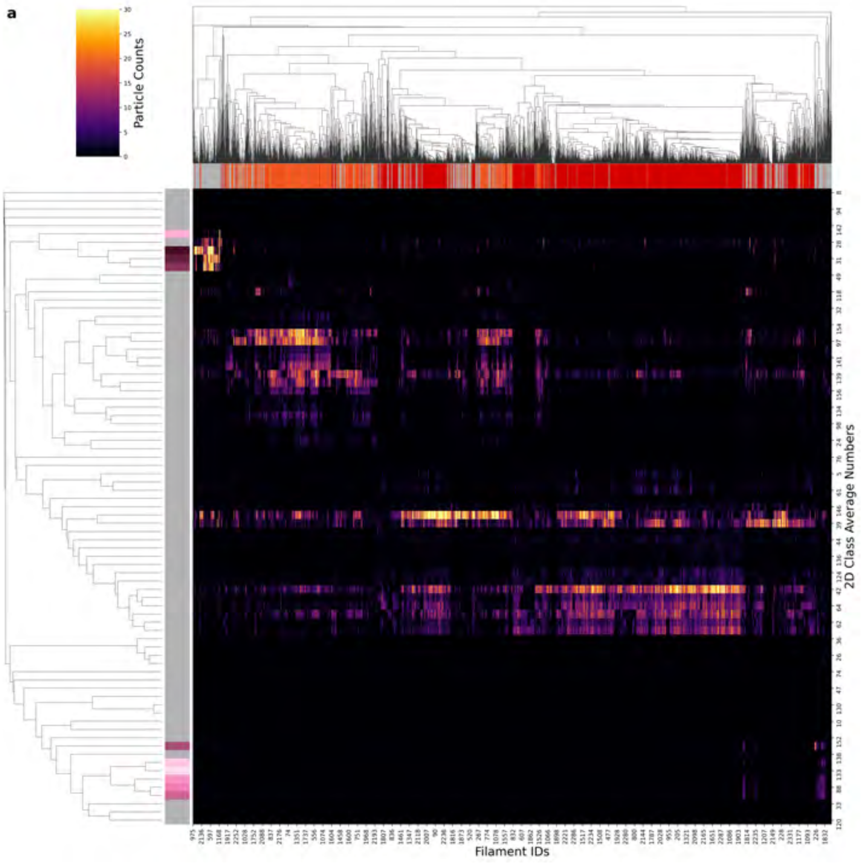

Condition	PHF
Timepoint	720 minutes
Replicate	1
Picking	Manual
Micrographs	1105
Extracted Particles	179876
Pixel Size	0.824 Å
Classified Particles	179876

b

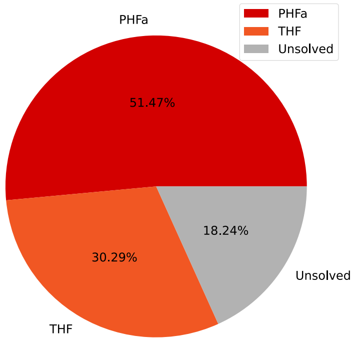

c

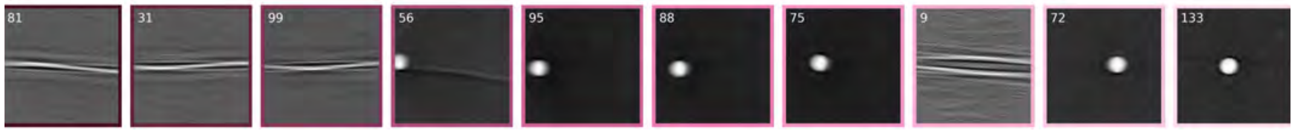

d

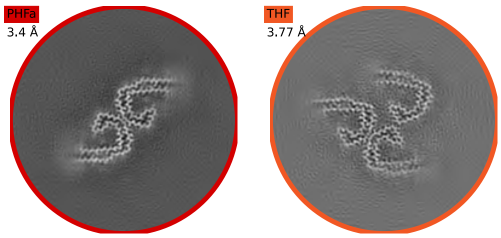

Supplementary Figure 17: Cryo-EM data set summary 17. Data set characteristics are specified in the table on the top right. **a** Hierarchical classification of individual filament segments according to their assigned 2D class average (vertical) and the picked filament ID(horizontal). **b** Pie chart with the relative amounts of different filament types. Grey represents unsolved filaments. Filament types are the same as in Figure 4 of the main text. Different colours represent different time points (120 min in purples; 180 min in blues; 240 min in greens; 300 min in yellows; 360 min in oranges and 720 min in reds). Structures are coloured according to the time point at which they are most abundant, averaged across all replicates. Unique names of filament types are indicated and the same names are used throughout this document. **c** 2D class averages of unsolved filaments. **d** XY-cross-sections, with a projected depth of approximately 4.7 Angstrom or each filament type.

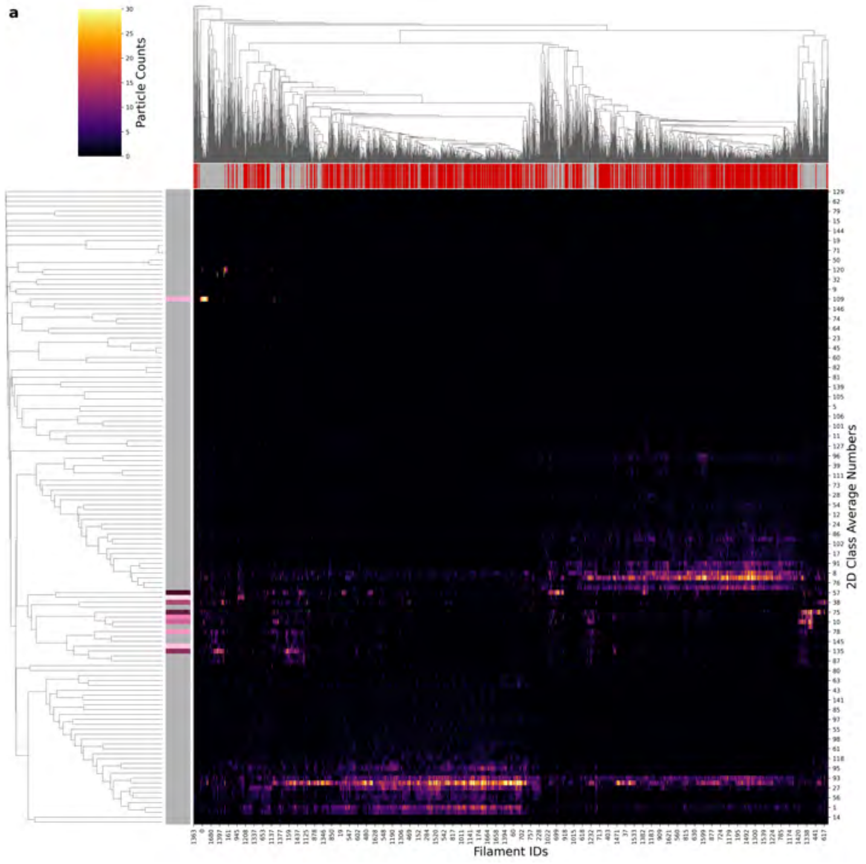

Condition	PHF
Timepoint	720 minutes
Replicate	2
Picking	Manual
Micrographs	3379
Extracted Particles	125865
Pixel Size	0.727 Å
Classified Particles	125865

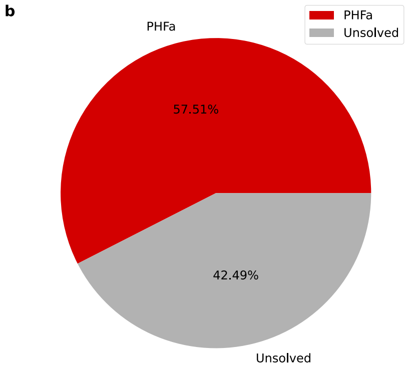

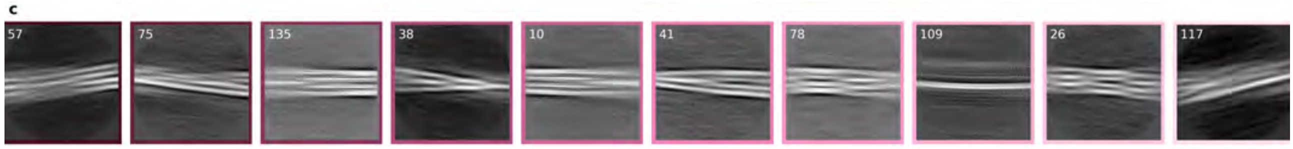

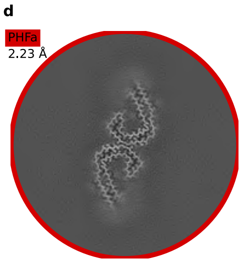

Supplementary Figure 18: Cryo-EM data set summary 18. Data set characteristics are specified in the table on the top right. **a** Hierarchical classification of individual filament segments according to their assigned 2D class average (vertical) and the picked filament ID(horizontal). **b** Pie chart with the relative amounts of different filament types. Grey represents unsolved filaments. Filament types are the same as in Figure 4 of the main text. Different colours represent different time points (120 min in purples; 180 min in blues; 240 min in greens; 300 min in yellows; 360 min in oranges and 720 min in reds). Structures are coloured according to the time point at which they are most abundant, averaged across all replicates. Unique names of filament types are indicated and the same names are used throughout this document. **c** 2D class averages of unsolved filaments. **d** XY-cross-sections, with a projected depth of approximately 4.7 Angstrom or each filament type.

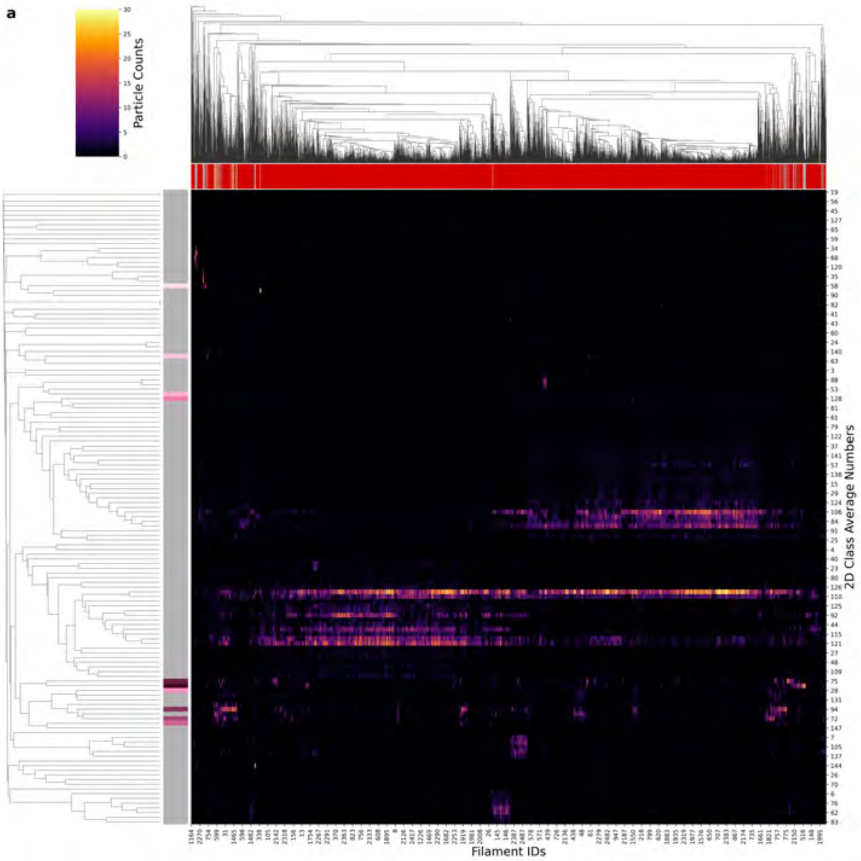

Condition	PHF
Timepoint	720 minutes
Replicate	3
Picking	Manual
Micrographs	5267
Extracted Particles	175528
Pixel Size	0.824 Å
Classified Particles	175528

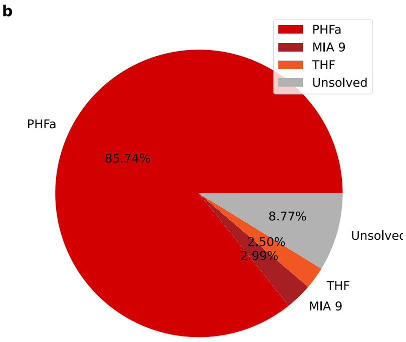

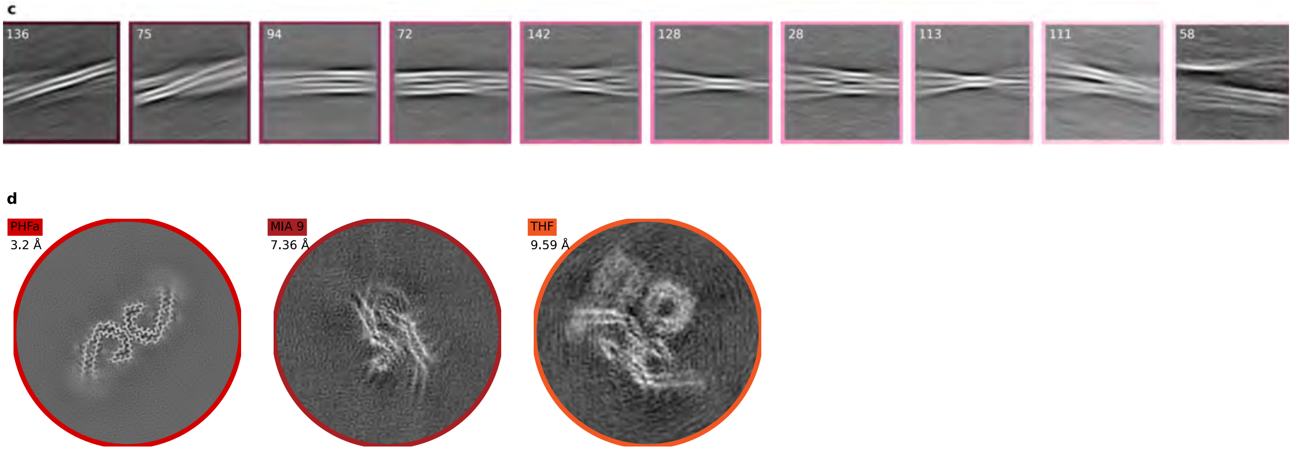

Supplementary Figure 19: Cryo-EM data set summary 19. Data set characteristics are specified in the table on the top right. **a** Hierarchical classification of individual filament segments according to their assigned 2D class average (vertical) and the picked filament ID(horizontal). **b** Pie chart with the relative amounts of different filament types. Grey represents unsolved filaments. Filament types are the same as in Figure 4 of the main text. Different colours represent different time points (120 min in purples; 180 min in blues; 240 min in greens; 300 min in yellows; 360 min in oranges and 720 min in reds). Structures are coloured according to the time point at which they are most abundant, averaged across all replicates. Unique names of filament types are indicated and the same names are used throughout this document. **c** 2D class averages of unsolved filaments. **d** XY-cross-sections, with a projected depth of approximately 4.7 Angstrom or each filament type.

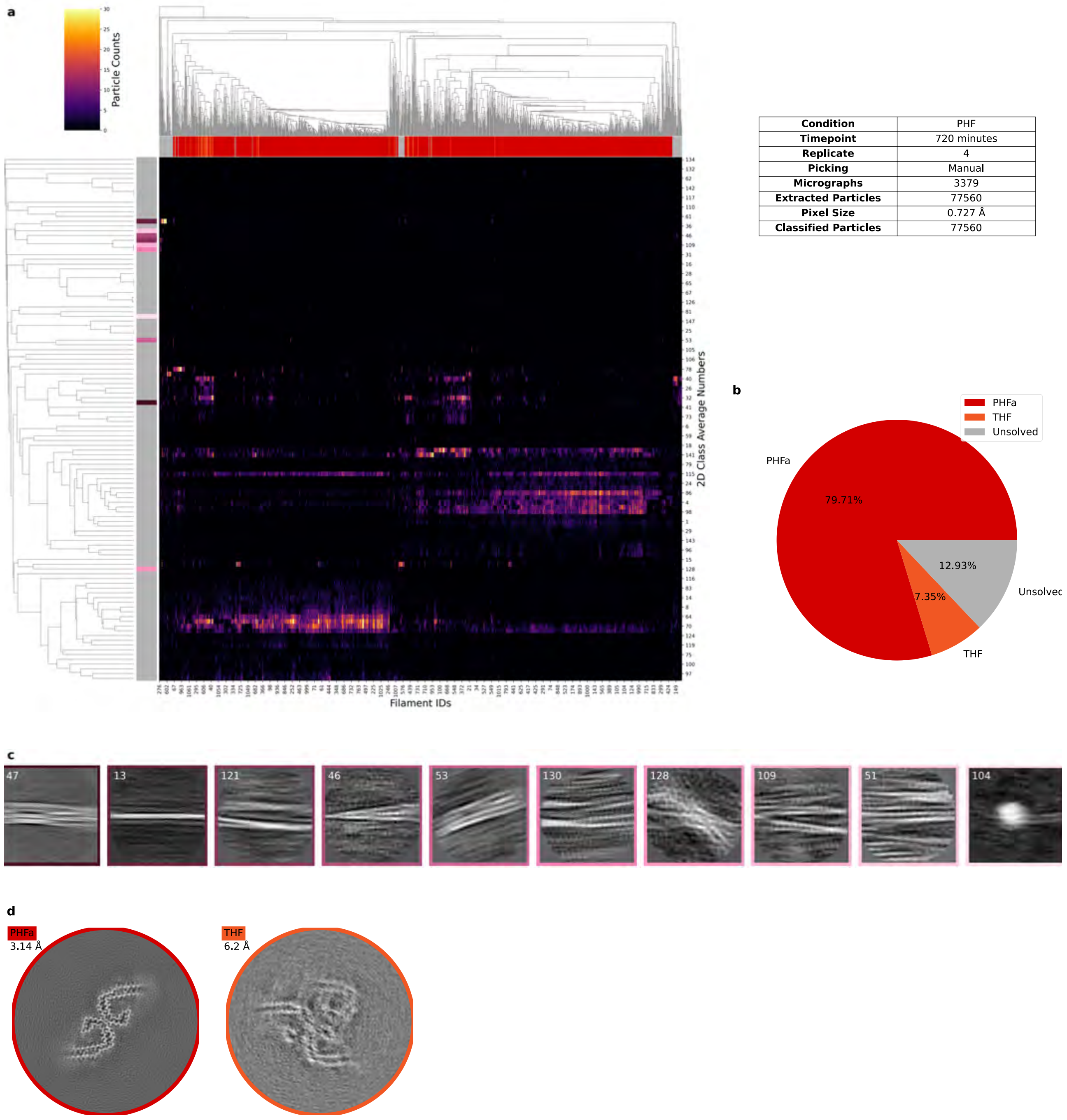

Condition	PHF
Timepoint	720 minutes
Replicate	4
Picking	Manual
Micrographs	3379
Extracted Particles	77560
Pixel Size	0.727 Å
Classified Particles	77560

Supplementary Figure 20: Cryo-EM data set summary 20. Data set characteristics are specified in the table on the top right. **a** Hierarchical classification of individual filament segments according to their assigned 2D class average (vertical) and the picked filament ID(horizontal). **b** Pie chart with the relative amounts of different filament types. Grey represents unsolved filaments. Filament types are the same as in Figure 4 of the main text. Different colours represent different time points (120 min in purples; 180 min in blues; 240 min in greens; 300 min in yellows; 360 min in oranges and 720 min in reds). Structures are coloured according to the time point at which they are most abundant, averaged across all replicates. Unique names of filament types are indicated and the same names are used throughout this document. **c** 2D class averages of unsolved filaments. **d** XY-cross-sections, with a projected depth of approximately 4.7 Angstrom or each filament type.

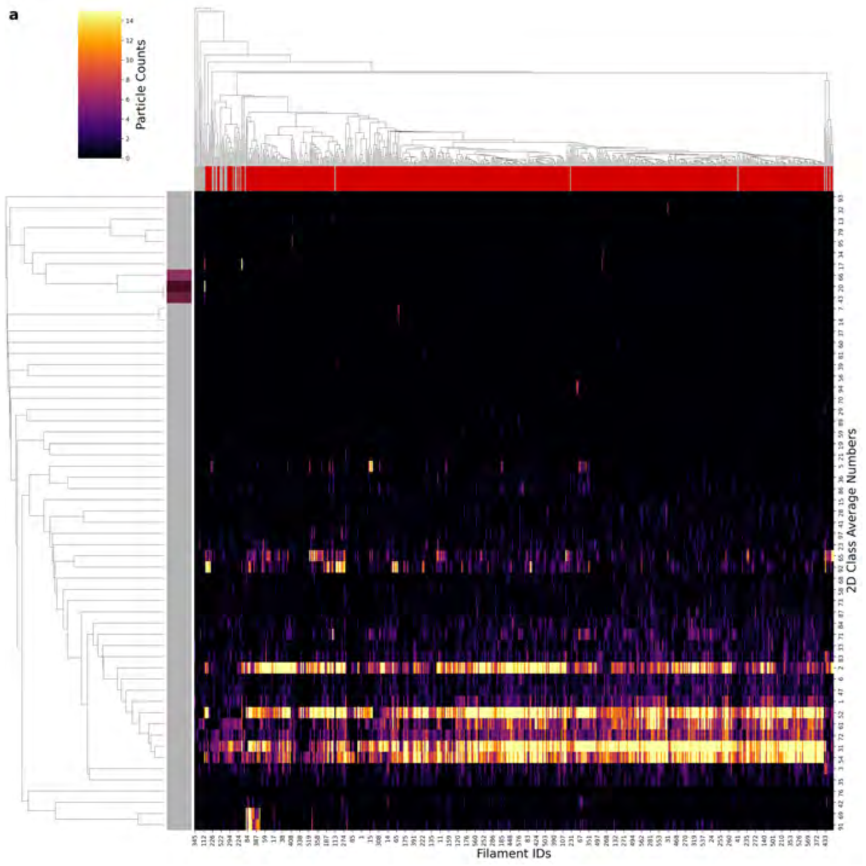

Condition	AD
Timepoint	720 minutes
Replicate	5
Picking	Manual
Micrographs	1880
Extracted Particles	46371
Pixel Size	0.824 Å
Classified Particles	46371

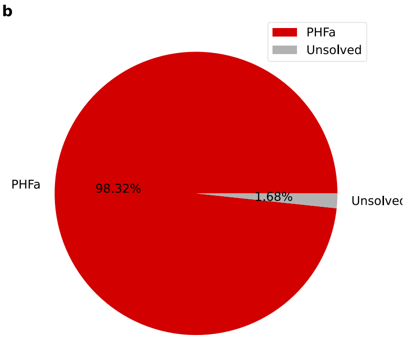

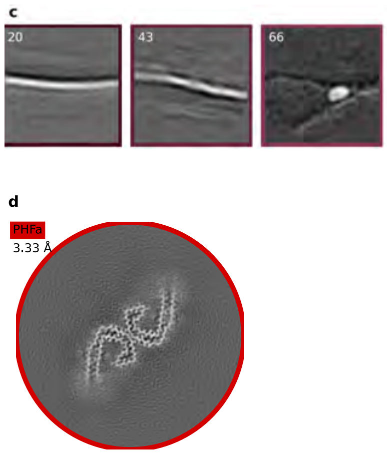

Supplementary Figure 21: Cryo-EM data set summary 21. Data set characteristics are specified in the table on the top right. **a** Hierarchical classification of individual filament segments according to their assigned 2D class average (vertical) and the picked filament ID(horizontal). **b** Pie chart with the relative amounts of different filament types. Grey represents unsolved filaments. Filament types are the same as in Figure 4 of the main text. Different colours represent different time points (120 min in purples; 180 min in blues; 240 min in greens; 300 min in yellows; 360 min in oranges and 720 min in reds). Structures are coloured according to the time point at which they are most abundant, averaged across all replicates. Unique names of filament types are indicated and the same names are used throughout this document. **c** 2D class averages of unsolved filaments. **d** XY-cross-sections, with a projected depth of approximately 4.7 Angstrom or each filament type.

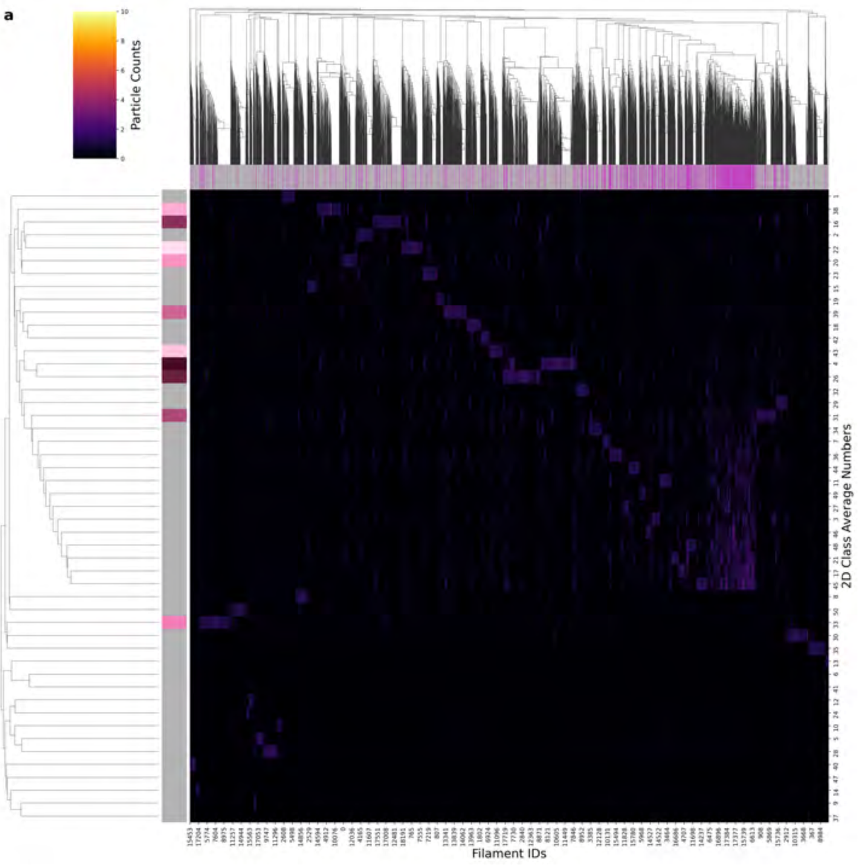

Condition	CTE
Timepoint	120 minutes
Replicate	1
Picking	Auto
Micrographs	1727
Extracted Particles	330525
Pixel Size	0.672 Å
Classified Particles	82995

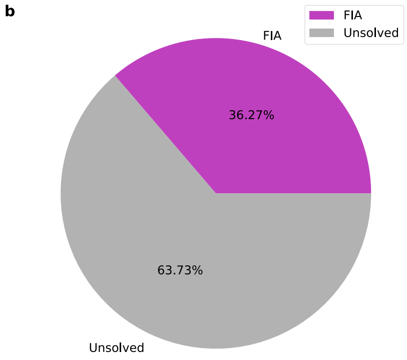

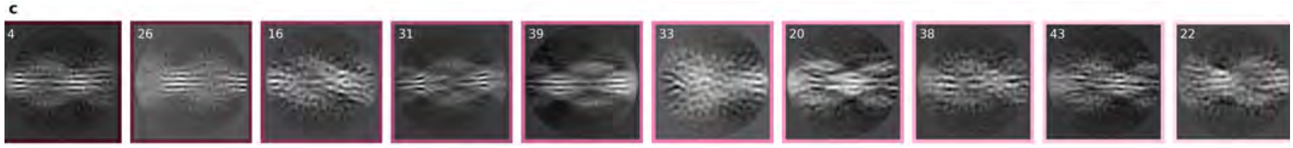

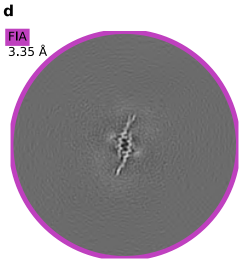

Supplementary Figure 22: Cryo-EM data set summary 22. Data set characteristics are specified in the table on the top right. **a** Hierarchical classification of individual filament segments according to their assigned 2D class average (vertical) and the picked filament ID(horizontal). **b** Pie chart with the relative amounts of different filament types. Grey represents unsolved filaments. Filament types are the same as in Figure 4 of the main text. Different colours represent different time points (120 min in purples; 180 min in blues; 240 min in greens; 300 min in yellows; 360 min in oranges and 720 min in reds). Structures are coloured according to the time point at which they are most abundant, averaged across all replicates. Unique names of filament types are indicated and the same names are used throughout this document. **c** 2D class averages of unsolved filaments. **d** XY-cross-sections, with a projected depth of approximately 4.7 Angstrom or each filament type.

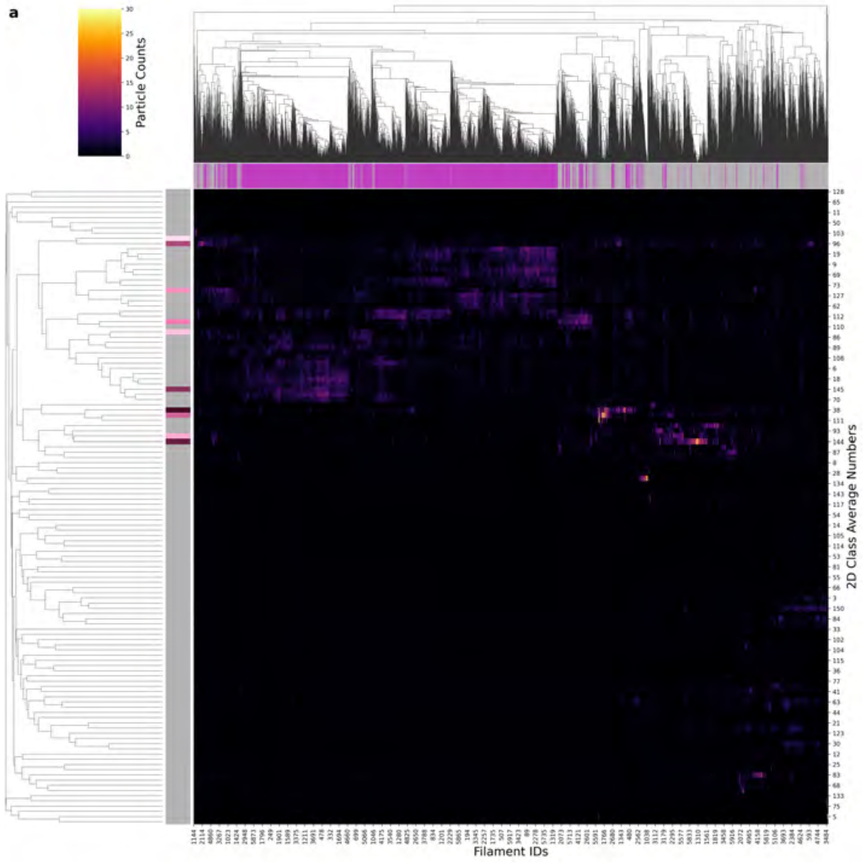

Condition	CTE
Timepoint	120 minutes
Replicate	2
Picking	Auto
Micrographs	3948
Extracted Particles	221378
Pixel Size	0.727 Å
Classified Particles	221378

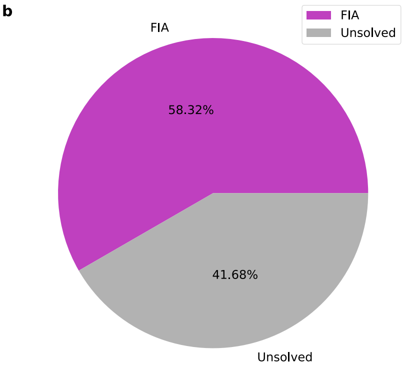

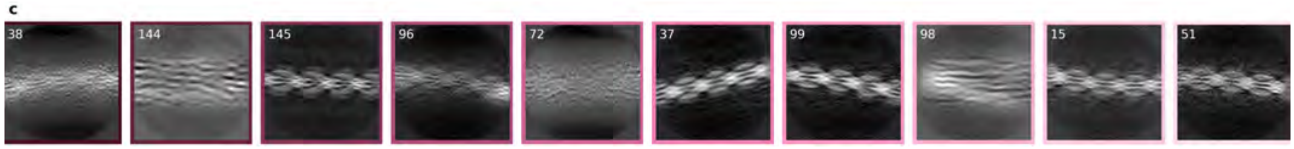

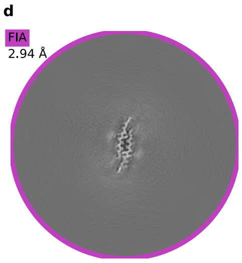

Supplementary Figure 23: Cryo-EM data set summary 23. Data set characteristics are specified in the table on the top right. **a** Hierarchical classification of individual filament segments according to their assigned 2D class average (vertical) and the picked filament ID(horizontal). **b** Pie chart with the relative amounts of different filament types. Grey represents unsolved filaments. Filament types are the same as in Figure 4 of the main text. Different colours represent different time points (120 min in purples; 180 min in blues; 240 min in greens; 300 min in yellows; 360 min in oranges and 720 min in reds). Structures are coloured according to the time point at which they are most abundant, averaged across all replicates. Unique names of filament types are indicated and the same names are used throughout this document. **c** 2D class averages of unsolved filaments. **d** XY-cross-sections, with a projected depth of approximately 4.7 Angstrom or each filament type.

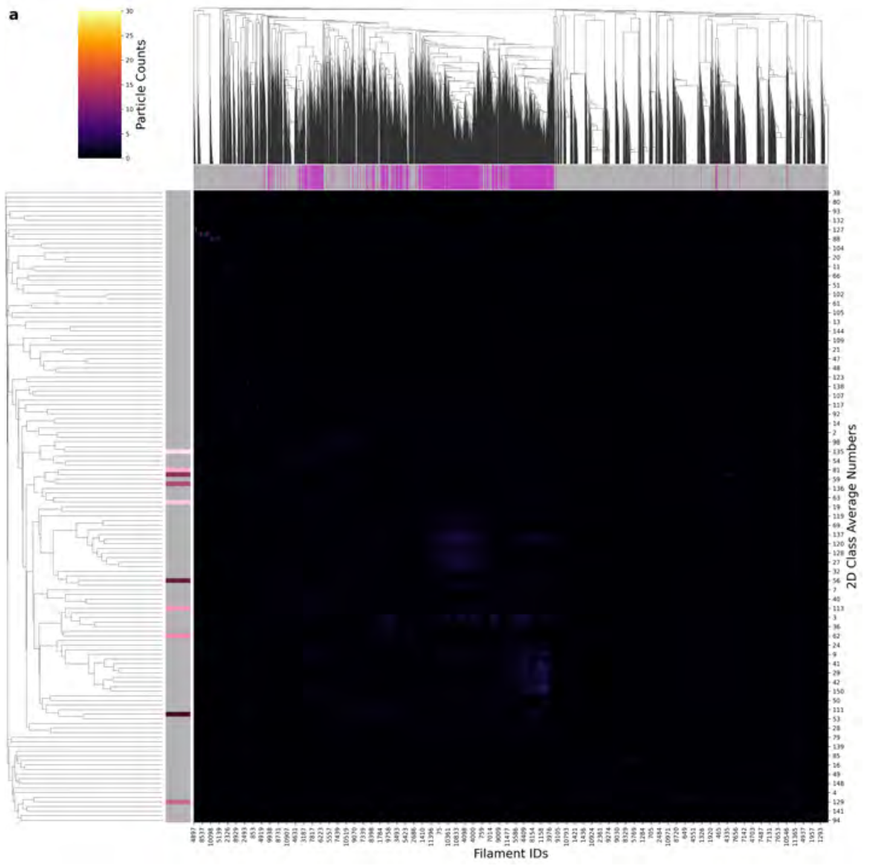

Condition	CTE
Timepoint	120 minutes
Replicate	4
Picking	Autopick
Micrographs	3940
Extracted Particles	127070
Pixel Size	0.727 Å
Classified Particles	127070

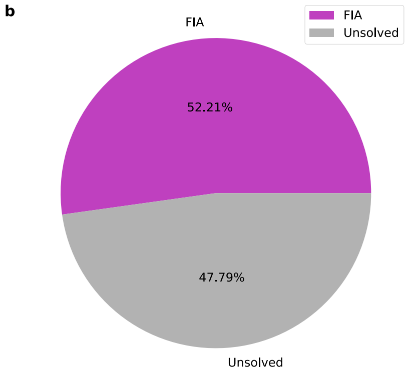

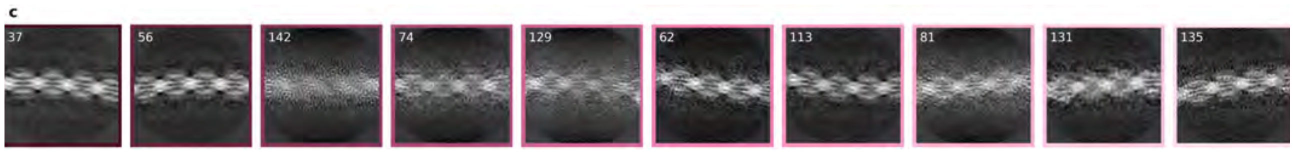

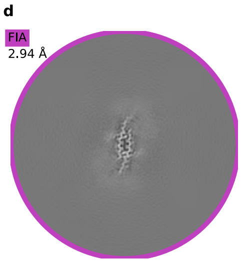

Supplementary Figure 24: Cryo-EM data set summary 24. Data set characteristics are specified in the table on the top right. **a** Hierarchical classification of individual filament segments according to their assigned 2D class average (vertical) and the picked filament ID(horizontal). **b** Pie chart with the relative amounts of different filament types. Grey represents unsolved filaments. Filament types are the same as in Figure 4 of the main text. Different colours represent different time points (120 min in purples; 180 min in blues; 240 min in greens; 300 min in yellows; 360 min in oranges and 720 min in reds). Structures are coloured according to the time point at which they are most abundant, averaged across all replicates. Unique names of filament types are indicated and the same names are used throughout this document. **c** 2D class averages of unsolved filaments. **d** XY-cross-sections, with a projected depth of approximately 4.7 Angstrom or each filament type.

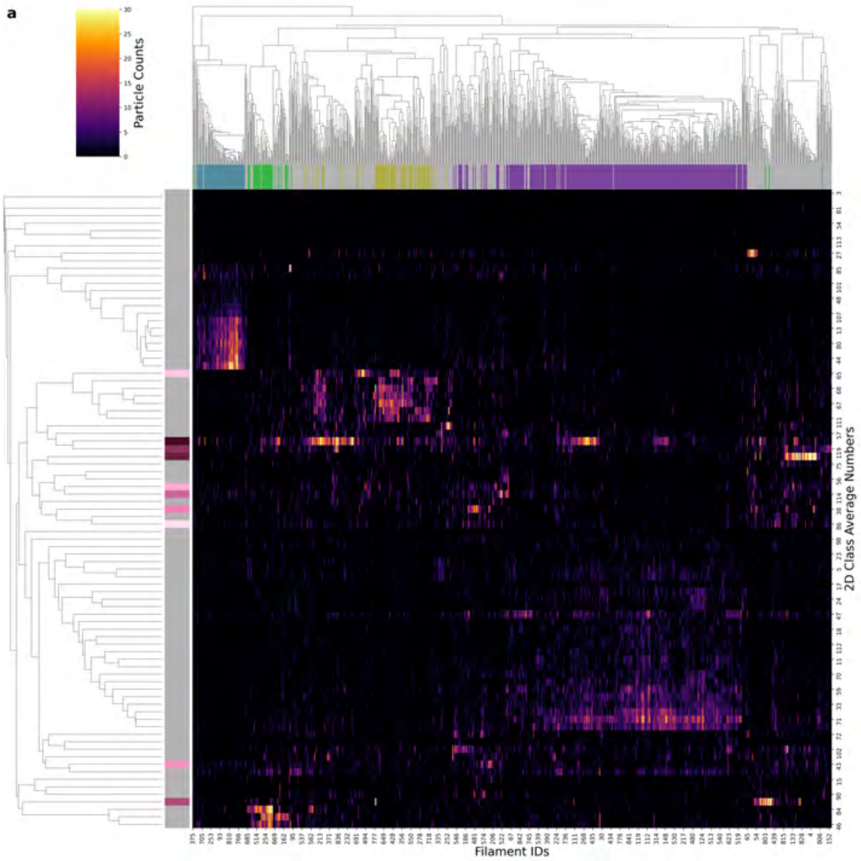

Condition	CTE
Timepoint	160 minutes
Replicate	4
Picking	Manual
Micrographs	932
Extracted Particles	59766
Pixel Size	0.824 Å
Classified Particles	59766

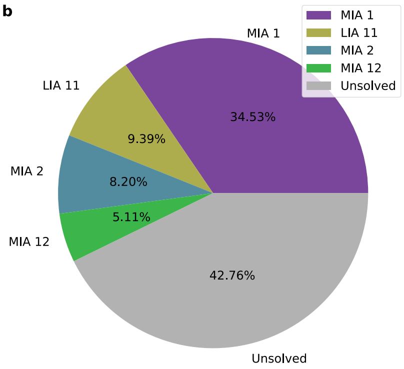

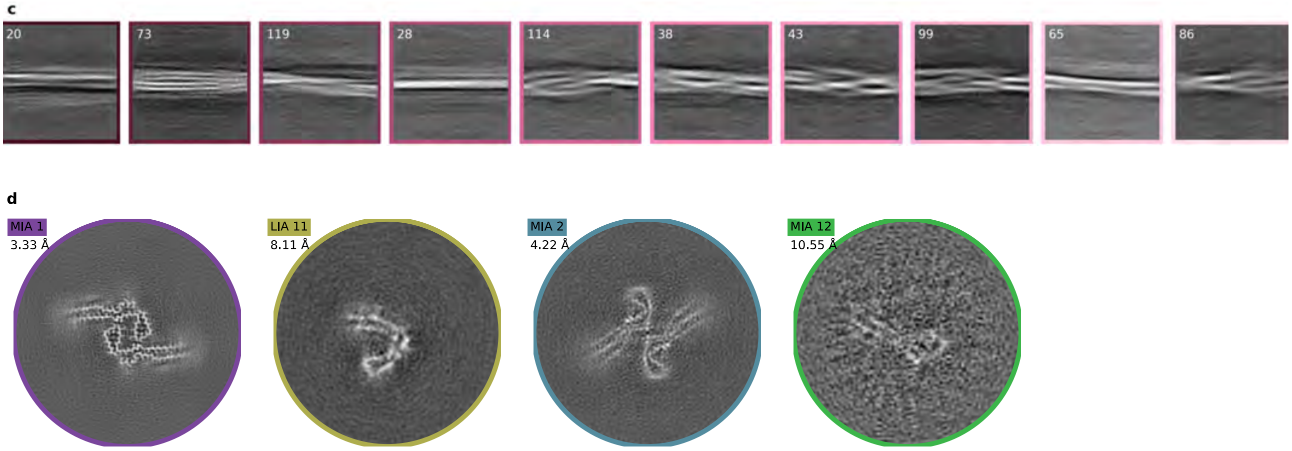

Supplementary Figure 25: Cryo-EM data set summary 25. Data set characteristics are specified in the table on the top right. **a** Hierarchical classification of individual filament segments according to their assigned 2D class average (vertical) and the picked filament ID(horizontal). **b** Pie chart with the relative amounts of different filament types. Grey represents unsolved filaments. Filament types are the same as in Figure 4 of the main text. Different colours represent different time points (120 min in purples; 180 min in blues; 240 min in greens; 300 min in yellows; 360 min in oranges and 720 min in reds). Structures are coloured according to the time point at which they are most abundant, averaged across all replicates. Unique names of filament types are indicated and the same names are used throughout this document. **c** 2D class averages of unsolved filaments. **d** XY-cross-sections, with a projected depth of approximately 4.7 Angstrom or each filament type.

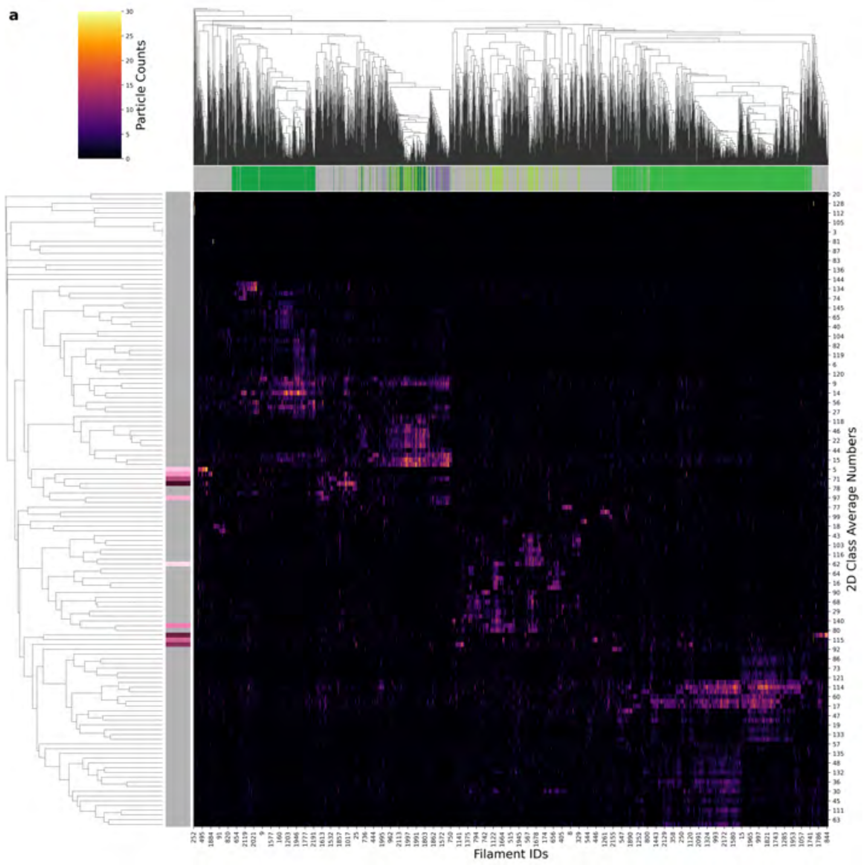

Condition	CTE
Timepoint	160 minutes
Replicate	5
Picking	Manual
Micrographs	2067
Extracted Particles	158080
Pixel Size	0.824 Å
Classified Particles	158080

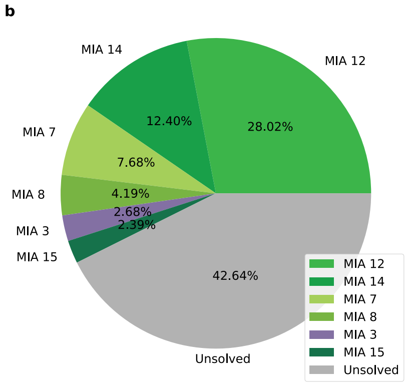

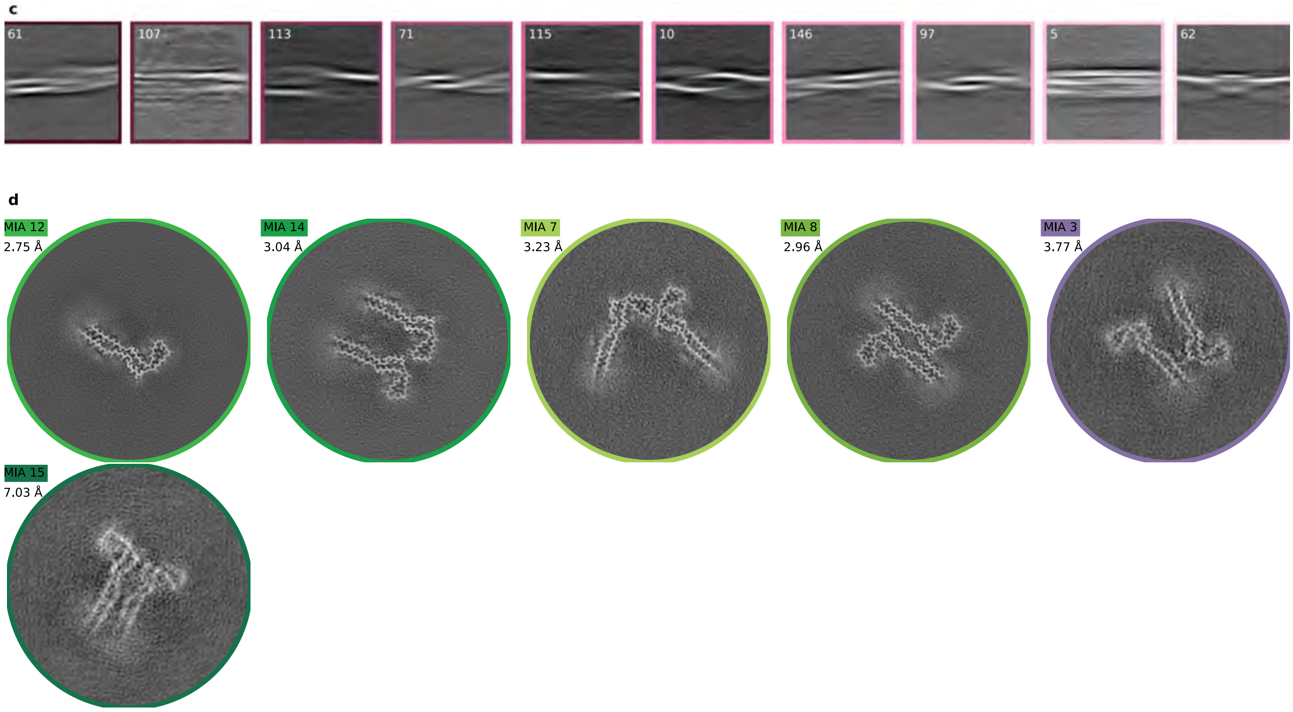

Supplementary Figure 26: Cryo-EM data set summary 26. Data set characteristics are specified in the table on the top right. **a** Hierarchical classification of individual filament segments according to their assigned 2D class average (vertical) and the picked filament ID(horizontal). **b** Pie chart with the relative amounts of different filament types. Grey represents unsolved filaments. Filament types are the same as in Figure 4 of the main text. Different colours represent different time points (120 min in purples; 180 min in blues; 240 min in greens; 300 min in yellows; 360 min in oranges and 720 min in reds). Structures are coloured according to the time point at which they are most abundant, averaged across all replicates. Unique names of filament types are indicated and the same names are used throughout this document. **c** 2D class averages of unsolved filaments. **d** XY-cross-sections, with a projected depth of approximately 4.7 Angstrom or each filament type.

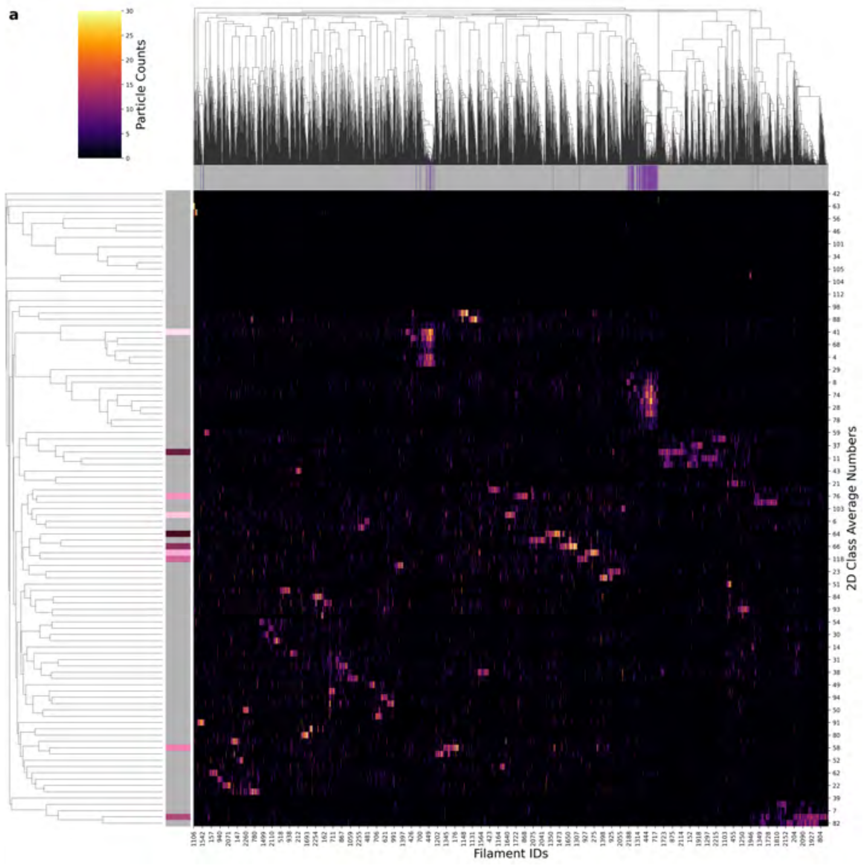

Condition	CTE
Timepoint	160 minutes
Replicate	6
Picking	Manual
Micrographs	5427
Extracted Particles	103098
Pixel Size	0.824 Å
Classified Particles	103098

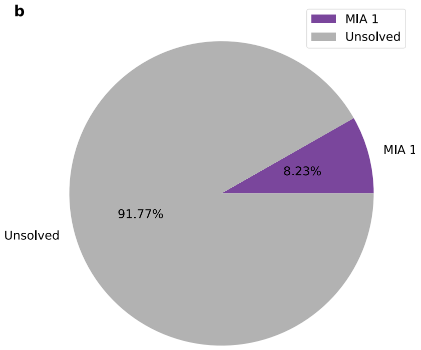

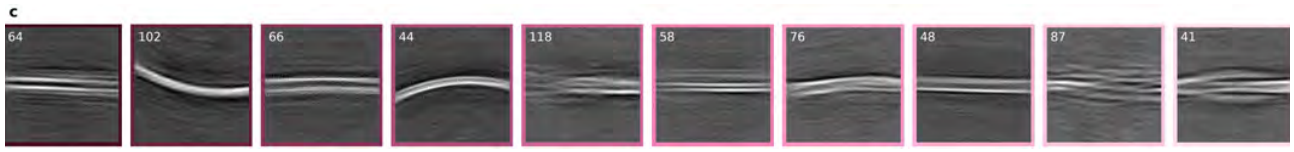

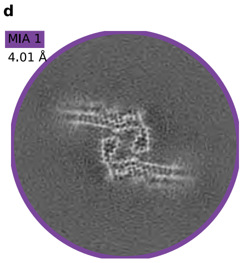

Supplementary Figure 27: Cryo-EM data set summary 27. Data set characteristics are specified in the table on the top right. **a** Hierarchical classification of individual filament segments according to their assigned 2D class average (vertical) and the picked filament ID(horizontal). **b** Pie chart with the relative amounts of different filament types. Grey represents unsolved filaments. Filament types are the same as in Figure 4 of the main text. Different colours represent different time points (120 min in purples; 180 min in blues; 240 min in greens; 300 min in yellows; 360 min in oranges and 720 min in reds). Structures are coloured according to the time point at which they are most abundant, averaged across all replicates. Unique names of filament types are indicated and the same names are used throughout this document. **c** 2D class averages of unsolved filaments. **d** XY-cross-sections, with a projected depth of approximately 4.7 Angstrom or each filament type.

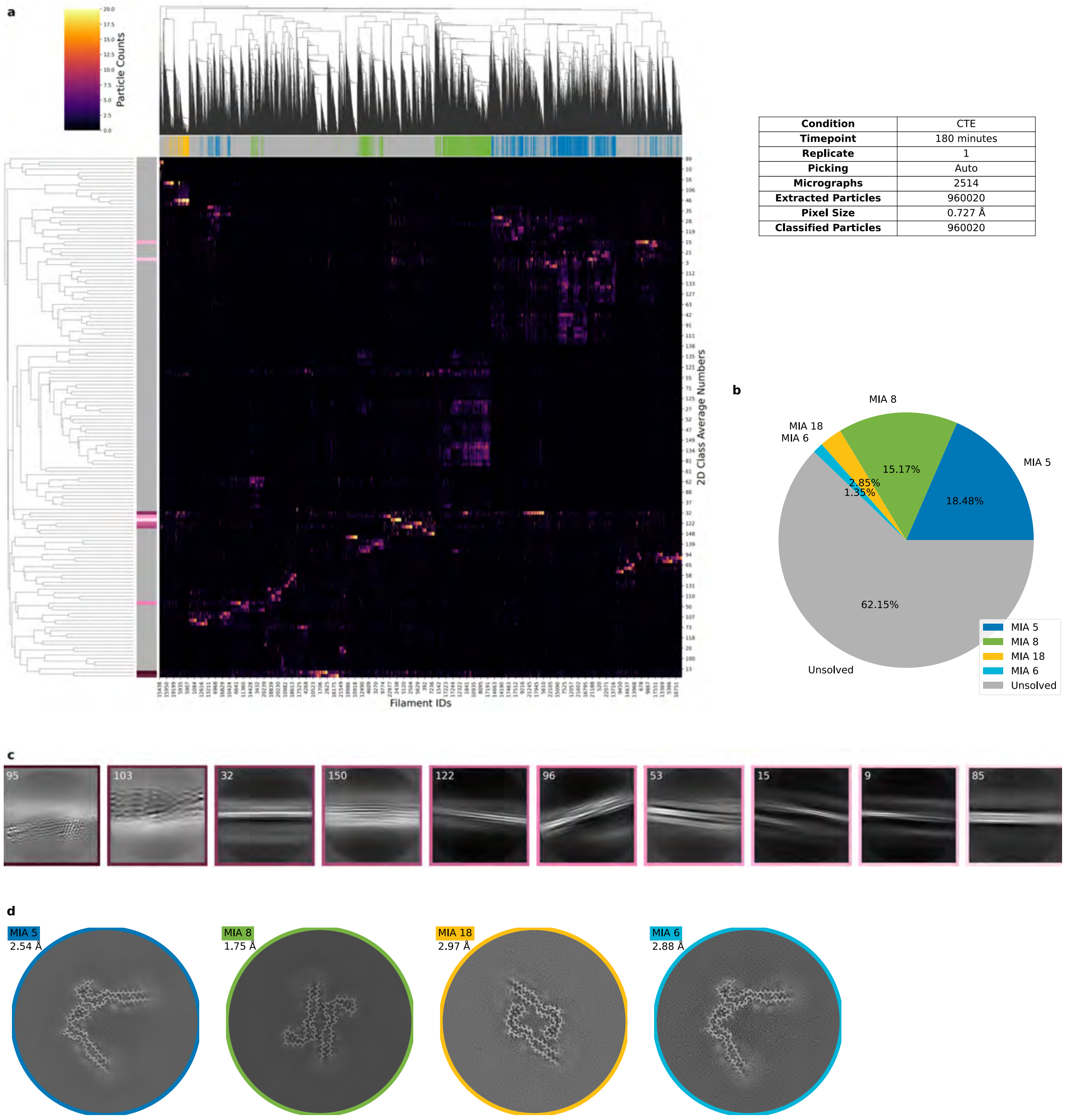

**Condition**	CTE
**Timepoint**	180 minutes
**Replicate**	1
**Picking**	Auto
**Micrographs**	2514
**Extracted Particles**	960020
**Pixel Size**	0.727 Å
**Classified Particles**	960020

Supplementary Figure 28: Cryo-EM data set summary 28. Data set characteristics are specified in the table on the top right. **a** Hierarchical classification of individual filament segments according to their assigned 2D class average (vertical) and the picked filament ID(horizontal). **b** Pie chart with the relative amounts of different filament types. Grey represents unsolved filaments. Filament types are the same as in Figure 4 of the main text. Different colours represent different time points (120 min in purples; 180 min in blues; 240 min in greens; 300 min in yellows; 360 min in oranges and 720 min in reds). Structures are coloured according to the time point at which they are most abundant, averaged across all replicates. Unique names of filament types are indicated and the same names are used throughout this document. **c** 2D class averages of unsolved filaments. **d** XY-cross-sections, with a projected depth of approximately 4.7 Angstrom or each filament type.

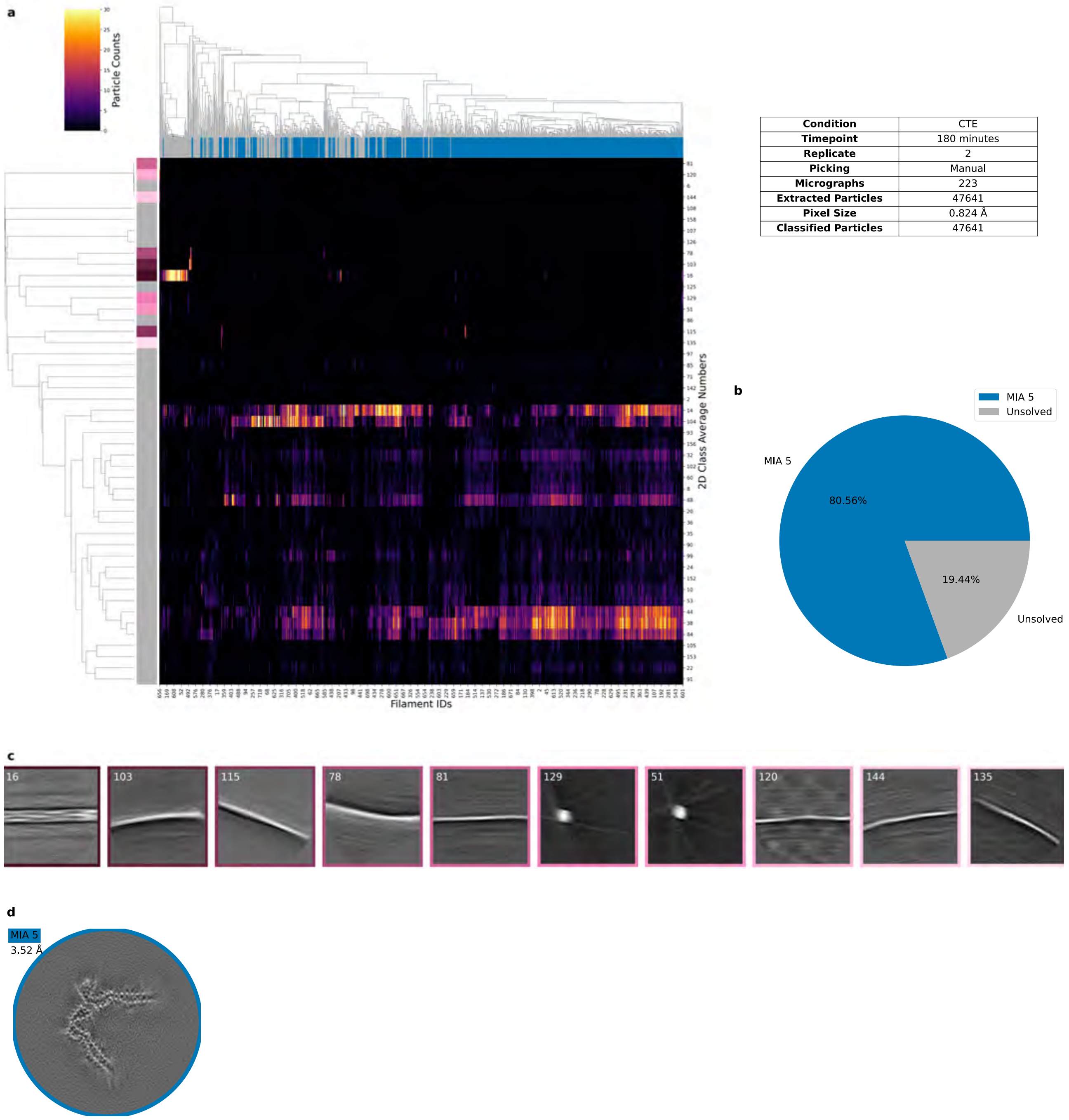

**Condition**	CTE
**Timepoint**	180 minutes
**Replicate**	2
**Picking**	Manual
**Micrographs**	223
**Extracted Particles**	47641
**Pixel Size**	0.824 Å
**Classified Particles**	47641

Supplementary Figure 29: Cryo-EM data set summary 29. Data set characteristics are specified in the table on the top right. **a** Hierarchical classification of individual filament segments according to their assigned 2D class average (vertical) and the picked filament ID(horizontal). **b** Pie chart with the relative amounts of different filament types. Grey represents unsolved filaments. Filament types are the same as in Figure 4 of the main text. Different colours represent different time points (120 min in purples; 180 min in blues; 240 min in greens; 300 min in yellows; 360 min in oranges and 720 min in reds). Structures are coloured according to the time point at which they are most abundant, averaged across all replicates. Unique names of filament types are indicated and the same names are used throughout this document. **c** 2D class averages of unsolved filaments. **d** XY-cross-sections, with a projected depth of approximately 4.7 Angstrom or each filament type.

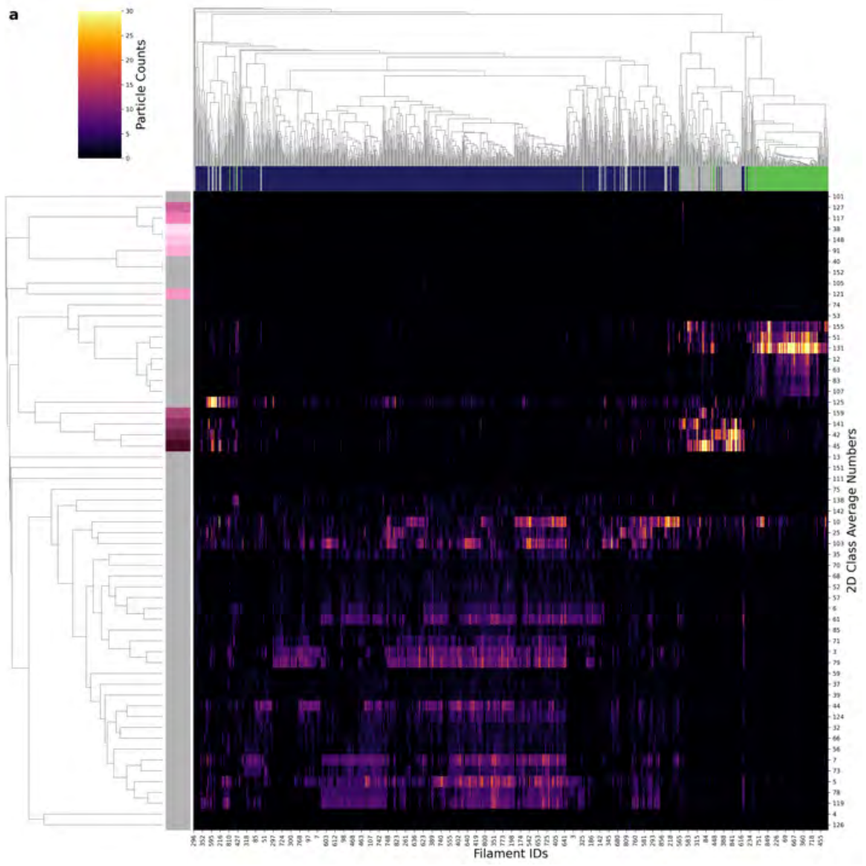

**Condition**	CTE
**Timepoint**	180 minutes
**Replicate**	4
**Picking**	Manual
**Micrographs**	1782
**Extracted Particles**	58123
**Pixel Size**	0.824 Å
**Classified Particles**	58123

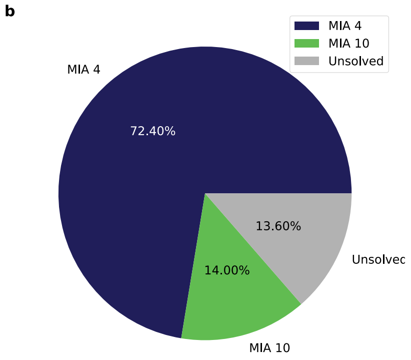

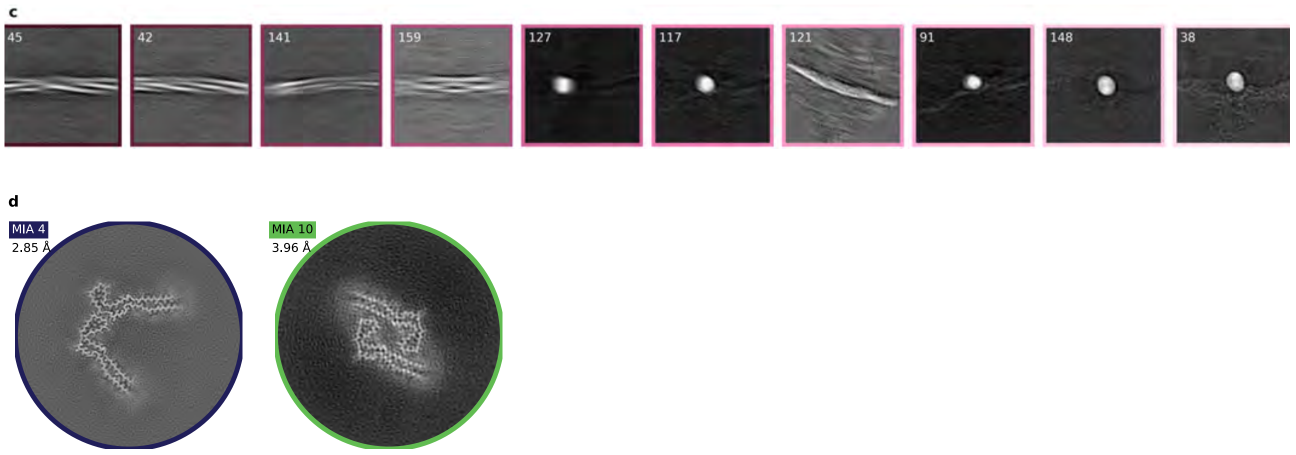

Supplementary Figure 30: Cryo-EM data set summary 30. Data set characteristics are specified in the table on the top right. **a** Hierarchical classification of individual filament segments according to their assigned 2D class average (vertical) and the picked filament ID(horizontal). **b** Pie chart with the relative amounts of different filament types. Grey represents unsolved filaments. Filament types are the same as in Figure 4 of the main text. Different colours represent different time points (120 min in purples; 180 min in blues; 240 min in greens; 300 min in yellows; 360 min in oranges and 720 min in reds). Structures are coloured according to the time point at which they are most abundant, averaged across all replicates. Unique names of filament types are indicated and the same names are used throughout this document. **c** 2D class averages of unsolved filaments. **d** XY-cross-sections, with a projected depth of approximately 4.7 Angstrom or each filament type.

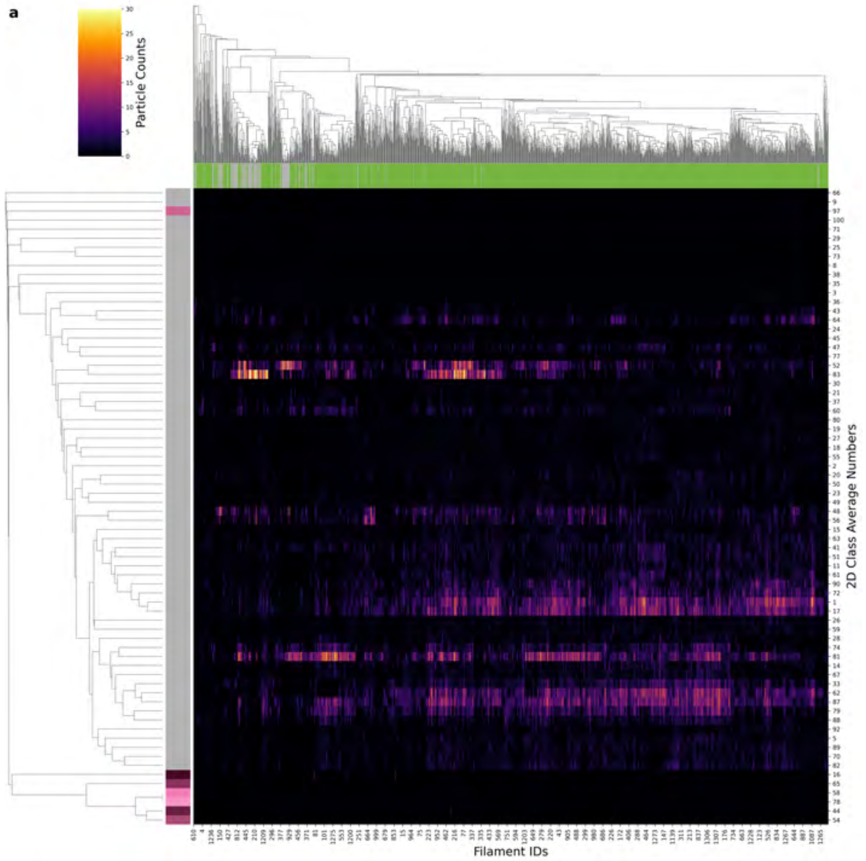

Condition	CTE
Timepoint	180 minutes
Replicate	5
Picking	Manual
Micrographs	1385
Extracted Particles	99429
Pixel Size	0.824 Å
Classified Particles	99429

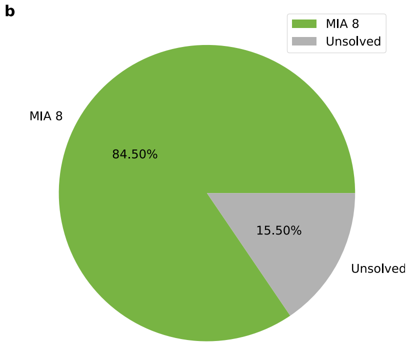

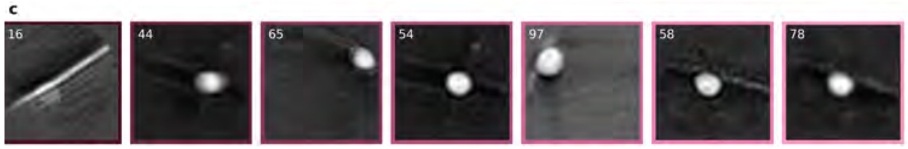

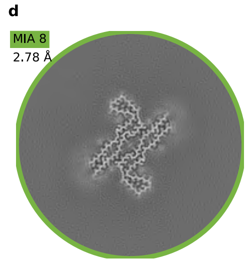

Supplementary Figure 31: Cryo-EM data set summary 31. Data set characteristics are specified in the table on the top right. **a** Hierarchical classification of individual filament segments according to their assigned 2D class average (vertical) and the picked filament ID(horizontal). **b** Pie chart with the relative amounts of different filament types. Grey represents unsolved filaments. Filament types are the same as in Figure 4 of the main text. Different colours represent different time points (120 min in purples; 180 min in blues; 240 min in greens; 300 min in yellows; 360 min in oranges and 720 min in reds). Structures are coloured according to the time point at which they are most abundant, averaged across all replicates. Unique names of filament types are indicated and the same names are used throughout this document. **c** 2D class averages of unsolved filaments. **d** XY-cross-sections, with a projected depth of approximately 4.7 Angstrom or each filament type.

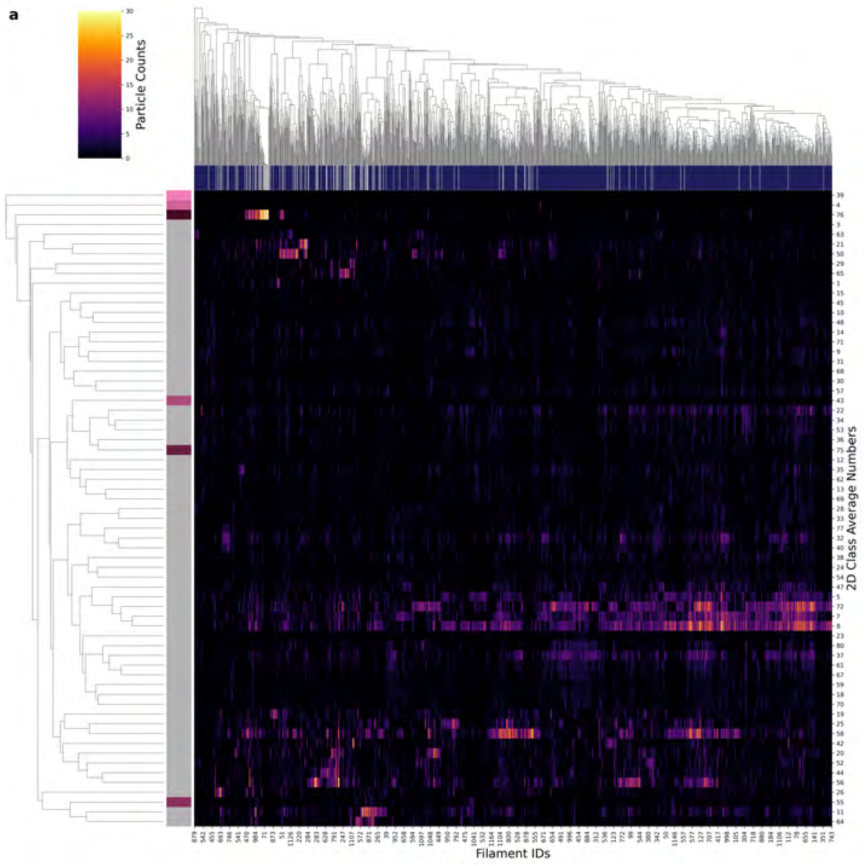

Condition	CTE
Timepoint	180 minutes
Replicate	6
Picking	Manual
Micrographs	601
Extracted Particles	71046
Pixel Size	0.824 Å
Classified Particles	71046

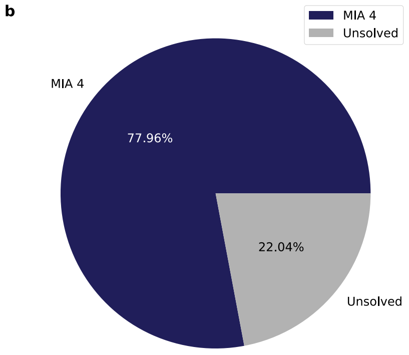

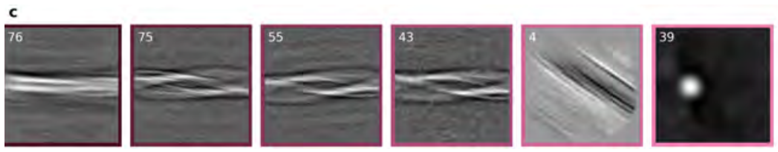

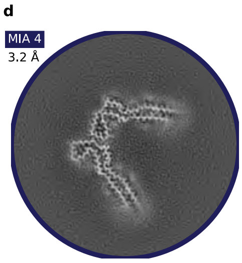

Supplementary Figure 32: Cryo-EM data set summary 32. Data set characteristics are specified in the table on the top right. **a** Hierarchical classification of individual filament segments according to their assigned 2D class average (vertical) and the picked filament ID(horizontal). **b** Pie chart with the relative amounts of different filament types. Grey represents unsolved filaments. Filament types are the same as in Figure 4 of the main text. Different colours represent different time points (120 min in purples; 180 min in blues; 240 min in greens; 300 min in yellows; 360 min in oranges and 720 min in reds). Structures are coloured according to the time point at which they are most abundant, averaged across all replicates. Unique names of filament types are indicated and the same names are used throughout this document. **c** 2D class averages of unsolved filaments. **d** XY-cross-sections, with a projected depth of approximately 4.7 Angstrom or each filament type.

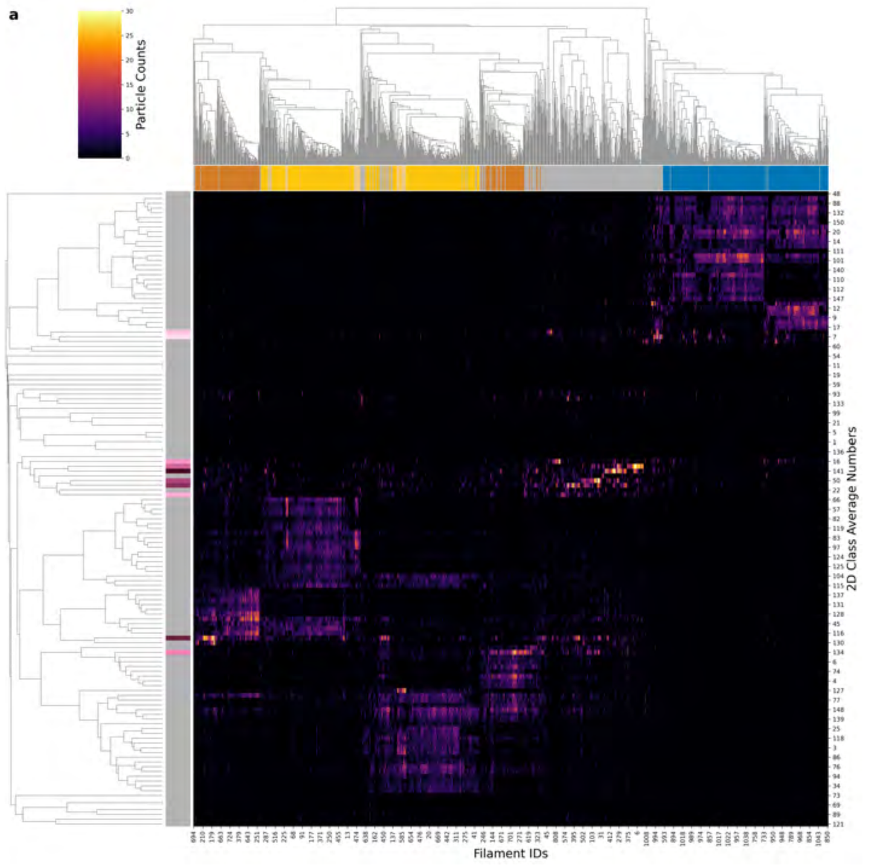

Condition	CTE
Timepoint	240 minutes
Replicate	1
Picking	Manual
Micrographs	3902
Extracted Particles	95858
Pixel Size	0.727 Å
Classified Particles	95858

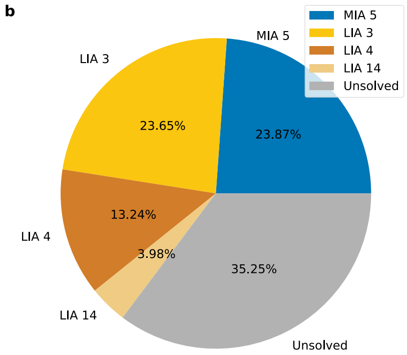

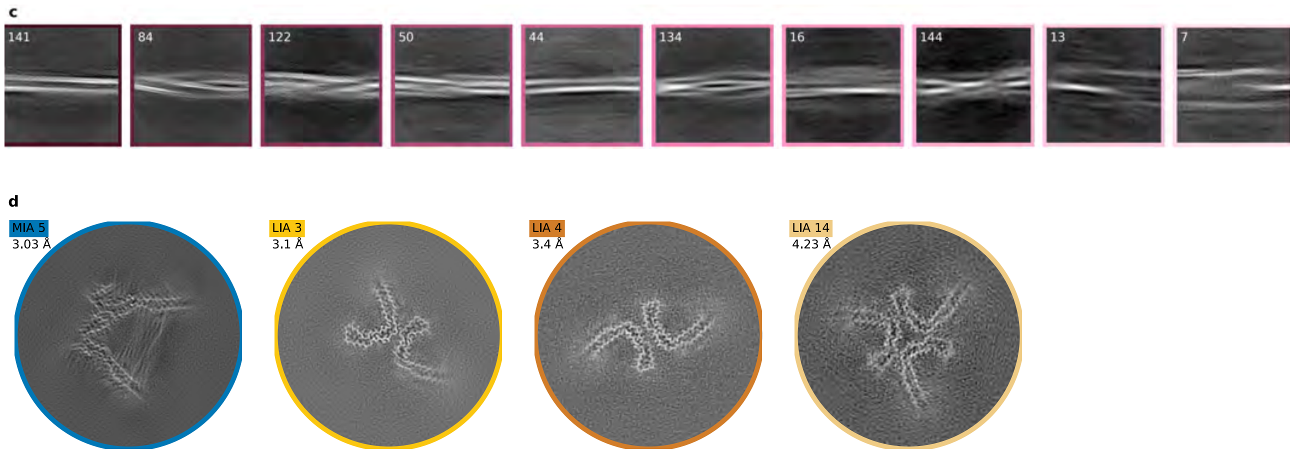

Supplementary Figure 33: Cryo-EM data set summary 33. Data set characteristics are specified in the table on the top right. **a** Hierarchical classification of individual filament segments according to their assigned 2D class average (vertical) and the picked filament ID(horizontal). **b** Pie chart with the relative amounts of different filament types. Grey represents unsolved filaments. Filament types are the same as in Figure 4 of the main text. Different colours represent different time points (120 min in purples; 180 min in blues; 240 min in greens; 300 min in yellows; 360 min in oranges and 720 min in reds). Structures are coloured according to the time point at which they are most abundant, averaged across all replicates. Unique names of filament types are indicated and the same names are used throughout this document. **c** 2D class averages of unsolved filaments. **d** XY-cross-sections, with a projected depth of approximately 4.7 Angstrom or each filament type.

a

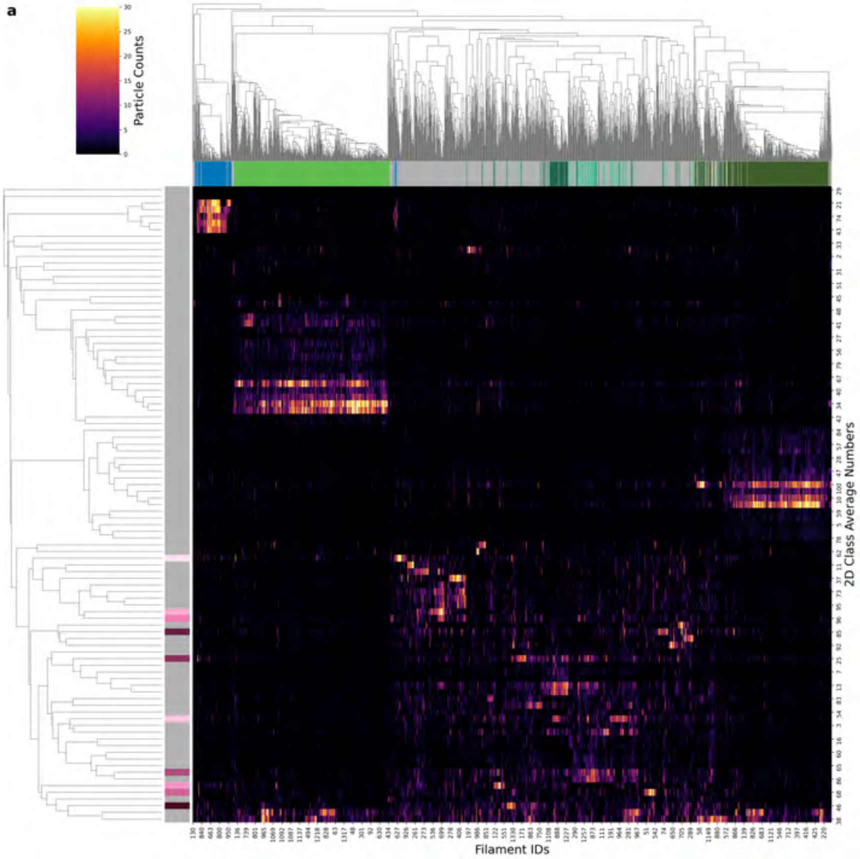

Condition	CTE
**Timepoint**	240 minutes
**Replicate**	2
**Picking**	Manual
**Micrographs**	942
**Extracted Particles**	123830
**Pixel Size**	0.824 Å
**Classified Particles**	123830

b

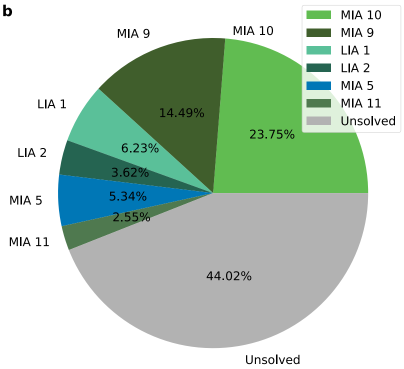

c

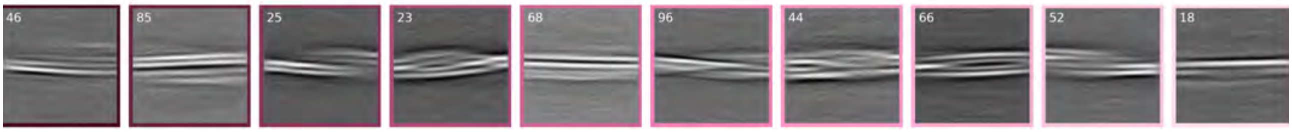

d

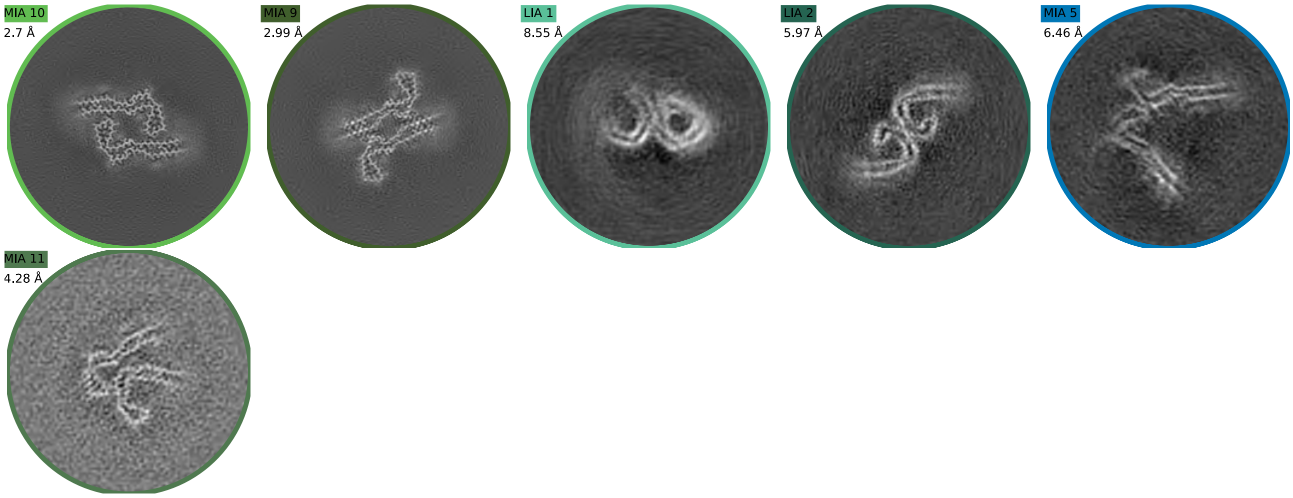

Supplementary Figure 34: Cryo-EM data set summary 34. Data set characteristics are specified in the table on the top right. **a** Hierarchical classification of individual filament segments according to their assigned 2D class average (vertical) and the picked filament ID(horizontal). **b** Pie chart with the relative amounts of different filament types. Grey represents unsolved filaments. Filament types are the same as in Figure 4 of the main text. Different colours represent different time points (120 min in purples; 180 min in blues; 240 min in greens; 300 min in yellows; 360 min in oranges and 720 min in reds). Structures are coloured according to the time point at which they are most abundant, averaged across all replicates. Unique names of filament types are indicated and the same names are used throughout this document. **c** 2D class averages of unsolved filaments. **d** XY-cross-sections, with a projected depth of approximately 4.7 Angstrom or each filament type.

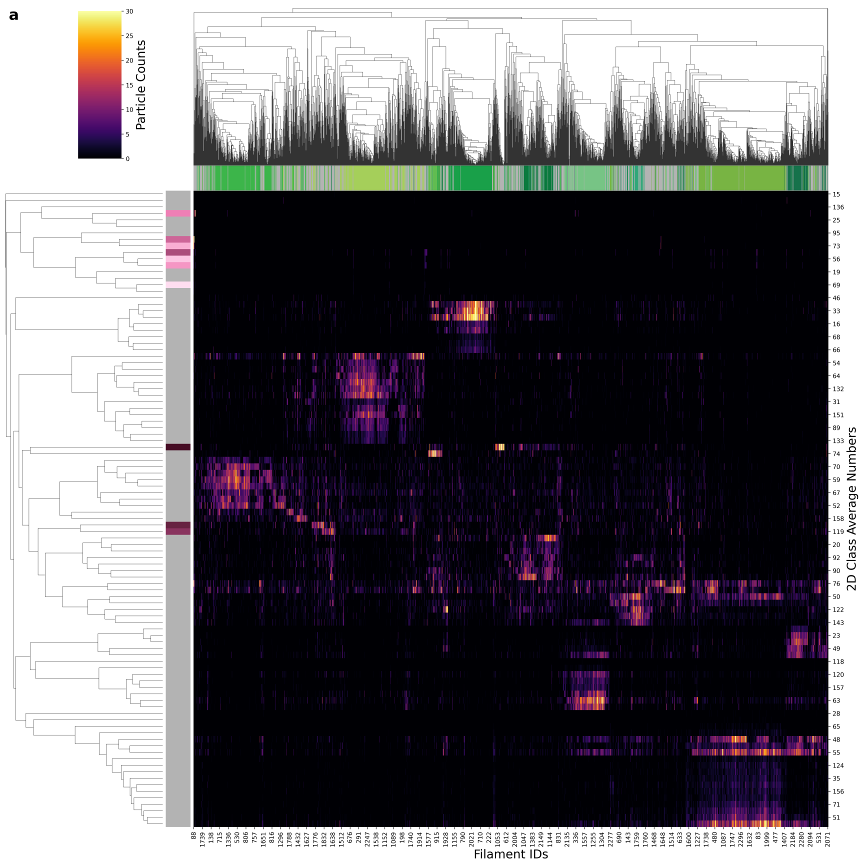

Condition	CTE
**Timepoint**	240 minutes
**Replicate**	3
**Picking**	Manual
**Micrographs**	967
**Extracted Particles**	188025
**Pixel Size**	0.824 Å
**Classified Particles**	188025

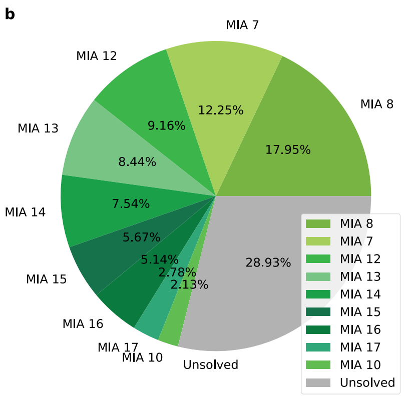

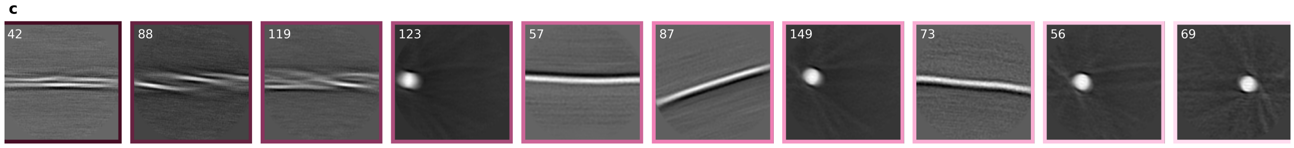

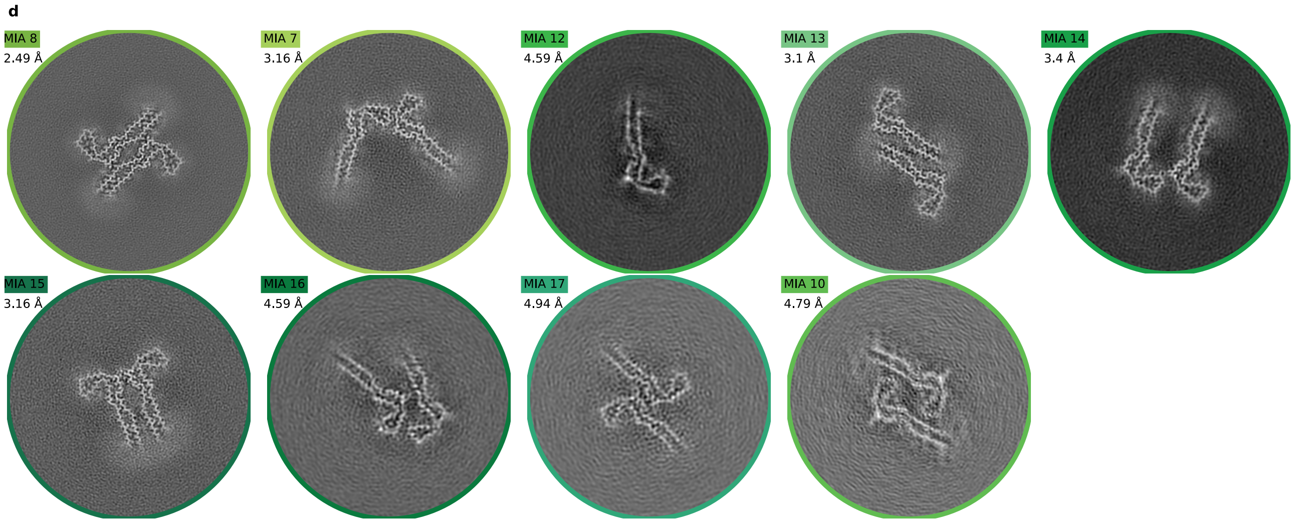

Supplementary Figure 35: Cryo-EM data set summary 35. Data set characteristics are specified in the table on the top right. **a** Hierarchical classification of individual filament segments according to their assigned 2D class average (vertical) and the picked filament ID(horizontal). **b** Pie chart with the relative amounts of different filament types. Grey represents unsolved filaments. Filament types are the same as in Figure 4 of the main text. Different colours represent different time points (120 min in purples; 180 min in blues; 240 min in greens; 300 min in yellows; 360 min in oranges and 720 min in reds). Structures are coloured according to the time point at which they are most abundant, averaged across all replicates. Unique names of filament types are indicated and the same names are used throughout this document. **c** 2D class averages of unsolved filaments. **d** XY-cross-sections, with a projected depth of approximately 4.7 Angstrom or each filament type.

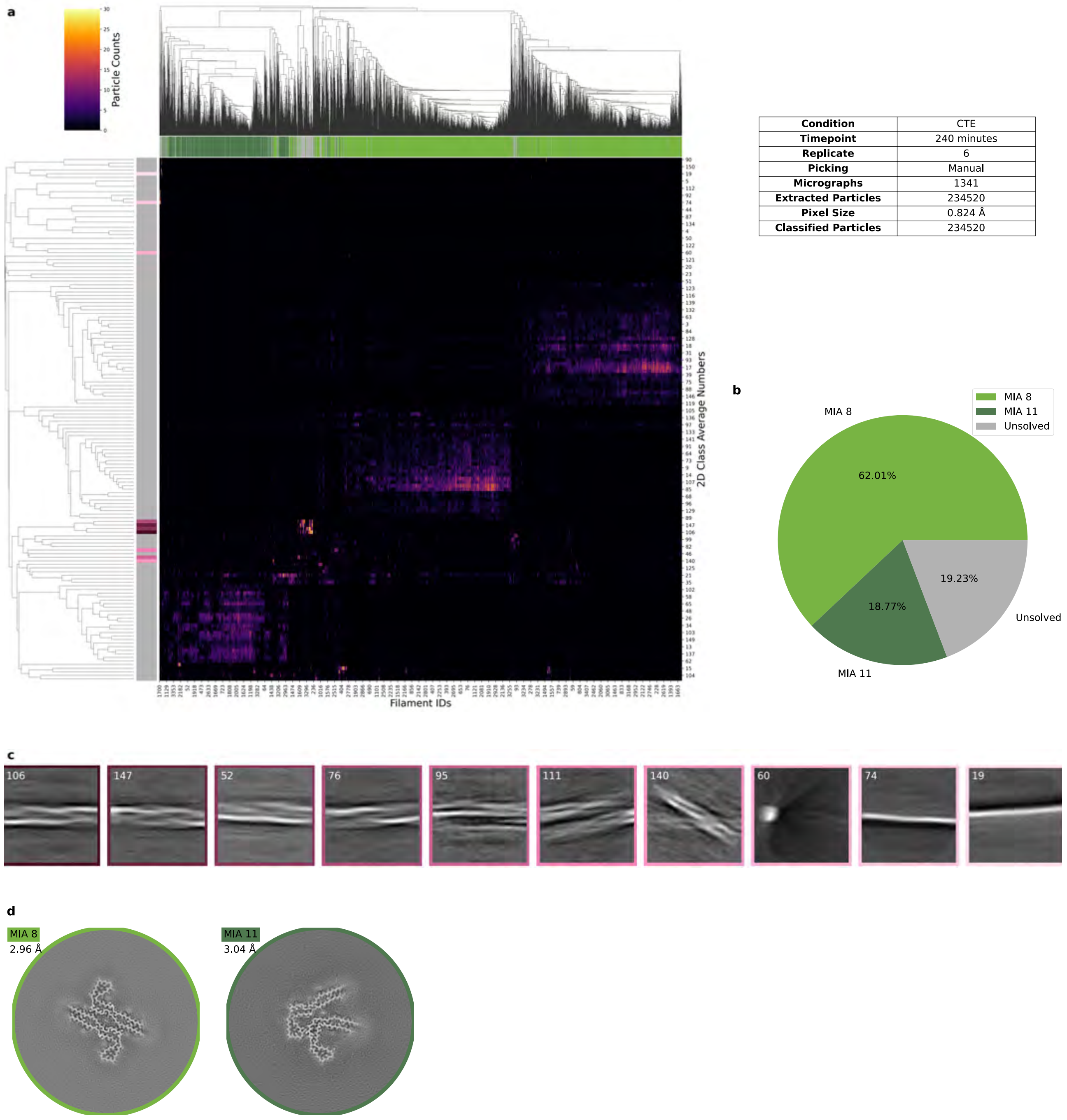

**Condition**	CTE
**Timepoint**	240 minutes
**Replicate**	6
**Picking**	Manual
**Micrographs**	1341
**Extracted Particles**	234520
**Pixel Size**	0.824 Å
**Classified Particles**	234520

Supplementary Figure 36: Cryo-EM data set summary 36. Data set characteristics are specified in the table on the top right. **a** Hierarchical classification of individual filament segments according to their assigned 2D class average (vertical) and the picked filament ID(horizontal). **b** Pie chart with the relative amounts of different filament types. Grey represents unsolved filaments. Filament types are the same as in Figure 4 of the main text. Different colours represent different time points (120 min in purples; 180 min in blues; 240 min in greens; 300 min in yellows; 360 min in oranges and 720 min in reds). Structures are coloured according to the time point at which they are most abundant, averaged across all replicates. Unique names of filament types are indicated and the same names are used throughout this document. **c** 2D class averages of unsolved filaments. **d** XY-cross-sections, with a projected depth of approximately 4.7 Angstrom or each filament type.

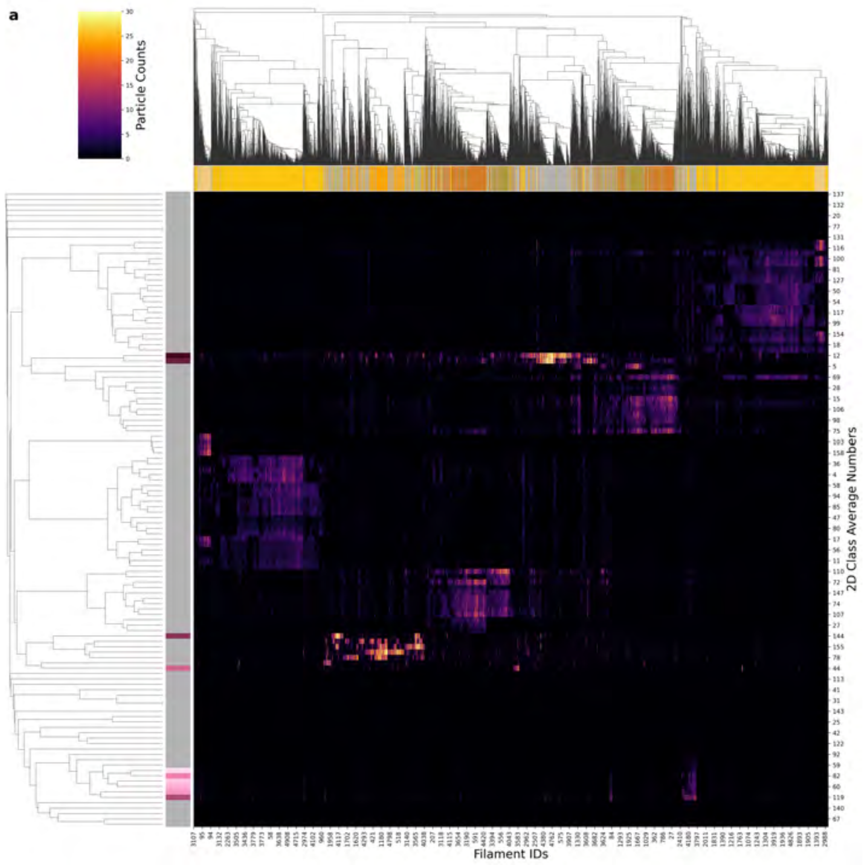

Condition	CTE
**Timepoint**	300 minutes
**Replicate**	1
**Picking**	Manual
**Micrographs**	3141
**Extracted Particles**	317057
**Pixel Size**	0.727 Å
**Classified Particles**	317057

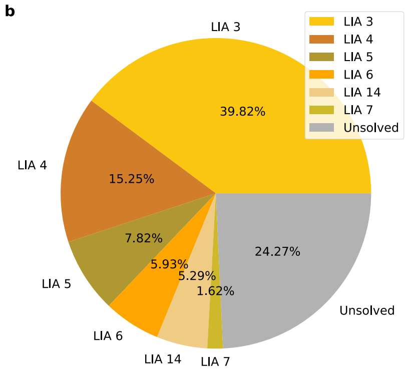

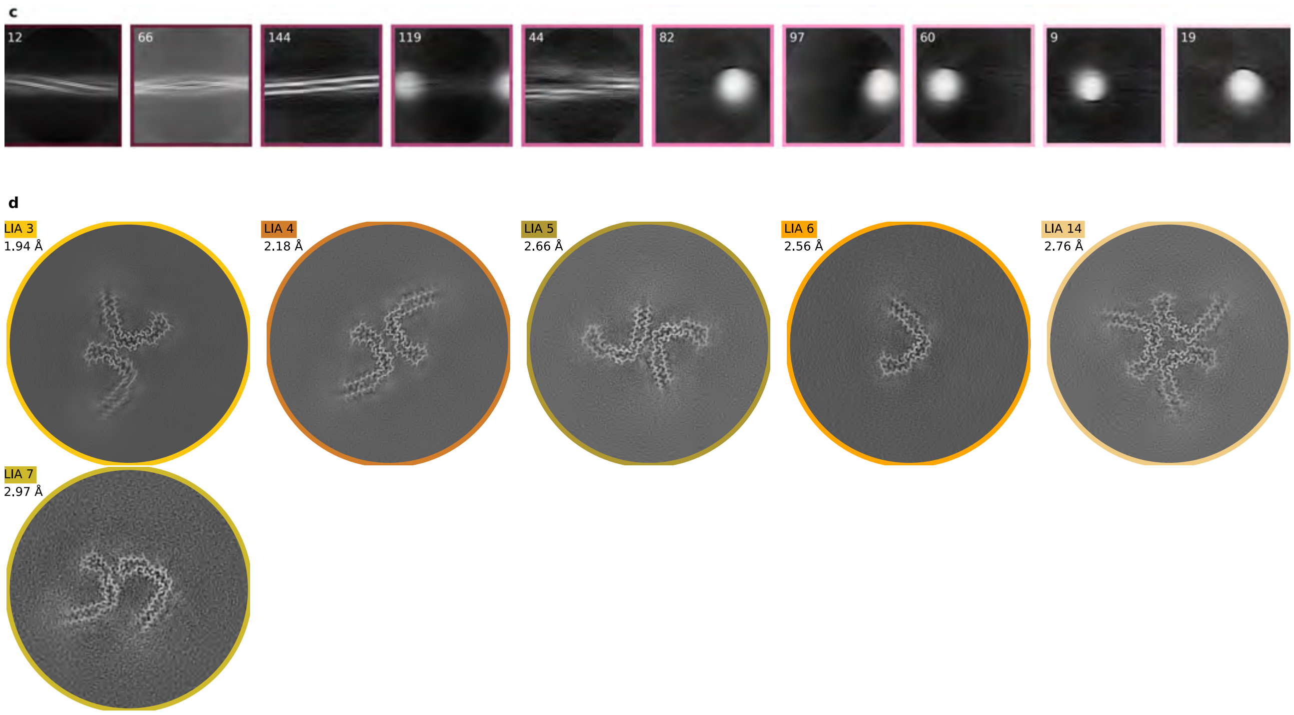

Supplementary Figure 37: Cryo-EM data set summary 37. Data set characteristics are specified in the table on the top right. **a** Hierarchical classification of individual filament segments according to their assigned 2D class average (vertical) and the picked filament ID(horizontal). **b** Pie chart with the relative amounts of different filament types. Grey represents unsolved filaments. Filament types are the same as in Figure 4 of the main text. Different colours represent different time points (120 min in purples; 180 min in blues; 240 min in greens; 300 min in yellows; 360 min in oranges and 720 min in reds). Structures are coloured according to the time point at which they are most abundant, averaged across all replicates. Unique names of filament types are indicated and the same names are used throughout this document. **c** 2D class averages of unsolved filaments. **d** XY-cross-sections, with a projected depth of approximately 4.7 Angstrom or each filament type.

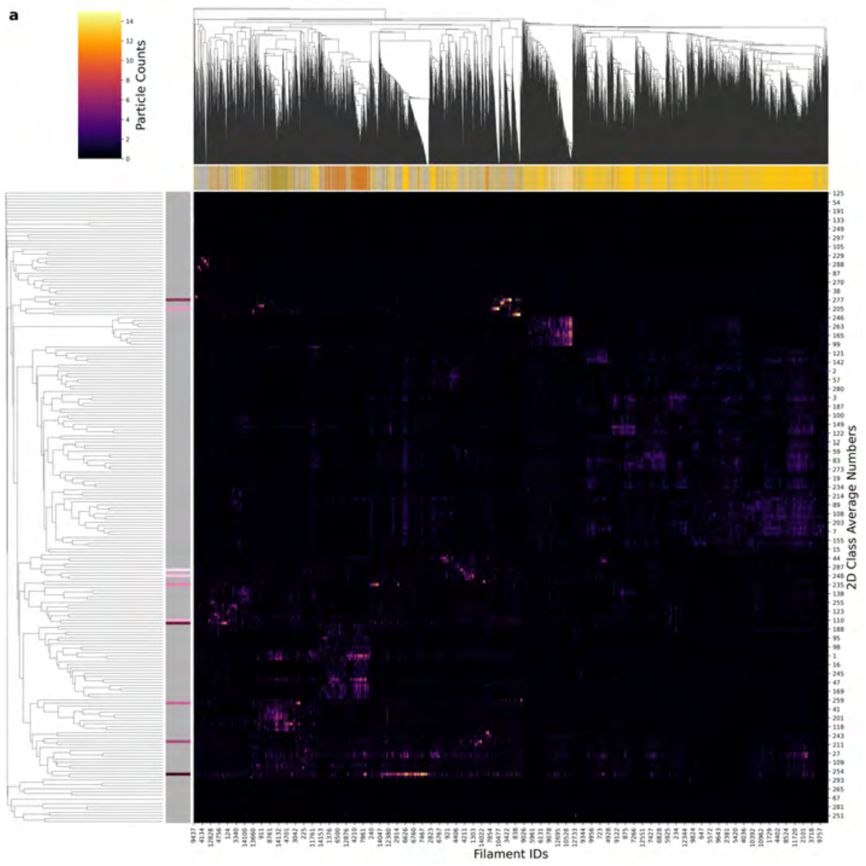

Condition	CTE
Timepoint	300 minutes
Replicate	2
Picking	Auto
Micrographs	2770
Extracted Particles	2054796
Pixel Size	0.727 Å
Classified Particles	540669

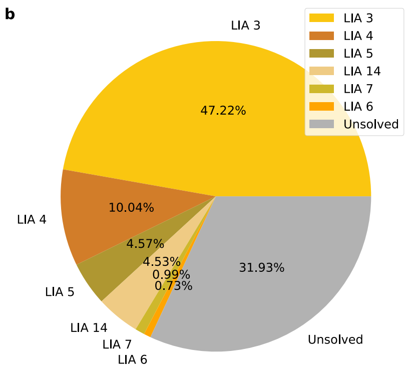

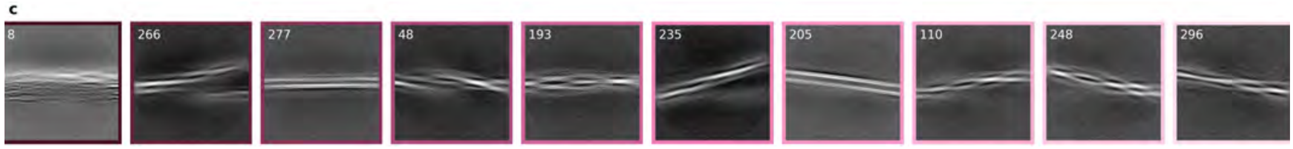

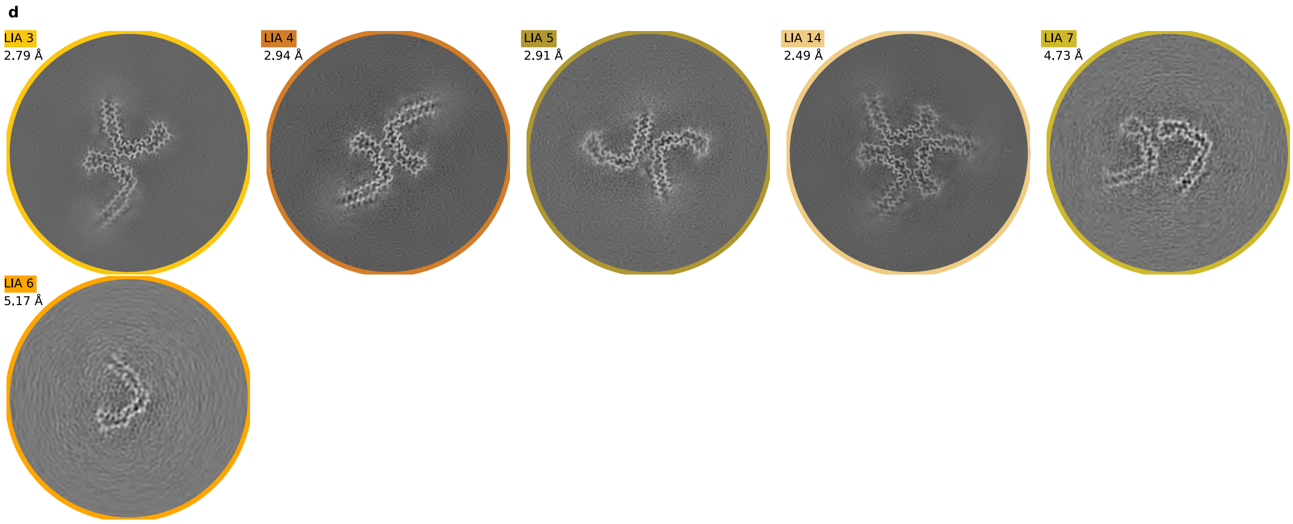

Supplementary Figure 38: Cryo-EM data set summary 38. Data set characteristics are specified in the table on the top right. **a** Hierarchical classification of individual filament segments according to their assigned 2D class average (vertical) and the picked filament ID(horizontal). **b** Pie chart with the relative amounts of different filament types. Grey represents unsolved filaments. Filament types are the same as in Figure 4 of the main text. Different colours represent different time points (120 min in purples; 180 min in blues; 240 min in greens; 300 min in yellows; 360 min in oranges and 720 min in reds). Structures are coloured according to the time point at which they are most abundant, averaged across all replicates. Unique names of filament types are indicated and the same names are used throughout this document. **c** 2D class averages of unsolved filaments. **d** XY-cross-sections, with a projected depth of approximately 4.7 Angstrom or each filament type.

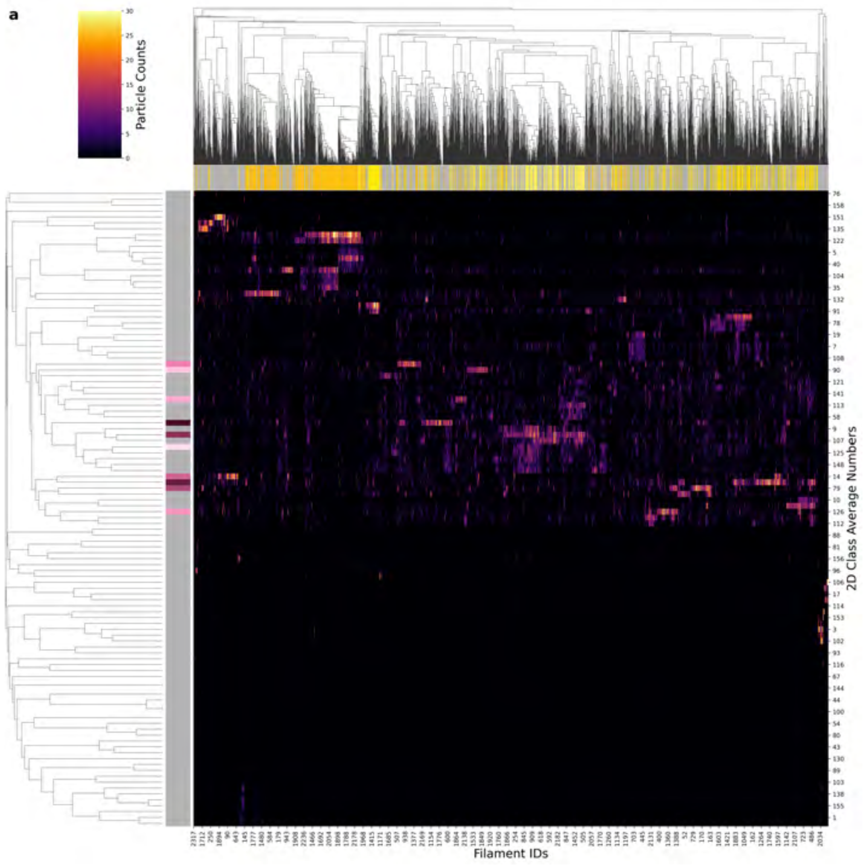

**Condition**	CTE
**Timepoint**	300 minutes
**Replicate**	3
**Picking**	Manual
**Micrographs**	711
**Extracted Particles**	134013
**Pixel Size**	0.824 Å
**Classified Particles**	134013

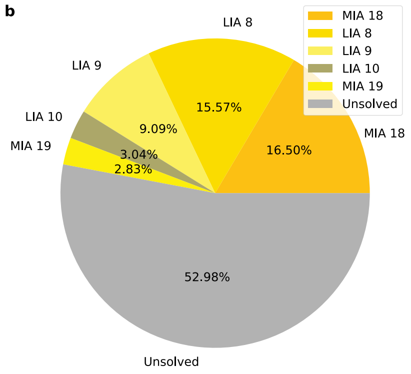

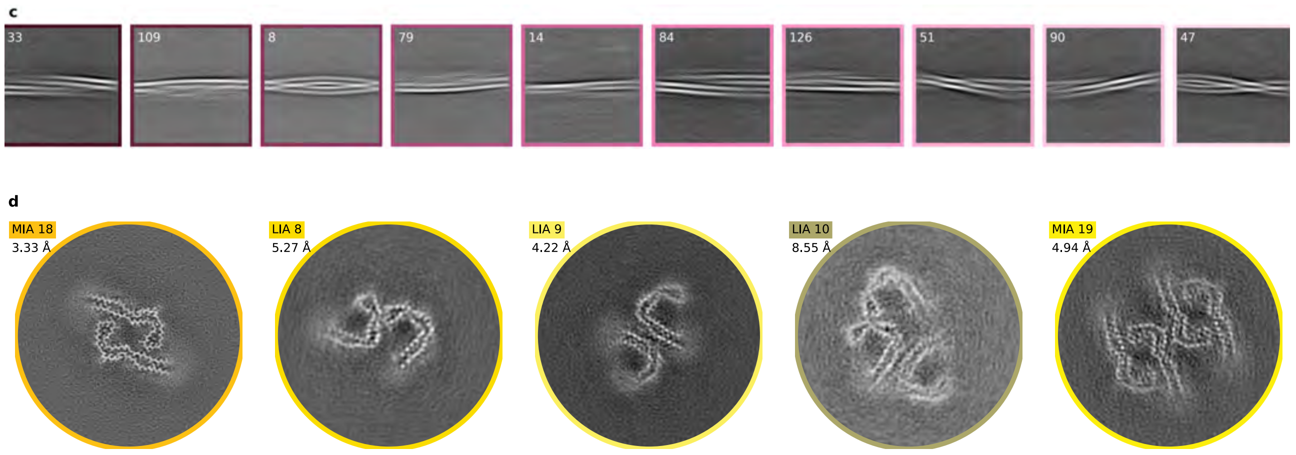

Supplementary Figure 39: Cryo-EM data set summary 39. Data set characteristics are specified in the table on the top right. **a** Hierarchical classification of individual filament segments according to their assigned 2D class average (vertical) and the picked filament ID(horizontal). **b** Pie chart with the relative amounts of different filament types. Grey represents unsolved filaments. Filament types are the same as in Figure 4 of the main text. Different colours represent different time points (120 min in purples; 180 min in blues; 240 min in greens; 300 min in yellows; 360 min in oranges and 720 min in reds). Structures are coloured according to the time point at which they are most abundant, averaged across all replicates. Unique names of filament types are indicated and the same names are used throughout this document. **c** 2D class averages of unsolved filaments. **d** XY-cross-sections, with a projected depth of approximately 4.7 Angstrom or each filament type.

Condition	CTE
Timepoint	300 minutes
Replicate	6
Picking	Manual
Micrographs	1577
Extracted Particles	181708
Pixel Size	0.824 Å
Classified Particles	181708

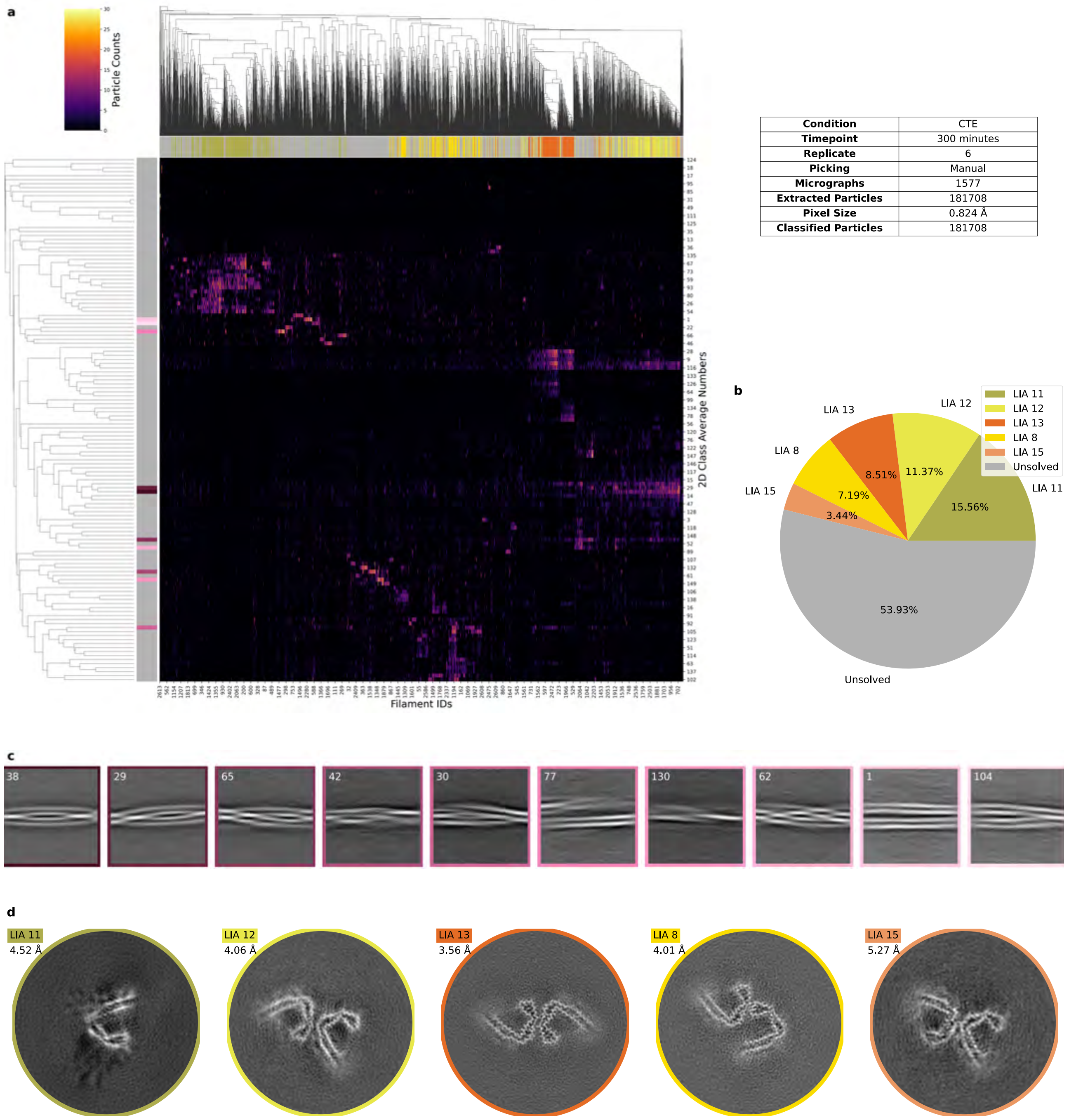

Supplementary Figure 40: Cryo-EM data set summary 40. Data set characteristics are specified in the table on the top right. **a** Hierarchical classification of individual filament segments according to their assigned 2D class average (vertical) and the picked filament ID(horizontal). **b** Pie chart with the relative amounts of different filament types. Grey represents unsolved filaments. Filament types are the same as in Figure 4 of the main text. Different colours represent different time points (120 min in purples; 180 min in blues; 240 min in greens; 300 min in yellows; 360 min in oranges and 720 min in reds). Structures are coloured according to the time point at which they are most abundant, averaged across all replicates. Unique names of filament types are indicated and the same names are used throughout this document. **c** 2D class averages of unsolved filaments. **d** XY-cross-sections, with a projected depth of approximately 4.7 Angstrom or each filament type.

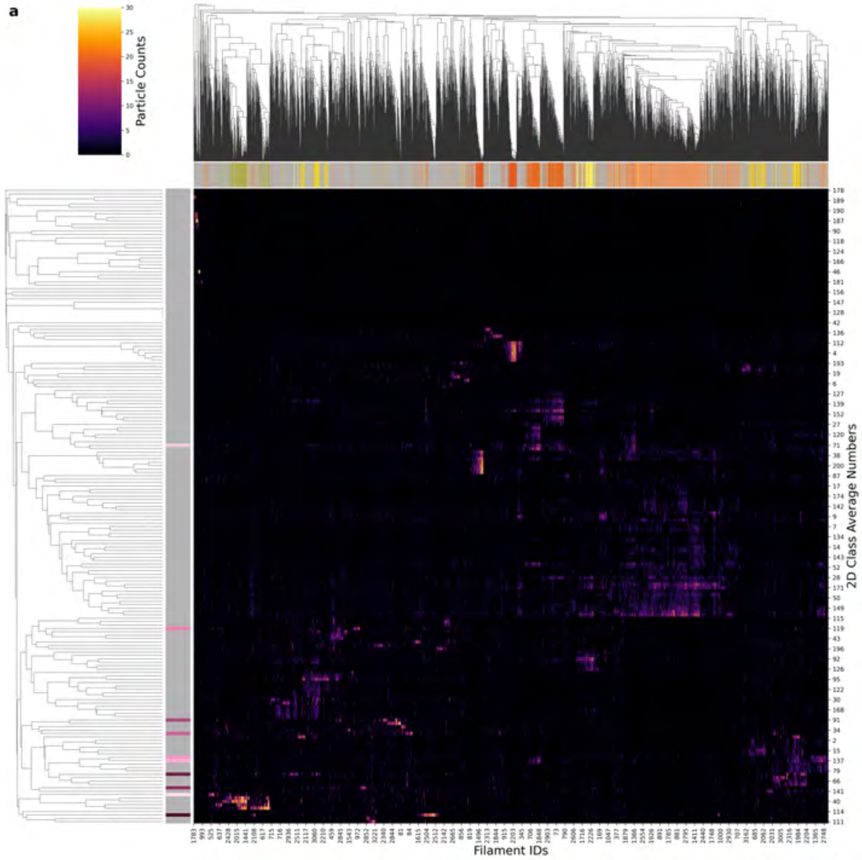

Condition	CTE
Timepoint	360 minutes
Replicate	1
Picking	Manual
Micrographs	233495
Extracted Particles	2943
Pixel Size	0.824 Å
Classified Particles	233495

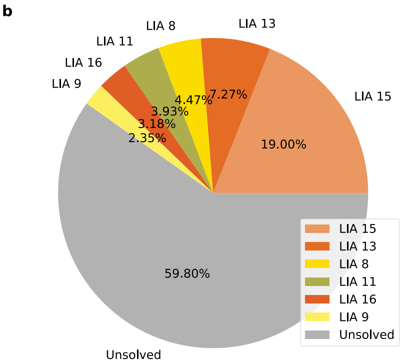

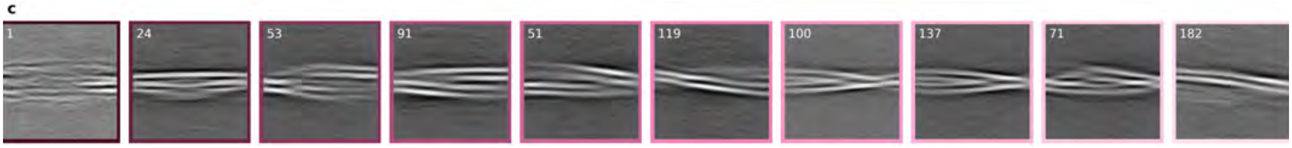

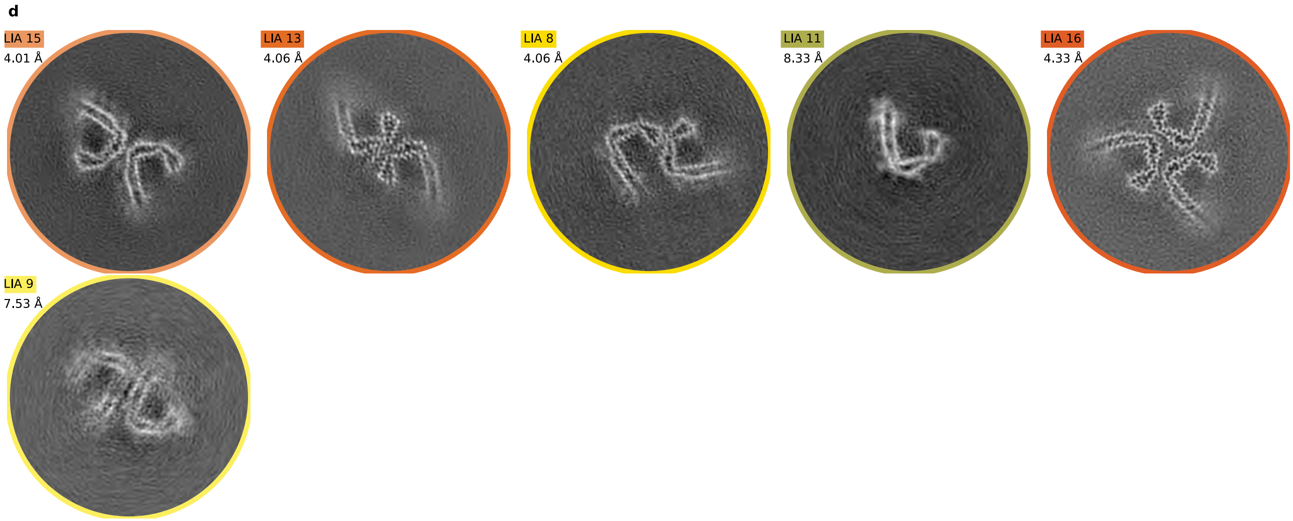

Supplementary Figure 41: Cryo-EM data set summary 41. Data set characteristics are specified in the table on the top right. **a** Hierarchical classification of individual filament segments according to their assigned 2D class average (vertical) and the picked filament ID(horizontal). **b** Pie chart with the relative amounts of different filament types. Grey represents unsolved filaments. Filament types are the same as in Figure 4 of the main text. Different colours represent different time points (120 min in purples; 180 min in blues; 240 min in greens; 300 min in yellows; 360 min in oranges and 720 min in reds). Structures are coloured according to the time point at which they are most abundant, averaged across all replicates. Unique names of filament types are indicated and the same names are used throughout this document. **c** 2D class averages of unsolved filaments. **d** XY-cross-sections, with a projected depth of approximately 4.7 Angstrom or each filament type.

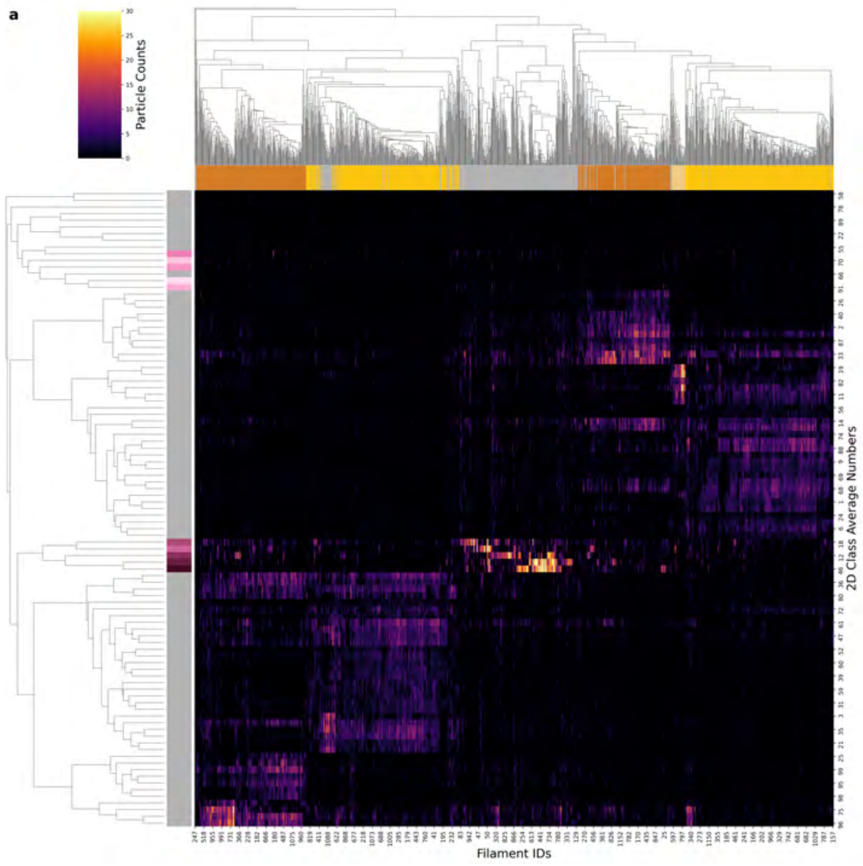

Condition	CTE
Timepoint	360 minutes
Replicate	4
Picking	Manual
Micrographs	2183
Extracted Particles	104881
Pixel Size	0.727 Å
Classified Particles	104881

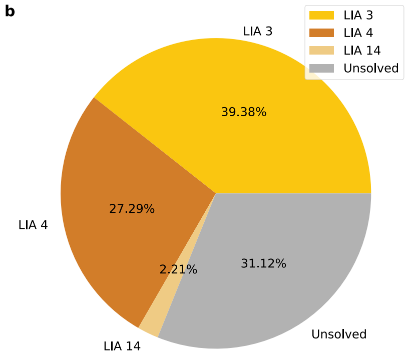

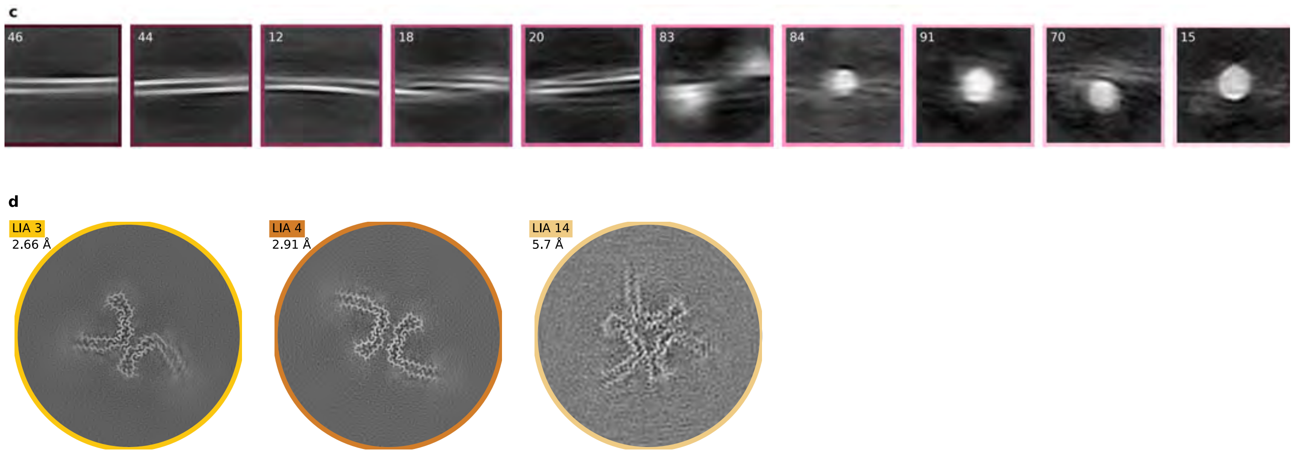

Supplementary Figure 42: Cryo-EM data set summary 42. Data set characteristics are specified in the table on the top right. **a** Hierarchical classification of individual filament segments according to their assigned 2D class average (vertical) and the picked filament ID(horizontal). **b** Pie chart with the relative amounts of different filament types. Grey represents unsolved filaments. Filament types are the same as in Figure 4 of the main text. Different colours represent different time points (120 min in purples; 180 min in blues; 240 min in greens; 300 min in yellows; 360 min in oranges and 720 min in reds). Structures are coloured according to the time point at which they are most abundant, averaged across all replicates. Unique names of filament types are indicated and the same names are used throughout this document. **c** 2D class averages of unsolved filaments. **d** XY-cross-sections, with a projected depth of approximately 4.7 Angstrom or each filament type.

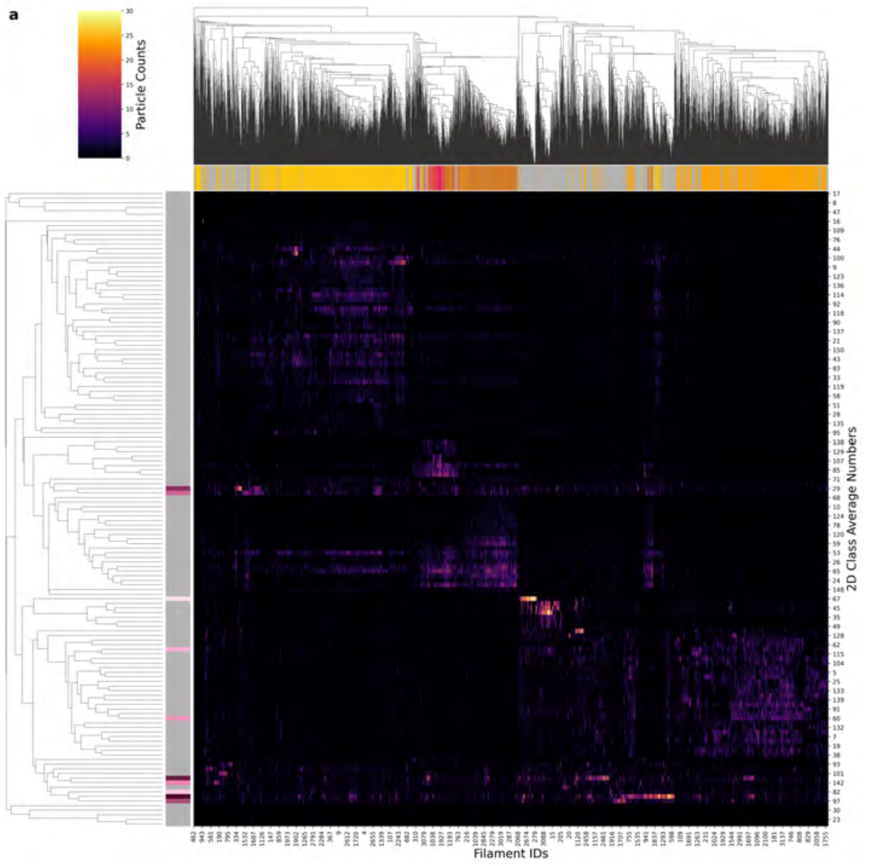

Condition	CTE
Timepoint	360 minutes
Replicate	5
Picking	Manual
Micrographs	1275
Extracted Particles	249442
Pixel Size	0.824 Å
Classified Particles	249442

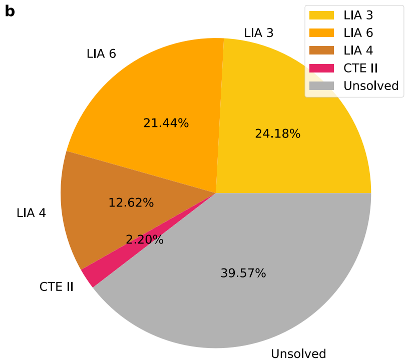

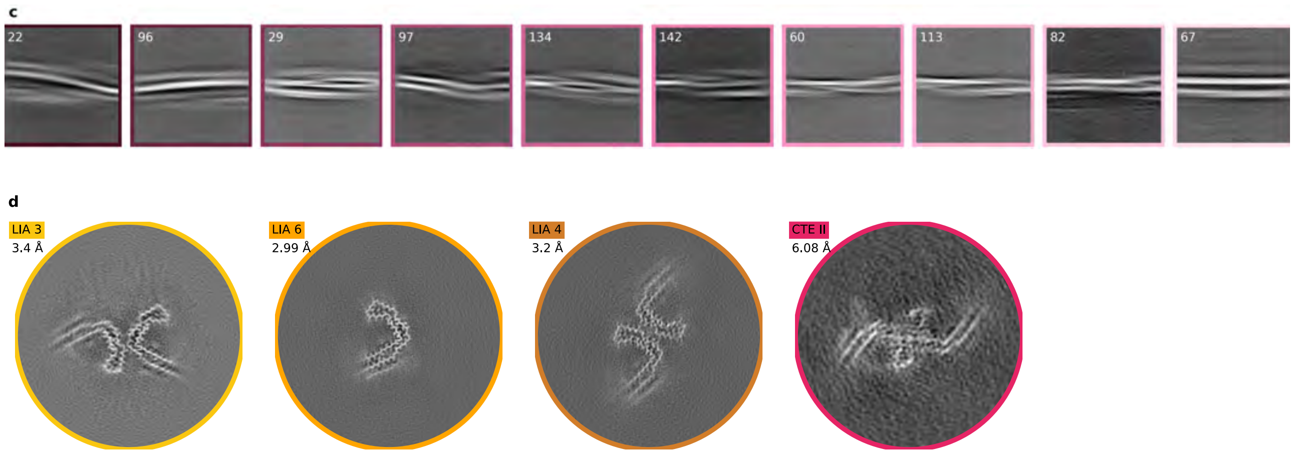

Supplementary Figure 43: Cryo-EM data set summary 43. Data set characteristics are specified in the table on the top right. **a** Hierarchical classification of individual filament segments according to their assigned 2D class average (vertical) and the picked filament ID(horizontal). **b** Pie chart with the relative amounts of different filament types. Grey represents unsolved filaments. Filament types are the same as in Figure 4 of the main text. Different colours represent different time points (120 min in purples; 180 min in blues; 240 min in greens; 300 min in yellows; 360 min in oranges and 720 min in reds). Structures are coloured according to the time point at which they are most abundant, averaged across all replicates. Unique names of filament types are indicated and the same names are used throughout this document. **c** 2D class averages of unsolved filaments. **d** XY-cross-sections, with a projected depth of approximately 4.7 Angstrom or each filament type.

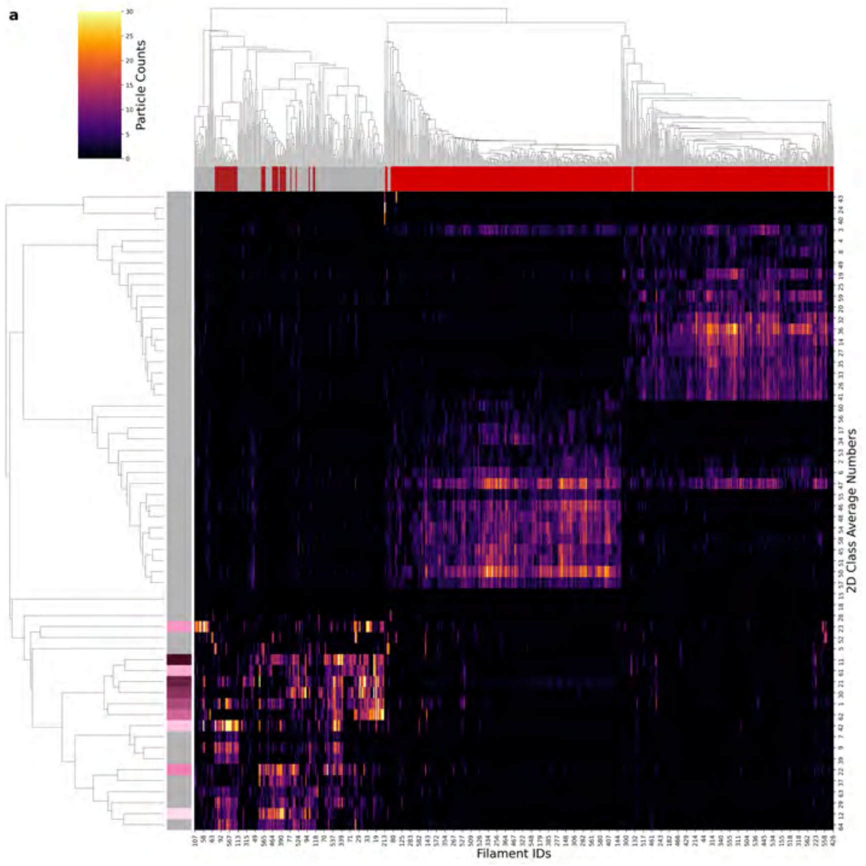

Condition	CTE
Timepoint	720 minutes
Replicate	1
Picking	Manual
Micrographs	1884
Extracted Particles	53403
Pixel Size	0.824 Å
Classified Particles	53403

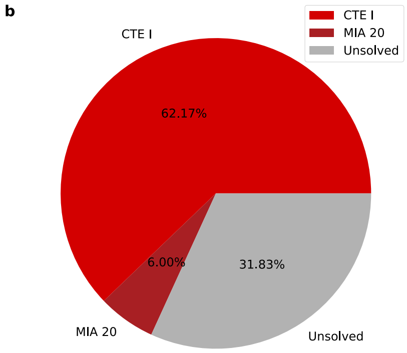

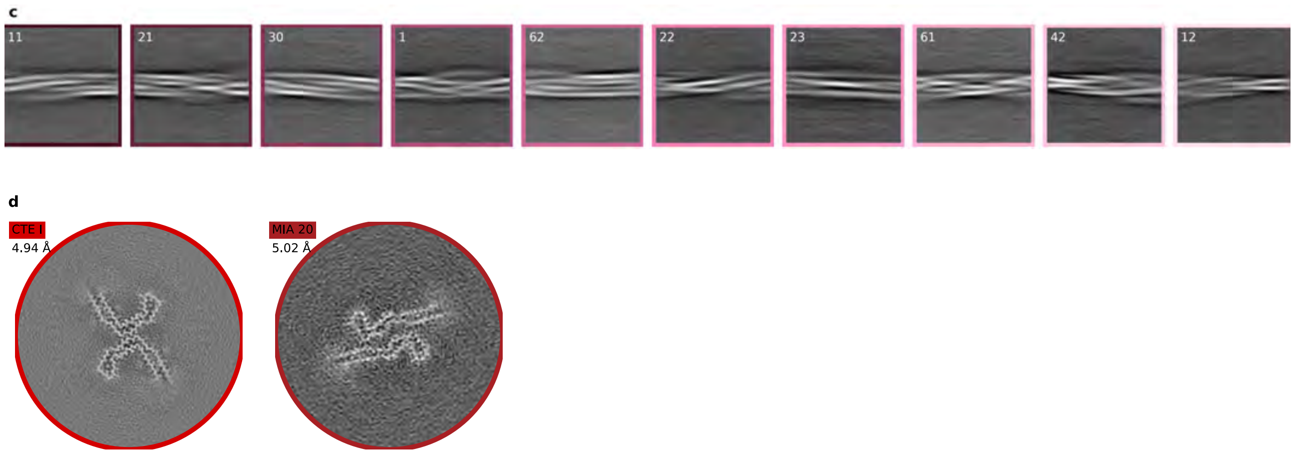

Supplementary Figure 44: Cryo-EM data set summary 44. Data set characteristics are specified in the table on the top right. **a** Hierarchical classification of individual filament segments according to their assigned 2D class average (vertical) and the picked filament ID(horizontal). **b** Pie chart with the relative amounts of different filament types. Grey represents unsolved filaments. Filament types are the same as in Figure 4 of the main text. Different colours represent different time points (120 min in purples; 180 min in blues; 240 min in greens; 300 min in yellows; 360 min in oranges and 720 min in reds). Structures are coloured according to the time point at which they are most abundant, averaged across all replicates. Unique names of filament types are indicated and the same names are used throughout this document. **c** 2D class averages of unsolved filaments. **d** XY-cross-sections, with a projected depth of approximately 4.7 Angstrom or each filament type.

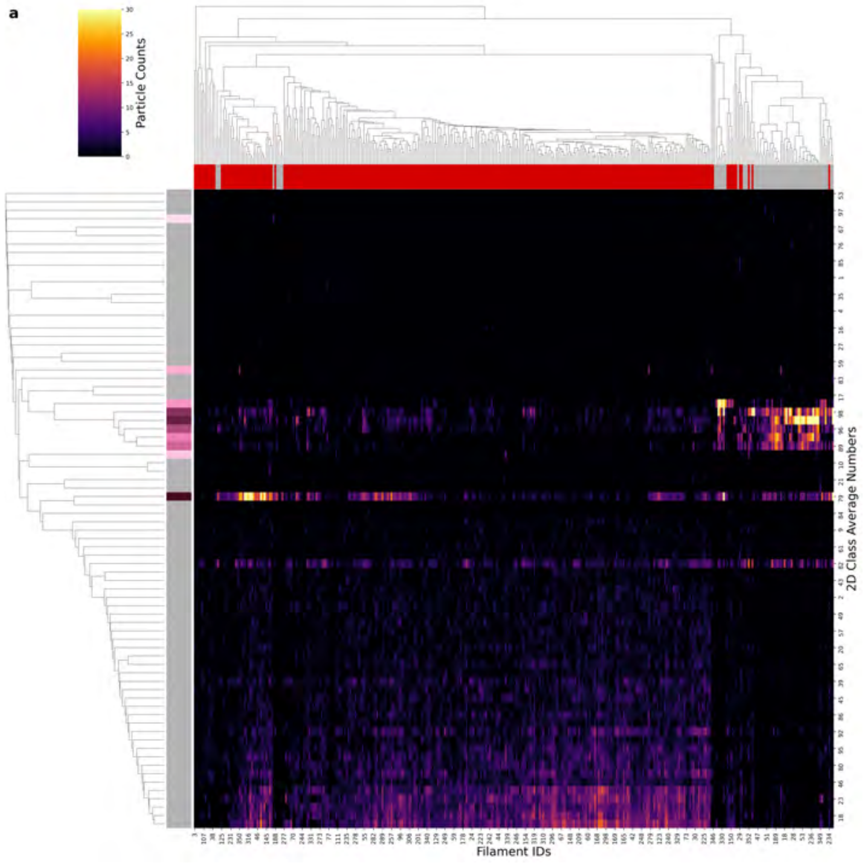

**Condition**	CTE
**Timepoint**	720 minutes
**Replicate**	2
**Picking**	Manual
**Micrographs**	1980
**Extracted Particles**	32022
**Pixel Size**	0.824 Å
**Classified Particles**	32022

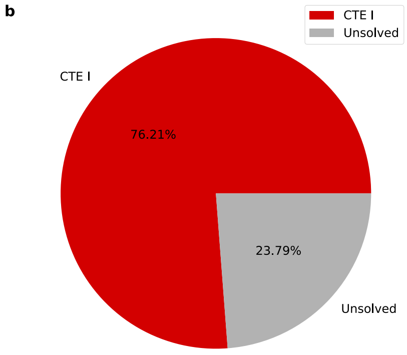

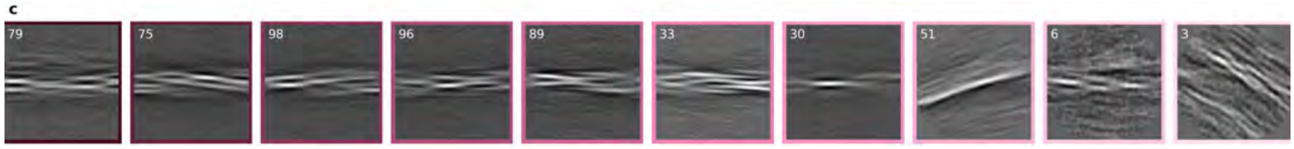

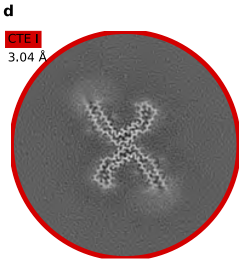

Supplementary Figure 45: Cryo-EM data set summary 45. Data set characteristics are specified in the table on the top right. **a** Hierarchical classification of individual filament segments according to their assigned 2D class average (vertical) and the picked filament ID(horizontal). **b** Pie chart with the relative amounts of different filament types. Grey represents unsolved filaments. Filament types are the same as in Figure 4 of the main text. Different colours represent different time points (120 min in purples; 180 min in blues; 240 min in greens; 300 min in yellows; 360 min in oranges and 720 min in reds). Structures are coloured according to the time point at which they are most abundant, averaged across all replicates. Unique names of filament types are indicated and the same names are used throughout this document. **c** 2D class averages of unsolved filaments. **d** XY-cross-sections, with a projected depth of approximately 4.7 Angstrom or each filament type.

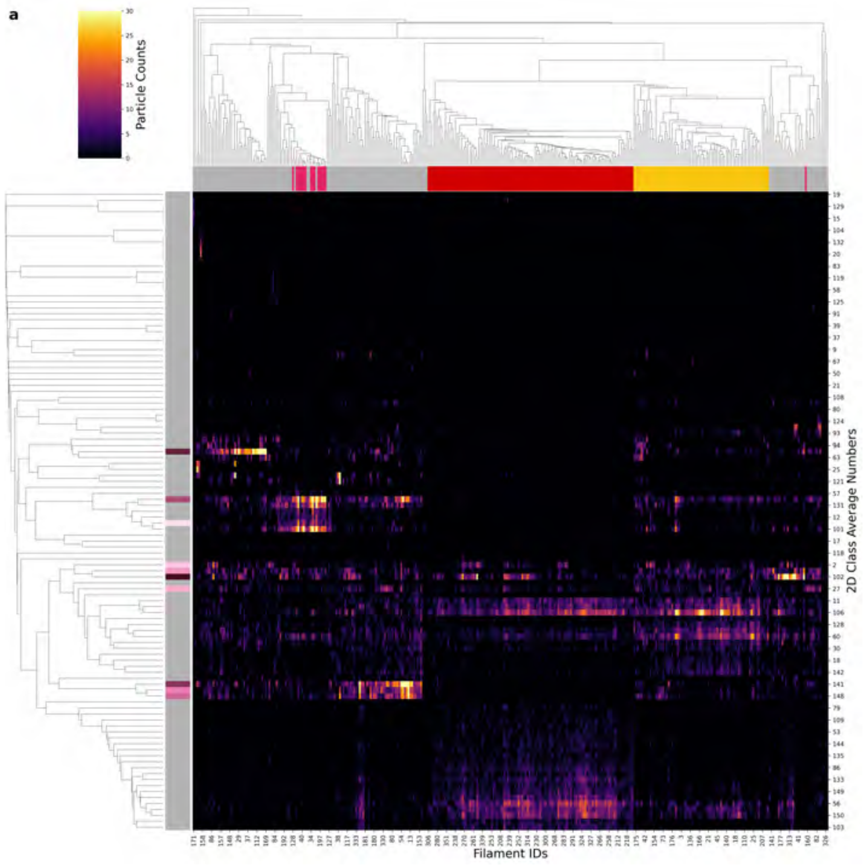

Condition	CTE
Timepoint	720 minutes
Replicate	3
Picking	Manual
Micrographs	1599
Extracted Particles	32580
Pixel Size	0.824 Å
Classified Particles	32580

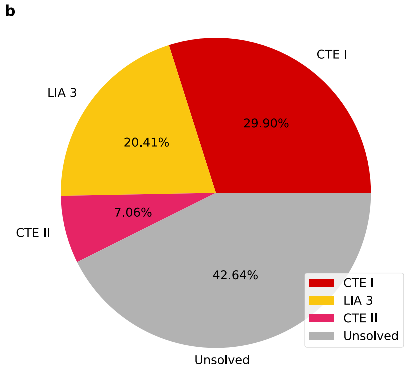

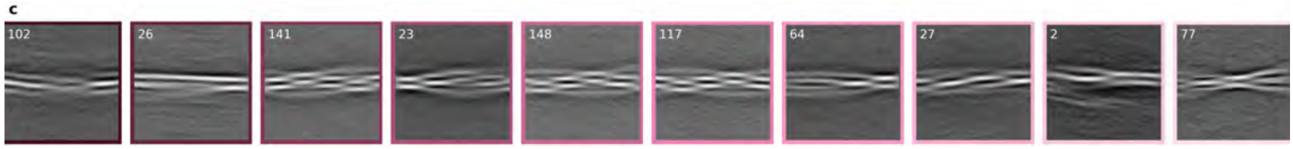

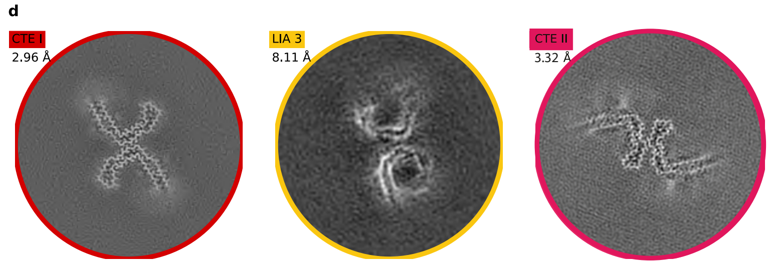

Supplementary Figure 46: Cryo-EM data set summary 46. Data set characteristics are specified in the table on the top right. **a** Hierarchical classification of individual filament segments according to their assigned 2D class average (vertical) and the picked filament ID(horizontal). **b** Pie chart with the relative amounts of different filament types. Grey represents unsolved filaments. Filament types are the same as in Figure 4 of the main text. Different colours represent different time points (120 min in purples; 180 min in blues; 240 min in greens; 300 min in yellows; 360 min in oranges and 720 min in reds). Structures are coloured according to the time point at which they are most abundant, averaged across all replicates. Unique names of filament types are indicated and the same names are used throughout this document. **c** 2D class averages of unsolved filaments. **d** XY-cross-sections, with a projected depth of approximately 4.7 Angstrom or each filament type.

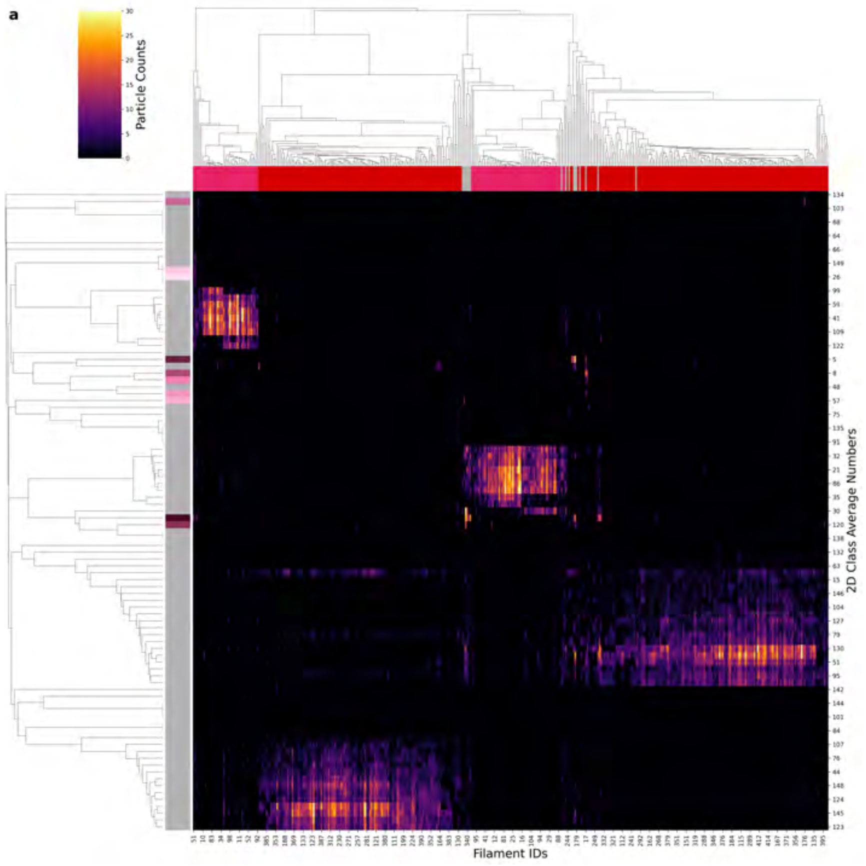

Condition	CTE
Timepoint	720 minutes
Replicate	4
Picking	Manual
Micrographs	2580
Extracted Particles	36037
Pixel Size	0.824 Å
Classified Particles	36037

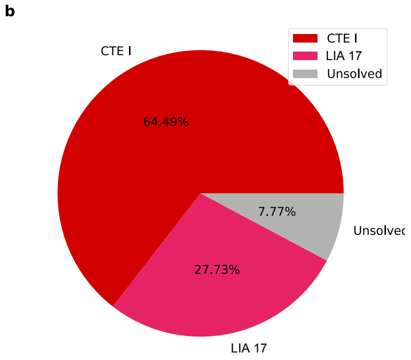

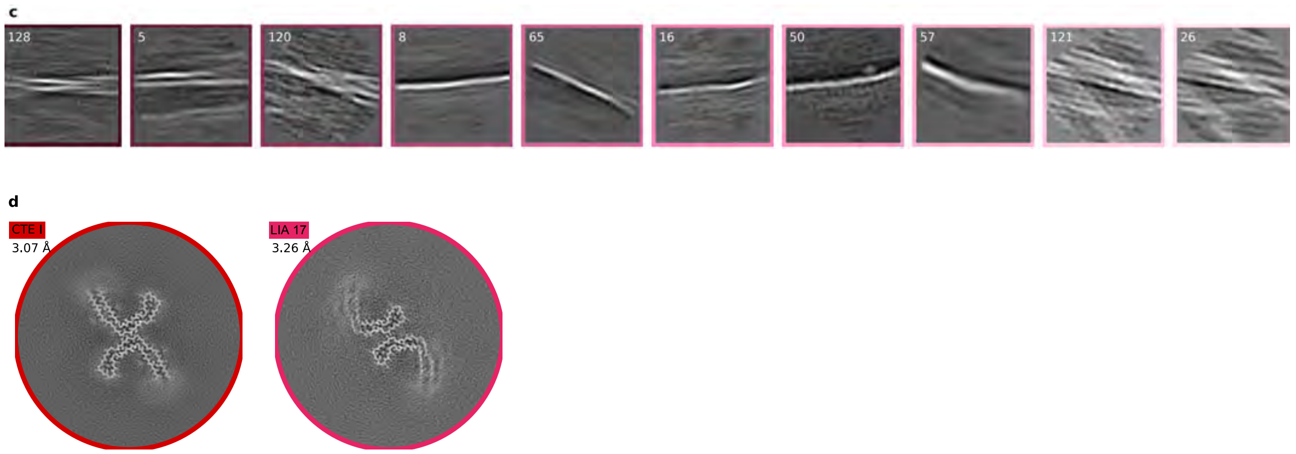

Supplementary Figure 47: Cryo-EM data set summary 47. Data set characteristics are specified in the table on the top right. **a** Hierarchical classification of individual filament segments according to their assigned 2D class average (vertical) and the picked filament ID(horizontal). **b** Pie chart with the relative amounts of different filament types. Grey represents unsolved filaments. Filament types are the same as in Figure 4 of the main text. Different colours represent different time points (120 min in purples; 180 min in blues; 240 min in greens; 300 min in yellows; 360 min in oranges and 720 min in reds). Structures are coloured according to the time point at which they are most abundant, averaged across all replicates. Unique names of filament types are indicated and the same names are used throughout this document. **c** 2D class averages of unsolved filaments. **d** XY-cross-sections, with a projected depth of approximately 4.7 Angstrom or each filament type.

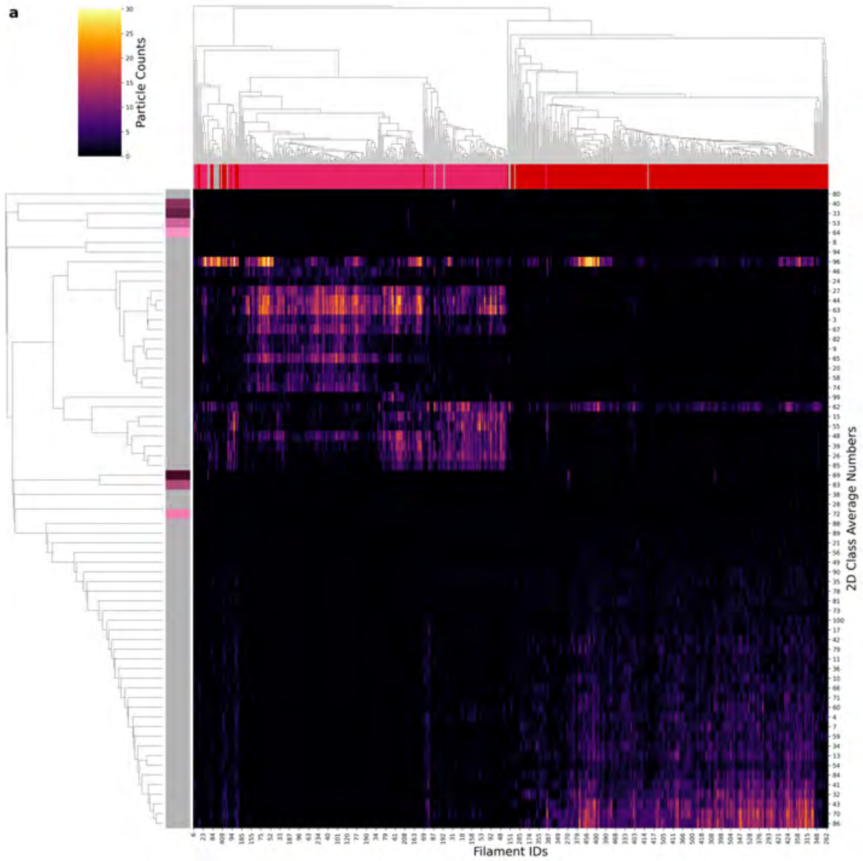

Condition	CTE
Timepoint	720 minutes
Replicate	5
Picking	Manual
Micrographs	2580
Extracted Particles	46371
Pixel Size	0.824 Å
Classified Particles	43926

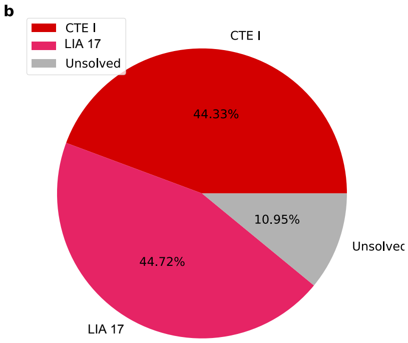

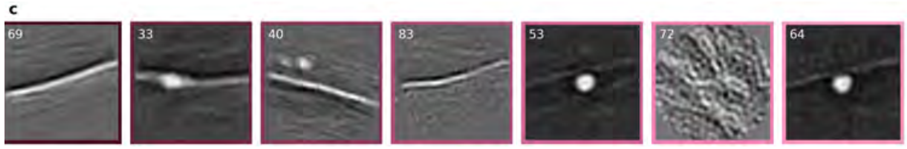

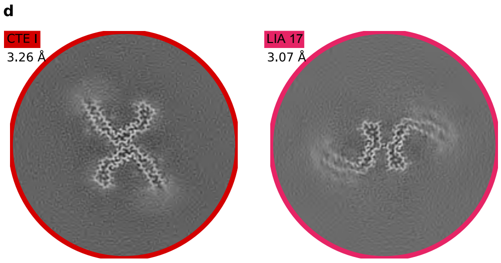

Supplementary Figure 48: Cryo-EM data set summary 48. Data set characteristics are specified in the table on the top right. **a** Hierarchical classification of individual filament segments according to their assigned 2D class average (vertical) and the picked filament ID(horizontal). **b** Pie chart with the relative amounts of different filament types. Grey represents unsolved filaments. Filament types are the same as in Figure 4 of the main text. Different colours represent different time points (120 min in purples; 180 min in blues; 240 min in greens; 300 min in yellows; 360 min in oranges and 720 min in reds). Structures are coloured according to the time point at which they are most abundant, averaged across all replicates. Unique names of filament types are indicated and the same names are used throughout this document. **c** 2D class averages of unsolved filaments. **d** XY-cross-sections, with a projected depth of approximately 4.7 Angstrom or each filament type.

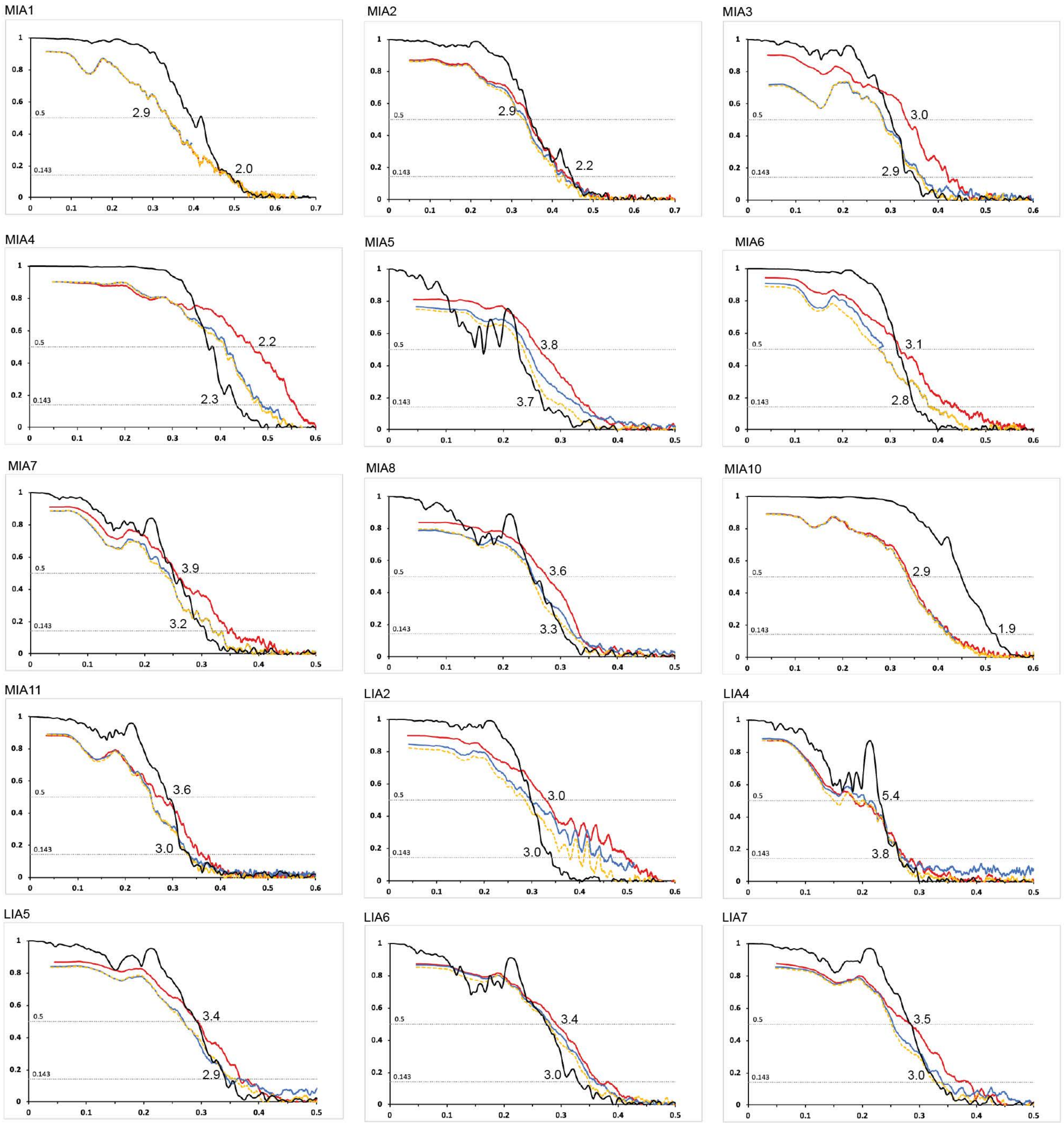

Supplementary Figure 49: Resolution estimates for AD data sets, part 1 For each structure indicated at the top, solvent-corrected Fourier-shell correlation (FSC) curves between independently refined half-maps are shown in black; FSC curves between the refined model and the reconstruction from all particles are shown in red; FSC curves between a model refined against half map 1 against half map 1 are shown in dashed yellow; FSC curves of the same model against half map 2 are shown in blue. Resolution estimates are indicated at thresholds of 0.143 for the half-map FSCs and 0.5 for the model-to-map FSCs.

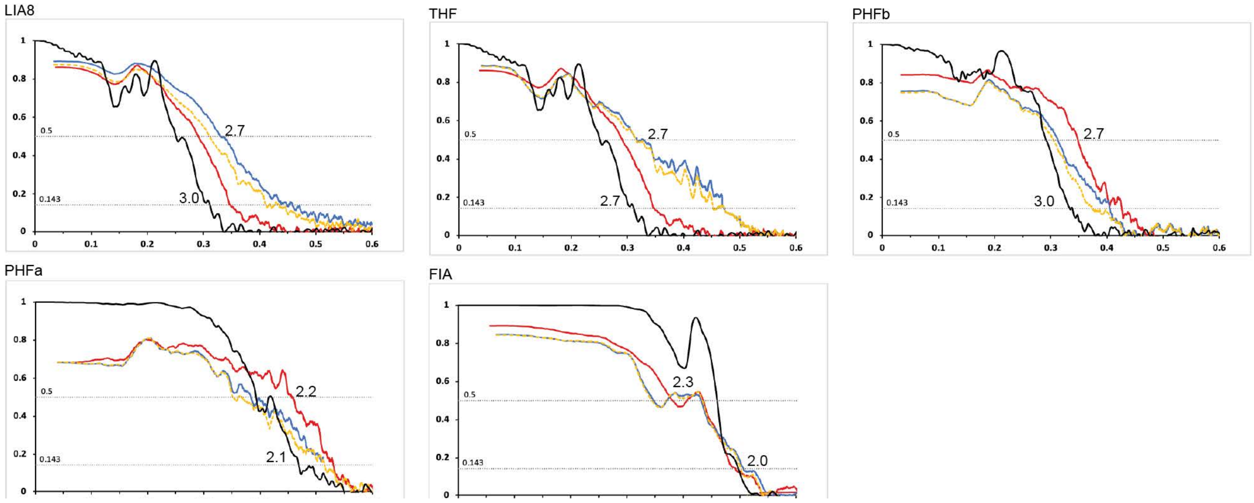

Supplementary Figure 50: Resolution estimates for AD data sets, part 2 For each structure indicated at the top, solvent-corrected Fourier-shell correlation (FSC) curves between independently refined half-maps are shown in black; FSC curves between the refined model and the reconstruction from all particles are shown in red; FSC curves between a model refined against half map 1 against half map 1 are shown in dashed yellow; FSC curves of the same model against half map 2 are shown in blue. Resolution estimates are indicated at thresholds of 0.143 for the half-map FSCs and 0.5 for the model-to-map FSCs.

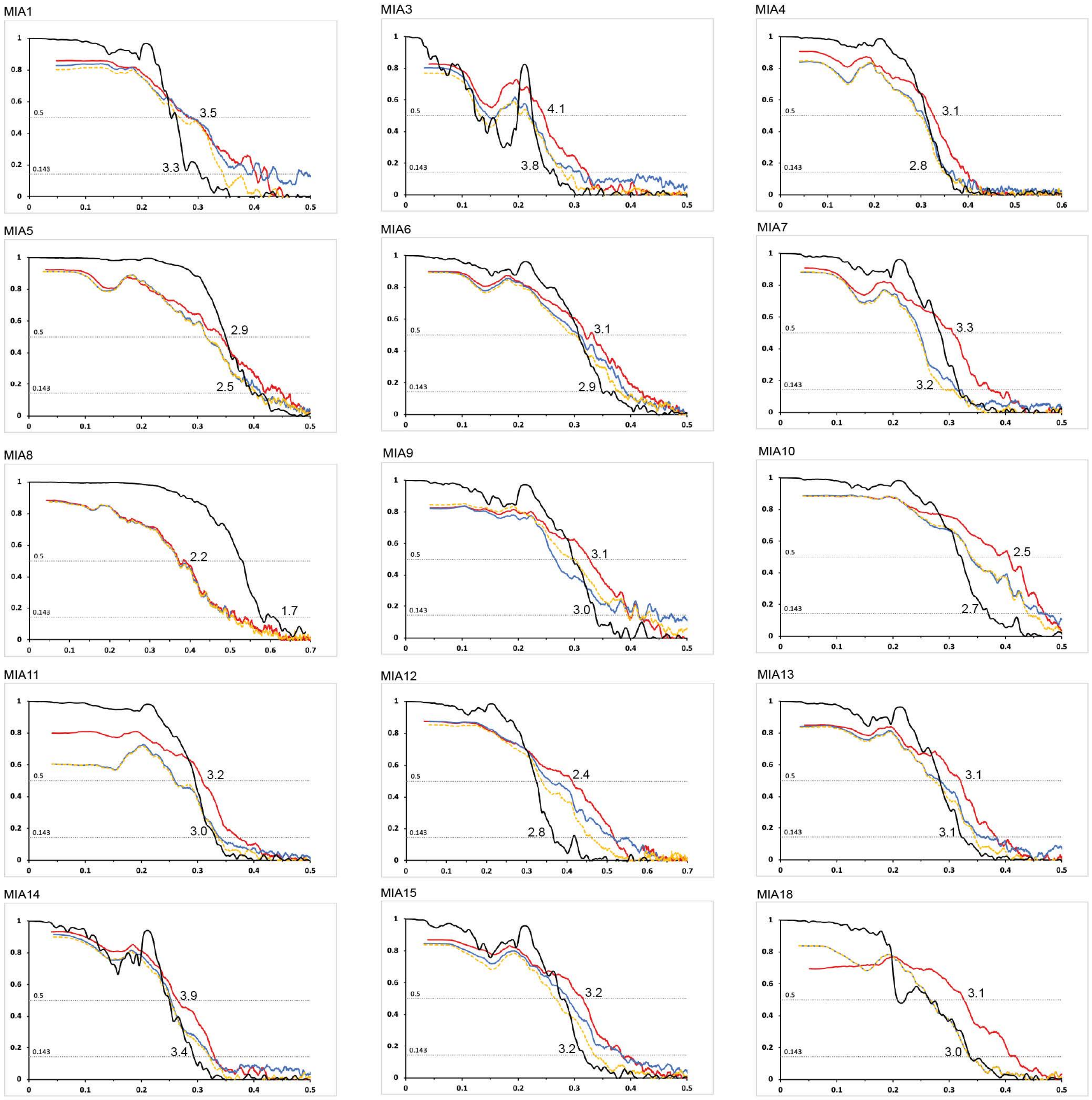

Supplementary Figure 51: Resolution estimates for CTE data sets, part 1 For each structure indicated at the top, solvent-corrected Fourier-shell correlation (FSC) curves between independently refined half-maps are shown in black; FSC curves between the refined model and the reconstruction from all particles are shown in red; FSC curves between a model refined against half map 1 against half map 1 are shown in dashed yellow; FSC curves of the same model against half map 2 are shown in blue. Resolution estimates are indicated at thresholds of 0.143 for the half-map FSCs and 0.5 for the model-to-map FSCs.

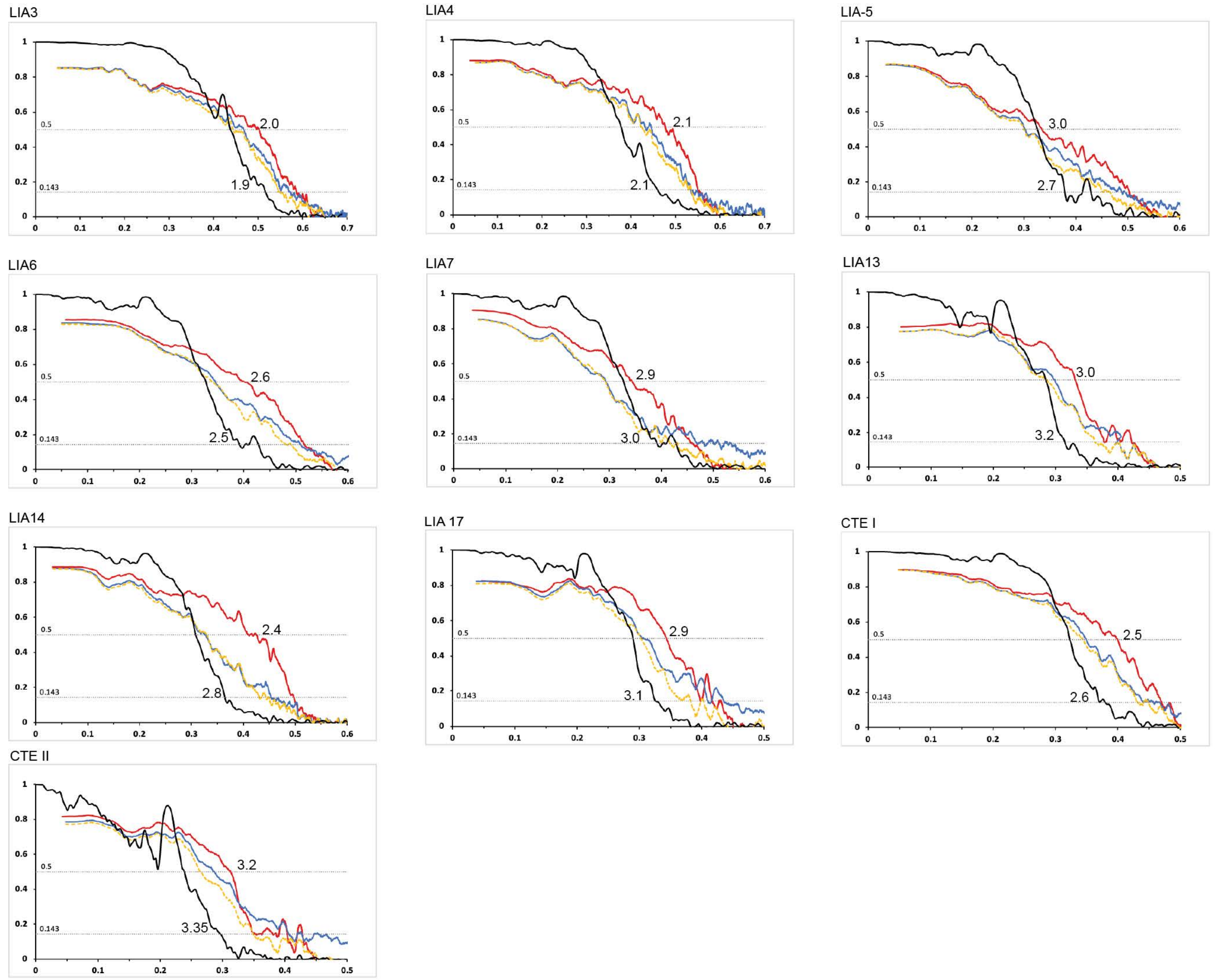

Supplementary Figure 52: Resolution estimates for CTE data sets, part 2 For each structure indicated at the top, solvent-corrected Fourier-shell correlation (FSC) curves between independently refined half-maps are shown in black; FSC curves between the refined model and the reconstruction from all particles are shown in red; FSC curves between a model refined against half map 1 against half map 1 are shown in dashed yellow; FSC curves of the same model against half map 2 are shown in blue. Resolution estimates are indicated at thresholds of 0.143 for the half-map FSCs and 0.5 for the model-to-map FSCs.

	TFS Krios G4	LMB Krios G2
**Data acquisition**		
Electron gun	CFEG	FEG
Detector	Falcon 4i	Falcon 4
Energy filter slit (eV)	10	-
Magnification	165,000 ×	105,000 ×
Voltage (kV)	300	300
Electron dose (e-/Å^2)	40	30
Defocus range (μm)	0.5 to 2.5	0.5 to 2.5

Supplementary Table 1: Data acquisition parameters. For the G4 and G2 Titan Krios microscopes at Thermo Fisher Scientific (TFS) and at the Laboratory of Molecular Biology (LMB), respectively. Statistics about cryo-EM reconstructions are reported separately in the Supplementary Tables below.

**TFS Krios G4**	**AD-FIA**<br>(120' R1)<br>(EMDB 17806)<br>(PDB 8PPO)
**Data acquisition**	
Electron gun	CFEG
Detector	Falcon 4i
Energy filter slit (eV)	10
Magnification	165,000
Voltage (kV)	300
Electron dose (e-/Å^2)	40
Defocus range (µM)	0.5 to 2.5
Pixel size (Å)	0.727
**Data processing**	
Initial particle images (no.)	1680613
Final particle images (no.)	58703
Helical twist (°)	176.886
Helical rise (Å)	2.3667
Symmetry imposed	C1
Map resolution FSC 0.143 (Å)	2.0
**Refinement**	
Initial model used (PDB code)	de novo
Model resolution FSC 0.5 (Å)	2.5
Map sharpening *B* factor (Å^2)	-36.32
Model composition	
Non-hydrogen atoms	1696
Protein residues	240
Ligands	0
B factors (Å^2)	
Protein	35.13
Ligand	na
R.m.s. deviations	
Bond lengths (Å)	0.007
Bond angles (°)	1.197
Validation	
MolProbity score	1.05
Clashscore	0.29
Poor rotamers (%)	0
Ramachandran plot	
Favored (%)	93.27
Allowed (%)	6.73
Disallowed (%)	0

Supplementary Table 2: Refinement statistics 1. Statistics are shown only for the atomic models that were refined in the highest-resolution map.

TFS Krios G4	AD-MIA1 (180' R2) (EMDB 18070) (PDB 8Q27)	AD-MIA2 (180' R2) (EMDB 18109) (PDB 8Q2J)
**Data acquisition**		
Electron gun	CFEG	CFEG
Detector	Falcon 4i	Falcon 4i
Energy filter slit (eV)	10	10
Magnification	165,000	165,000
Voltage (kV)	300	300
Electron dose (e-/Å^2)	40	40
Defocus range (μM)	0.5 to 2.5	0.5-2.5
Pixel size (Å)	0.727	0.727
**Data processing**		
Initial particle images (no.)	138115	138115
Final particle images (no.)	77889	21130
Helical twist (°)	-1.11	-1.22
Helical rise (Å)	4.71	4.75
Symmetry imposed	C1	C1
Map resolution FSC 0.143 (Å)	2.02	2.23
**Refinement**		
Initial model used (PDB code)	ModelAngelo	ModelAngelo
Model resolution FSC 0.5 (Å)	2.9	2.9
Map sharpening *B* factor (Å^2)	-24.9	-25.68
Model composition		
Non-hydrogen atoms	3435	3507
Protein residues	453	462
Ligands	0	0
B factors (Å^2)		
Protein	93.54	96.76
Ligand	na	Na
R.m.s. deviations		
Bond lengths (Å)	0.01	0.01
Bond angles (°)	1.871	1.838
Validation		
MolProbity score	0.83	0.56
Clashscore	0.57	0.14
Poor rotamers (%)	1.27	0
Ramachandran plot		
Favored (%)	97.73	98.22
Allowed (%)	2.27	1.78
Disallowed (%)	0	0

Supplementary Table 3: Refinement statistics 2. Statistics are shown only for the atomic models that were refined in the highest-resolution map.

**TFS Krios G4**	**AD-MIA3** (180' R3) (EMDB 18111) (PDB 8Q2K)	**AD-MIA4** (180' R3) (EMDB 18112) (PDB 8Q2L)
**Data acquisition**		
Electron gun	CFEG	CFEG
Detector	Falcon 4i	Falcon 4i
Energy filter slit (eV)	10	10
Magnification	165,000	165,000
Voltage (kV)	300	300
Electron dose (e-/Å^2)	40	40
Defocus range (μM)	0.5 to 2.5	0.5 to 2.5
Pixel size (Å)	0.727	0.727
**Data processing**		
Initial particle images (no.)	159594	159594
Final particle images (no.)	13033	94975
Helical twist (°)	-1.29	-1.44
Helical rise (Å)	4.75	4.74
Symmetry imposed	C1	C2
Map resolution FSC 0.143 (Å)	2.88	2.32
**Refinement**		
Initial model used (PDB code)	de novo	ModelAngelo
Model resolution FSC 0.5 (Å)	3.0	2.2
Map sharpening *B* factor (Å^2)	-57.56	-62.15
Model composition		
Non-hydrogen atoms	3444	3354
Protein residues	450	438
Ligands	0	0
B factors (Å^2)		
Protein	96.09	40.74
Ligand	na	na
R.m.s. deviations		
Bond lengths (Å)	0.011	0.012
Bond angles (°)	1.884	2.330
Validation		
MolProbity score	0.94	0.86
Clashscore	0.43	1.32
Poor rotamers (%)	0.00	0
Ramachandran plot		
Favored (%)	95.89	98.12
Allowed (%)	4.11	1.88
Disallowed (%)	0	0

Supplementary Table 4: Refinement statistics 3. Statistics are shown only for the atomic models that were refined in the highest-resolution map.

TFS Krios G4	AD-MIA5 (240’ R3) (EMDB 18215) (PDB 8Q7F)	AD-MIA8 (240’ R3) (EMDB 18228) (PDB 8Q7P)	AD-LIA8 (240’ R3) (EMDB 18331) (PDB 8QCP)
**Data acquisition**			
Electron gun	CFEG	CFEG	CFEG
Detector	Falcon 4i	Falcon 4i	Falcon 4i
Energy filter slit (eV)	10	10	10
Magnification	165,000	165,000	165,000
Voltage (kV)	300	300	300
Electron dose (e-/Å^2)	40	40	40
Defocus range (μM)	0.5 to 2.5	0.5 to 2.5	0.5 to 2.5
Pixel size (Å)	0.727	0.727	0.727
**Data processing**			
Initial particle images (no.)	80292	80292	80292
Final particle images (no.)	2111	4226	4013
Helical twist (º)	-1.32	-1.11	-1.24
Helical rise (Å)	4.74	4.77	4.75
Symmetry imposed	C1	C1	C1
Map resolution FSC 0.143 (Å)	3.72	3.28	3.21
**Refinement**			
Initial model used (PDB code)	de novo	de novo	de novo
Model resolution FSC 0.5 (Å)	3.8	3.6	3.30
Map sharpening *B* factor (Å^2)	-47.87	-37.31	-33.55
Model composition			
Non-hydrogen atoms	3402	3402	3435
Protein residues	450	450	453
Ligands	0	0	0
B factors (Å^2)			
Protein	95.31	95.31	94.92
Ligand	na	na	na
R.m.s. deviations			
Bond lengths (Å)	0.011	0.010	0.011
Bond angles (°)	1.967	1.943	1.929
Validation			
MolProbity score	0.50	0.50	1.02
Clashscore	0	0	0.14
Poor rotamers (%)	0.51	0.00	0
Ramachandran plot			
Favored (%)	98.86	98.63	92.52
Allowed (%)	1.14	1.37	7.48
Disallowed (%)	0	0	0.00

Supplementary Table 5: Refinement statistics 4. Statistics are shown only for the atomic models that were refined in the highest-resolution map.

LMB Krios G2	AD-MIA6 (240' R1) (EMDB 18219) (PDB 8Q7L)	AD-MIA7 (240' R1) (EMDB 18224) (PDB 8Q7M)	AD-LIA1 (240' R1) (EMDB 18344)	AD-LIA2 (240' R1) (EMDB 18249) (PDB 8Q88)
**Data acquisition**				
Electron gun	FEG	FEG	FEG	FEG
Detector	Falcon 4	Falcon 4	Falcon 4	Falcon 4
Energy filter slit (eV)	na	na	na	na
Magnification	105,000	105,000	105,000	105,000
Voltage (kV)	300	300	300	300
Electron dose (e-/Å^2)	30	30	30	30
Defocus range (μM)	0.5 to 2.5	0.5 to 2.5	0.5 to 2.5	0.5 to 2.5
Pixel size (Å)	0.824	0.824	0.824	0.824
**Data processing**				
Initial particle images (no.)	352893	352893	352893	352893
Final particle images (no.)	125479	7632	7532	88413
Helical twist (°)	-1.06	-0.77	-0.81	-0.82
Helical rise (Å)	4.74	4.77	4.75	4.77
Symmetry imposed	C1	C1	C1	C1
Map resolution FSC 0.143 (Å)	2.82	3.26	7.19	2.95
**Refinement**				
Initial model used (PDB code)	de novo	de novo		de novo
Model resolution FSC 0.5 (Å)	3.1	3.9		3.0
Map sharpening *B* factor (Å^2)	-72.91	-45.89		-78.18
Model composition				
Non-hydrogen atoms	3375	5109		3468
Protein residues	441	669		456
Ligands	0	0		0
B factors (Å^2)				
Protein	93.83	78.97		50.07
Ligand	na	na		na
R.m.s. deviations				
Bond lengths (Å)	0.010	0.010		0.010
Bond angles (°)	1.999	2.036		1.879
Validation				
MolProbity score	1.34	0.85		1.06
Clashscore	2.47	0.48		0.85
Poor rotamers (%)	0	0.17		0.25
Ramachandran plot				
Favored (%)	95.57	96.93		95.72

Supplementary Table 6: Refinement statistics 5. Statistics are shown only for the atomic models that were refined in the highest-resolution map.

TFS Krios G4	AD-MIA10 (240’ R5) (EMDB 18250) (PDB 8Q8C)	AD-MIA11 (240’ R5) (EMDB 18233) (PDB 8Q7T)
**Data acquisition**		
Electron gun	CFEG	CFEG
Detector	Falcon 4i	Falcon 4i
Energy filter slit (eV)	10	10
Magnification	165,000	165,000
Voltage (kV)	300	300
Electron dose (e-/Å^2)	40	40
Defocus range (μM)	0.5 to 2.5	0.5 to 2.5
Pixel size (Å)	0.727	0.727
**Data processing**		
Initial particle images (no.)	1178114	1178114
Final particle images (no.)	744002	17410
Helical twist (º)	-1.08	-0.99
Helical rise (Å)	4.74	4.76
Symmetry imposed	C1	C1
Map resolution FSC 0.143 (Å)	1.92	3.00
**Refinement**		
Initial model used (PDB code)	de novo	de novo
Model resolution FSC 0.5 (Å)	2.9	3.6
Map sharpening *B* factor (Å^2)	-32.0	-54.81
Model composition		
Non-hydrogen atoms	3246	4947
Protein residues	429	654
Ligands	0	0
B factors (Å^2)		
Protein	96.32	95.98
Ligand	na	na
R.m.s. deviations		
Bond lengths (Å)	0.011	0.010
Bond angles (°)	1.790	1.895
Validation		
MolProbity score	0.78	0.80
Clashscore	0.91	0.69
Poor rotamers (%)	0	0
Ramachandran plot		
Favored (%)	98.80	97.64
Allowed (%)	1.20	2.36
Disallowed (%)	0	0

Supplementary Table 7: Refinement statistics 6. Statistics are shown only for the atomic models that were refined in the highest-resolution map.

LMB Krios G2	AD-LIA4 (300' R4) (EMDB 18252) (PDB 8Q8E)
**Data acquisition**	
Electron gun	FEG
Detector	Falcon 4
Energy filter slit (eV)	na
Magnification	105,000
Voltage (kV)	300
Electron dose (e-/Å^2)	30
Defocus range (μM)	0.5 to 2.5
Pixel size (Å)	0.824
**Data processing**	
Initial particle images (no.)	100371
Final particle images (no.)	6032
Helical twist (°)	179.61
Helical rise (Å)	2.38
Symmetry imposed	C1
Map resolution FSC 0.143 (Å)	3.81
**Refinement**	
Initial model used (PDB code)	de novo
Model resolution FSC 0.5 (Å)	5.4
Map sharpening *B* factor (Å^2)	-48.66
Model composition	
Non-hydrogen atoms	6810
Protein residues	894
Ligands	0
B factors (Å^2)	
Protein	93.55
Ligand	na
R.m.s. deviations	
Bond lengths (Å)	0.011
Bond angles (°)	2.082
Validation	
MolProbity score	1.81
Clashscore	5.47
Poor rotamers (%)	0
Ramachandran plot	
Favored (%)	91.03
Allowed (%)	9.97
Disallowed (%)	0

Supplementary Table 8: Refinement statistics 7. Statistics are shown only for the atomic models that were refined in the highest-resolution map.

LMB Krios G2	AD-LIA6 (300’ R2) (EMDB 18251) (PDB 8Q8D)	AD-LIA5 (300’ R2) (EMDB 18253) (PDB 8Q8F)
**Data acquisition**		
Electron gun	FEG	FEG
Detector	Falcon 4	Falcon 4
Energy filter slit (eV)	na	na
Magnification	105,000	105,000
Voltage (kV)	300	300
Electron dose (e-/Å^2)	30	30
Defocus range (μM)	1.0 to 2.5	1.0 to 2.5
Pixel size (Å)	0.824	0.824
**Data processing**		
Initial particle images (no.)	119480	119480
Final particle images (no.)	4959	9294
Helical twist (º)	-1.07	-1.07
Helical rise (Å)	4.766	4.766
Symmetry imposed	C1	C1
Map resolution FSC 0.143 (Å)	3.04	2.93
**Refinement**		
Initial model used (PDB code)	ModelAngelo	ModelAngelo
Model resolution FSC 0.5 (Å)	3.4	3.4
Map sharpening *B* factor (Å^2)	-27.03	-30.40
Model composition		
Non-hydrogen atoms	3468	3468
Protein residues	456	456
Ligands	0	0
B factors (Å^2)		
Protein	92.45	92.45
Ligand	na	na
R.m.s. deviations		
Bond lengths (Å)	0.01	0.01
Bond angles (°)	1.946	1.913
Validation		
MolProbity score	0.66	0.93
Clashscore	0.00	0.14
Poor rotamers (%)	0	0.76
Ramachandran plot		
Favored (%)	97.07	94.59
Allowed (%)	2.93	5.49
Disallowed (%)	0	0

Supplementary Table 9: Refinement statistics 8. Statistics are shown only for the atomic models that were refined in the highest-resolution map.

**LMB Krios G2**	**AD-LIA7** (300' R3) (EMDB 18254) (PDB 8Q8L)	**AD-PHFb** (300' R3) (EMDB 18255) (PDB 8Q8M)	**AD-QHF** (300' R3) (EMDB 18349)
**Data acquisition**			
Electron gun	FEG	FEG	FEG
Detector	Falcon 4	Falcon 4	Falcon 4
Energy filter slit (eV)	na	na	na
Magnification	105,000	105,000	105,000
Voltage (kV)	300	300	300
Electron dose (e-/Å^2)	30	30	30
Defocus range (μM)	0.5 to 2.5	0.5 to 2.5	0.5 to 2.5
Pixel size (Å)	0.824	0.824	0.824
**Data processing**			
Initial particle images (no.)	495698	495698	495698
Final particle images (no.)	24999	109455	17256
Helical twist (°)	-1.05	179.86	-0.78
Helical rise (Å)	4.77	2.394	4.75
Symmetry imposed	C1	C1	C1
Map resolution FSC 0.143 (Å)	3.04	2.95	4.28
**Refinement**			
Initial model used (PDB code)	de novo	de novo	
Model resolution FSC 0.5 (Å)	3.51	2.7	
Map sharpening *B* factor (Å^2)	-46.92	-31.86	
Model composition			
Non-hydrogen atoms	3405	3468	
Protein residues	447	456	
Ligands	0	0	
B factors (Å^2)			
Protein	93.55	50.07	
Ligand	na	na	
R.m.s. deviations			
Bond lengths (Å)	0.011	0.010	
Bond angles (°)	2.141	2.029	
Validation			
MolProbity score	1.07	0.97	
Clashscore	0.58	0.00	
Poor rotamers (%)	0	0	
Ramachandran plot			
Favored (%)	94.48	92.57	

Supplementary Table 10: Refinement statistics 9. Statistics are shown only for the atomic models that were refined in the highest-resolution map.

TFS Krios G4	AD-LIA3 (360’ R1) (EMDB 18347)	AD-THF (360’ R1) (EMDB 18259) (PDB 8Q8S)
**Data acquisition**		
Electron gun	CFEG	CFEG
Detector	Falcon 4i	Falcon 4i
Energy filter slit (eV)	10	10
Magnification	165,000	165,000
Voltage (kV)	300	300
Electron dose (e-/Å^2)	40	40
Defocus range (µM)	0.5 to 2.5	0.5 to 2.5
Pixel size (Å)	0.727	0.727
**Data processing**		
Initial particle images (no.)	382080	382080
Final particle images (no.)	6568	60376
Helical twist (º)	-0.7	-0.86
Helical rise (Å)	4.75	4.75
Symmetry imposed	C1	C1
Map resolution FSC 0.143 (Å)	9.00	2.68
**Refinement**		
Initial model used (PDB code)		de novo
Model resolution FSC 0.5 (Å)		2.7
Map sharpening *B* factor (Å^2)		-39.98
Model composition		
Non-hydrogen atoms		5202
Protein residues		684
Ligands		0
B factors (Å^2)		
Protein		50.03
Ligand		na
R.m.s. deviations		
Bond lengths (Å)		0.011
Bond angles (°)		2.224
Validation		
MolProbity score		1.22
Clashscore		0.85
Poor rotamers (%)		0
Ramachandran plot		

Supplementary Table 11: Refinement statistics 10. Statistics are shown only for the atomic models that were refined in the highest-resolution map.

LMB Krios G2	AD-MIA9 (720' R3) (EMDB 18348)
**Data acquisition**	
Electron gun	FEG
Detector	Falcon 4
Energy filter slit (eV)	na
Magnification	105,000
Voltage (kV)	300
Electron dose (e-/Å^2)	30
Defocus range (μM)	0.5 to 2.5
Pixel size (Å)	0.824
**Data processing**	
Initial particle images (no.)	175528
Final particle images (no.)	5242
Helical twist (°)	-1.09
Helical rise (Å)	4.79
Symmetry imposed	C1
Map resolution FSC 0.143 (Å)	7.34
**Refinement**	
Initial model used (PDB code)	
Model resolution FSC 0.5 (Å)	
Map sharpening *B* factor (Å^2)	
Model composition	
Non-hydrogen atoms	
Protein residues	
Ligands	
B factors (Å^2)	
Protein	
Ligand	
R.m.s. deviations	
Bond lengths (Å)	
Bond angles (°)	
Validation	
MolProbity score	
Clashscore	
Poor rotamers (%)	
Ramachandran plot	
Favored (%)	

Supplementary Table 12: Refinement statistics 11. Statistics are shown only for the atomic models that were refined in the highest-resolution map.

TFS Krios G4	AD-PHFa (EMDB 18258) (PDB 8Q8R)
**Data acquisition**	
Electron gun	CFEG
Detector	Falcon 4i
Energy filter slit (eV)	10
Magnification	165,000
Voltage (kV)	300
Electron dose (e-/Å^2)	40
Defocus range (μM)	0.5 to 2.5
Pixel size (Å)	0.727
**Data processing**	
Initial particle images (no.)	125865
Final particle images (no.)	72387
Helical twist (°)	179.494
Helical rise (Å)	2.38
Symmetry imposed	C1
Map resolution FSC 0.143 (Å)	2.1
**Refinement**	
Initial model used (PDB code)	de novo
Model resolution FSC 0.5 (Å)	2.2
Map sharpening *B* factor (Å^2)	-50.2
Model composition	
Non-hydrogen atoms	3468
Protein residues	456
Ligands	0
B factors (Å^2)	
Protein	50.01
Ligand	na
R.m.s. deviations	
Bond lengths (Å)	0.011
Bond angles (°)	1.911
Validation	
MolProbity score	1.08
Clashscore	0.29
Poor rotamers (%)	0
Ramachandran plot	
Favored (%)	88.96
Allowed (%)	11.04
Disallowed (%)	0

Supplementary Table 13: Refinement statistics 12. Statistics are shown only for the atomic models that were refined in the highest-resolution map.

LMB Krios G2	CTE-MIA1 (160' R4) (EMDB 18261) (PDB 8Q8U)	CTE-MIA2 (160' R4) (EMDB 18354)	CTE-LIA11 (160' R4) (EMDB 18363)
**Data acquisition**			
Electron gun	FEG	FEG	FEG
Detector	Falcon 4	Falcon 4	Falcon 4
Energy filter slit (eV)	na	na	na
Magnification	105,000	105,000	105,000
Voltage (kV)	300	300	300
Electron dose (e-/Å^2)	30	30	30
Defocus range (μM)	0.5 to 2.5	0.5 to 2.5	0.5 to 2.5
Pixel size (Å)	0.824	0.824	0.824
**Data processing**			
Initial particle images (no.)	59766	59766	59766
Final particle images (no.)	20639	4900	5612
Helical twist (°)	-1.23	-1.22	-1.32
Helical rise (Å)	4.77	4.75	4.75
Symmetry imposed	C2	C1	C1
Map resolution FSC 0.143 (Å)	3.3	4.22	8.11
**Refinement**			
Initial model used (PDB code)	de novo		
Model resolution FSC 0.5 (Å)	3.5		
Map sharpening *B* factor (Å^2)	-52.74		
Model composition			
Non-hydrogen atoms	3246		
Protein residues	426		
Ligands	0		
B factors (Å^2)			
Protein	39.48		
Ligand	na		
R.m.s. deviations			
Bond lengths (Å)	0.011		
Bond angles (°)	2.006		
Validation			
MolProbity score	0.92		
Clashscore	0.00		
Poor rotamers (%)	0		
Ramachandran plot			
Favored (%)	93.73		
Allowed (%)	6.28		
Disallowed (%)	0		

Supplementary Table 14: Refinement statistics 13. Statistics are shown only for the atomic models that were refined in the highest-resolution map.

LMB Krios G2	CTE-MIA3 (160’ R5) (EMDB 18262) (PDB 8Q8V)	CTE-MIA12 (160’ R5) (EMDB 18333) (PDB 8QCR)
**Data acquisition**		
Electron gun	FEG	CFEG
Detector	Falcon 4	Falcon 4
Energy filter slit (eV)	na	na
Magnification	105,000	105,000
Voltage (kV)	300	300
Electron dose (e-/Å^2)	30	30
Defocus range (μM)	0.5 to 2.5	0.5 to 2.5
Pixel size (Å)	0.824	0.824
**Data processing**		
Initial particle images (no.)	158080	158080
Final particle images (no.)	4243	44288
Helical twist (°)	-1.13	-1.39
Helical rise (Å)	4.77	4.77
Symmetry imposed	C1	C2
Map resolution FSC 0.143 (Å)	3.8	2.75
**Refinement**		
Initial model used (PDB code)	de novo	de novo
Model resolution FSC 0.5 (Å)	4.1	2.4
Map sharpening *B* factor (Å^2)	-25.39	-34.96
Model composition		
Non-hydrogen atoms	3444	1545
Protein residues	450	204
Ligands	0	0
B factors (Å^2)		
Protein	94.85	97.44
Ligand	na	na
R.m.s. deviations		
Bond lengths (Å)	0.011	0.01
Bond angles (°)	2.018	1.856
Validation		
MolProbity score	1.39	0.89
Clashscore	1.99	0.63
Poor rotamers (%)	0	0
Ramachandran plot		
Favored (%)	93.61	96.97
Allowed (%)	6.39	3.03
Disallowed (%)	0	0

Supplementary Table 15: Refinement statistics 14. Statistics are shown only for the atomic models that were refined in the highest-resolution map.

LMB Krios G2	CTE-MIA4 (180' R4) (EMDB 18263) (PDB 8Q8W)
**Data acquisition**	
Electron gun	FEG
Detector	Falcon 4
Energy filter slit (eV)	na
Magnification	105,000
Voltage (kV)	300
Electron dose (e-/Å^2)	30
Defocus range (μM)	1.0 to 2.5
Pixel size (Å)	0.824
**Data processing**	
Initial particle images (no.)	66426
Final particle images (no.)	42082
Helical twist (°)	-1.09
Helical rise (Å)	4.73
Symmetry imposed	C1
Map resolution FSC 0.143 (Å)	2.85
**Refinement**	
Initial model used (PDB code)	de novo
Model resolution FSC 0.5 (Å)	3.1
Map sharpening *B* factor (Å^2)	-50.89
Model composition	
Non-hydrogen atoms	3246
Protein residues	429
Ligands	0
B factors (Å^2)	
Protein	96.32
Ligand	na
R.m.s. deviations	
Bond lengths (Å)	0.010
Bond angles (°)	1.863
Validation	
MolProbity score	0.82
Clashscore	0.45
Poor rotamers (%)	0.00
Ramachandran plot	
Favored (%)	97.12
Allowed (%)	2.88
Disallowed (%)	0

Supplementary Table 16: Refinement statistics 15. Statistics are shown only for the atomic models that were refined in the highest-resolution map.

TFS Krios G4	**CTE-MIA5** (180’ R1) (EMDB 18264) (PDB 8Q8X)	**CTE-MIA6** (180’ R1) (EMDB 18265) (PDB 8Q8Y)	**CTE-MIA8** (180’ R1) (EMDB 18271) (PDB 8Q98)	**CTE-MIA18** (180’ R1) (EMDB 18278) (PDB 8Q9E)
**Data acquisition**				
Electron gun	CFEG	CFEG	CFEG	CFEG
Detector	Falcon 4i	Falcon 4i	Falcon 4i	Falcon 4i
Energy filter slit (eV)	10	10	10	10
Magnification	165,000	165,000	165,000	165,000
Voltage (kV)	300	300	300	300
Electron dose (e-/Å^2)	40	40	40	40
Defocus range (μM)	1.0 to 2.5	1.0 to 2.5	0.5 to 2.5	0.5 to 2.5
Pixel size (Å)	0.727	0.727	0.727	0.727
**Data processing**				
Initial particle images (no.)	960020	960020	960020	960020
Final particle images (no.)	177452	12971	37333	27343
Helical twist (°)	-1.08	-1.12	-1.31	-1.65
Helical rise (Å)	4.74	4.71	4.75	4.74
Symmetry imposed	C1	C1	C2	C1
Map resolution FSC 0.143 (Å)	2.54	2.88	1.75	2.97
**Refinement**				
Initial model used (PDB code)	de novo	de novo	ModelAngelo	ModelAngelo
Model resolution FSC 0.5 (Å)	2.9	3.1	2.7	3.1
Map sharpening *B* factor (Å^2)	-65.69	-50.06	-19.86	-65.20
Model composition				
Non-hydrogen atoms	3435	3375	3444	3312
Protein residues	453	441	450	432
Ligands	0	0	0	0
B factors (Å^2)				
Protein	93.54	93.83	96.09	94.68
Ligand	na	na	na	na
R.m.s. deviations				
Bond lengths (Å)	0.011	0.011	0.01	0.01
Bond angles (°)	1.871	1.989	1.822	1.960
Validation				
MolProbity score	0.87	0.68	0.50	0.92
Clashscore	0.57	0.29	0.00	1.18
Poor rotamers (%)	1.27	0	0	1.32
Ramachandran plot				
Favored (%)	97.51	97.67	99.54	98.10
Allowed (%)	2.49	2.33	0.46	1.90
Disallowed (%)	0	0	0	0

Supplementary Table 17: Refinement statistics 16. Statistics are shown only for the atomic models that were refined in the highest-resolution map.

LMB Krios G2	CTE-MIA7 (240’ R3) (EMDB 18266) (PDB 8Q8Z)	CTE-MIA13 (240’ R3) (EMDB 18275) (PDB 8Q9B)	CTE-MIA14 (240’ R3) (EMDB 18276) (PDB 8Q9C)	CTE-MIA15 (240’ R3) (EMDB 18277) (PDB 8Q9D)
**Data acquisition**				
Electron gun	FEG	FEG	FEG	FEG
Detector	Falcon 4	Falcon 4	Falcon 4	Falcon 4
Energy filter slit (eV)	na	na	na	na
Magnification	105,000	105,000	105,000	105,000
Voltage (kV)	300	300	300	300
Electron dose (e-/Å^2)	30	30	30	30
Defocus range (μM)	1.0 to 2.5	1.0 to 2.5	1.0 to 2.5	1.0 to 2.5
Pixel size (Å)	0.824	0.824	0.824	0.824
**Data processing**				
Initial particle images (no.)	208952	208952	208952	208952
Final particle images (no.)	23040	15874	14186	10660
Helical twist (°)	-1.05	179.5	-1.24	-1.25
Helical rise (Å)	4.76	2.38	4.74	4.76
Symmetry imposed	C1	C1	C1	C1
Map resolution FSC 0.143 (Å)	3.16	3.10	3.40	3.16
**Refinement**				
Initial model used (PDB code)	de novo	de novo	de novo	de novo
Model resolution FSC 0.5 (Å)	3.3	3.1	3.8	3.2
Map sharpening *B* factor (Å^2)	-53.84	-48.26	-33.30	-45.23
Model composition				
Non-hydrogen atoms	3444	3444	3444	3444
Protein residues	450	450	450	450
Ligands	0	0	0	0
B factors (Å^2)				
Protein	94.85	94.85	94.85	94.85
Ligand	na	na	na	na
R.m.s. deviations				
Bond lengths (Å)	0.010	0.010	0.010	0.010
Bond angles (°)	1.982	1.891	2.056	1.900
Validation				
MolProbity score	0.82	0.78	0.90	1.11
Clashscore	0.00	0.00	0.00	0.85
Poor rotamers (%)	0.00	0.00	0.00	0.00
Ramachandran plot				
Favored (%)	95.43	95.89	94.06	94.98
Allowed (%)	4.57	4.11	5.94	5.02
Disallowed (%)	0	0	0	0

Supplementary Table 18: Refinement statistics 17. Statistics are shown only for the atomic models that were refined in the highest-resolution map.

**LMB Krios G2**	**CTE-MIA16**<br>(240' R3)<br>(EMDB 18355)
**Data acquisition**	
Electron gun	FEG
Detector	Falcon 4
Energy filter slit (eV)	na
Magnification	105,000
Voltage (kV)	300
Electron dose (e-/Å^2)	30
Defocus range (μM)	1.0 to 2.5
Pixel size (Å)	0.824
**Data processing**	
Initial particle images (no.)	208952
Final particle images (no.)	9660
Helical twist (°)	-1.14
Helical rise (Å)	4.75
Symmetry imposed	C1
Map resolution FSC 0.143 (Å)	4.59
**Refinement**	
Initial model used (PDB code)	
Model resolution FSC 0.5 (Å)	
Map sharpening *B* factor (Å^2)	
Model composition	
Non-hydrogen atoms	
Protein residues	
Ligands	
B factors (Å^2)	
Protein	
Ligand	
R.m.s. deviations	
Bond lengths (Å)	
Bond angles (°)	
Validation	
MolProbity score	
Clashscore	
Poor rotamers (%)	
Ramachandran plot	
Favored (%)	
Allowed (%)	
Disallowed (%)	

Supplementary Table 19: Refinement statistics 18. Statistics are shown only for the atomic models that were refined in the highest-resolution map.

LMB Krios G2	CTE-MIA9 (240’ R2) (EMDB 18270) (PDB 8Q97)	CTE-MIA10 (240’ R2) (EMDB 18272) (PDB 8Q99)	CTE-LIA1 (240’ R2) (EMDB 18358)	CTE-LIA2 (240’ R2) (EMDB 18359)
**Data acquisition**				
Electron gun	FEG	FEG	FEG	FEG
Detector	Falcon 4	Falcon 4	Falcon 4	Falcon 4
Energy filter slit (eV)	na	na	na	na
Magnification	105,000	105,000	105,000	105,000
Voltage (kV)	300	300	300	300
Electron dose (e-/Å^2)	30	30	30	30
Defocus range (μM)	1.0 to 2.5	1.0 to 2.5	1.0 to 2.5	1.0 to 2.5
Pixel size (Å)	0.824	0.824	0.824	0.824
**Data processing**				
Initial particle images (no.)	123830	123830	123830	123830
Final particle images (no.)	18265	38067	7709	4486
Helical twist (º)	-1.14	-1.30	-1.32	-1.22
Helical rise (Å)	4.77	4.77	4.75	4.75
Symmetry imposed	C2	C1	C1	C1
Map resolution FSC 0.143 (Å)	2.99	2.70	8.56	5.97
**Refinement**				
Initial model used (PDB code)	de novo	de novo		
Model resolution FSC 0.5 (Å)	3.1	2.5		
Map sharpening *B* factor (Å^2)	-44.91	-42.43		
Model composition				
Non-hydrogen atoms	3246	3246		
Protein residues	426	426		
Ligands	0	0		
B factors (Å^2)				
Protein	39.48	39.48		
Ligand	na	na		
R.m.s. deviations				
Bond lengths (Å)	0.011	0.010		
Bond angles (°)	2.084	1.911		
Validation				
MolProbity score	0.50	0.63		
Clashscore	0.00	0.00		
Poor rotamers (%)	0.00	0.00		
Ramachandran plot				
Favored (%)	99.28	97.34		
Allowed (%)	0.72	2.66		
Disallowed (%)	0	0		

Supplementary Table 20: Refinement statistics 19. Statistics are shown only for the atomic models that were refined in the highest-resolution map.

LMB Krios G2	CTE-MIA11 (240' R6) (EMDB 18273) (PDB 8Q9A)
**Data acquisition**	
Electron gun	FEG
Detector	Falcon 4
Energy filter slit (eV)	na
Magnification	105,000
Voltage (kV)	300
Electron dose (e-/Å^2)	30
Defocus range (μM)	1.0 to 2.5
Pixel size (Å)	0.824
**Data processing**	
Initial particle images (no.)	234520
Final particle images (no.)	44009
Helical twist (°)	-1.24
Helical rise (Å)	4.76
Symmetry imposed	C1
Map resolution FSC 0.143 (Å)	3.04
**Refinement**	
Initial model used (PDB code)	de novo
Model resolution FSC 0.5 (Å)	3.2
Map sharpening *B* factor (Å^2)	-71.45
Model composition	
Non-hydrogen atoms	3507
Protein residues	462
Ligands	0
B factors (Å^2)	
Protein	96.76
Ligand	na
R.m.s. deviations	
Bond lengths (Å)	0.010
Bond angles (°)	1.876
Validation	
MolProbity score	0.81
Clashscore	0.42
Poor rotamers (%)	0.00
Ramachandran plot	
Favored (%)	97.11
Allowed (%)	2.89
Disallowed (%)	0

Supplementary Table 21: Refinement statistics 20. Statistics are shown only for the atomic models that were refined in the highest-resolution map.

TFS Krios G4	CTE-LIA3 (300' R1) (EMDB 18279) (PDB 8Q9F)	CTE-LIA4 (300' R1) (EMDB 18281) (PDB 8Q9H)	CTE-LIA5 (300' R1) (EMDB 18280) (PDB 8Q9G)	CTE-LIA6 (300' R1) (EMDB 18282) (PDB 8Q9I)
**Data acquisition**				
Electron gun	CFEG	CFEG	CFEG	CFEG
Detector	Falcon 4i	Falcon 4i	Falcon 4i	Falcon 4i
Energy filter slit (eV)	10	10	10	10
Magnification	165,000	165,000	165,000	165,000
Voltage (kV)	300	300	300	300
Electron dose (e-/$Å^2$)	40	40	40	40
Defocus range (μM)	1.0 to 2.5	1.0 to 2.5	1.0 to 2.5	1.0 to 2.5
Pixel size (Å)	0.727	0.727	0.727	0.727
**Data processing**				
Initial particle images (no.)	361062	361062	361062	361062
Final particle images (no.)	126250	48346	24805	18914
Helical twist (°)	-1.45	179.389	-1.2	-1.48
Helical rise (Å)	4.74	2.374	4.76	4.76
Symmetry imposed	C1	C1	C1	C1
Map resolution FSC 0.143 (Å)	1.93	2.18	2.65	2.56
**Refinement**				
Initial model used (PDB code)	de novo	de novo	de novo	de novo
Model resolution FSC 0.5 (Å)	2.0	2.1	3.0	2.5
Map sharpening *B* factor ($Å^2$)	-30.35	-27.78	-34.15	-27.70
Model composition				
Non-hydrogen atoms	3132	3342	3342	1671
Protein residues	414	438	438	219
Ligands	0	0	0	0
B factors ($Å^2$)				
Protein	101	110	110	110
Ligand	na	na	na	na
R.m.s. deviations				
Bond lengths (Å)	0.011	0.010	0.011	0.010
Bond angles (°)	1.872	1.835	2.049	1.860
Validation				
MolProbity score	0.62	0.65	0.50	0.75
Clashscore	0.31	0.00	0.00	0.00
Poor rotamers (%)	0.00	0.00	0.00	0.00
Ramachandran plot				
Favored (%)	99.75	97.18	98.36	96.24
Allowed (%)	0.25	2.82	1.64	3.76
Disallowed (%)	0	0	0	0

Supplementary Table 22: Refinement statistics 21. Statistics are shown only for the atomic models that were refined in the highest-resolution map.

TFS Krios G4	CTE-LIA7 (300' R1) (EMDB 18283) (PDB 8Q9J)	CTE-LIA14 (300' R1) (EMDB 18285) (PDB 8Q9L)
**Data acquisition**		
Electron gun	CFEG	CFEG
Detector	Falcon 4i	Falcon 4i
Energy filter slit (eV)	10	10
Magnification	165,000	165,000
Voltage (kV)	300	300
Electron dose (e-/Å^2)	40	40
Defocus range (μM)	1.0 to 2.5	1.0 to 2.5
Pixel size (Å)	0.727	0.727
**Data processing**		
Initial particle images (no.)	361062	361062
Final particle images (no.)	5127	16784
Helical twist (º)	-1.32	119.496
Helical rise (Å)	4.74	1.58
Symmetry imposed	C1	C1
Map resolution FSC 0.143 (Å)	2.96	2.76
**Refinement**		
Initial model used (PDB code)	de novo	de novo
Model resolution FSC 0.5 (Å)	2.9	2.4
Map sharpening *B* factor (Å^2)	-30.73	-43.56
Model composition		
Non-hydrogen atoms	3342	5013
Protein residues	438	657
Ligands	0	0
B factors (Å^2)		
Protein	110.13	110.93
Ligand	na	na
R.m.s. deviations		
Bond lengths (Å)	0.011	0.010
Bond angles (°)	1.928	1.864
Validation		
MolProbity score	0.50	0.50
Clashscore	0.00	0.00
Poor rotamers (%)	0.00	0.00
Ramachandran plot		
Favored (%)	98.83	98.44
Allowed (%)	1.17	1.56
Disallowed (%)	0	0

Supplementary Table 23: Refinement statistics 22. Statistics are shown only for the atomic models that were refined in the highest-resolution map.

LMB Krios G2	**CTE-MIA19** (300' R3) (EMDB 18356)	**CTE-LIA8** (300' R3) (EMDB 18360)	**CTE-LIA9** (300' R3) (EMDB 18361)	**CTE-LIA10** (300' R3) (EMDB 18362)
**Data acquisition**				
Electron gun	FEG	FEG	FEG	FEG
Detector	Falcon 4	Falcon 4	Falcon 4	Falcon 4
Energy filter slit (eV)	na	na	na	na
Magnification	105,000	105,000	105,000	105,000
Voltage (kV)	300	300	300	300
Electron dose (e-/Å^2)	30	30	30	30
Defocus range (μM)	1.0 to 2.5	1.0 to 2.5	1.0 to 2.5	1.0 to 2.5
Pixel size (Å)	0.824	0.824	0.824	0.824
**Data processing**				
Initial particle images (no.)	148997	148997	148997	148997
Final particle images (no.)	3787	20871	12179	4071
Helical twist (°)	-1.20	-1.17	-1.09	-0.80
Helical rise (Å)	4.76	4.75	4.65	4.35
Symmetry imposed	C1	C1	C1	C1
Map resolution FSC 0.143 (Å)	6.73	8.55	5.97	21.1
**Refinement**				
Initial model used (PDB code)				
Model resolution FSC 0.5 (Å)				
Map sharpening *B* factor (Å^2)				
Model composition				
Non-hydrogen atoms				
Protein residues				
Ligands				
B factors (Å^2)				
Protein				
Ligand				
R.m.s. deviations				
Bond lengths (Å)				
Bond angles (°)				
Validation				
MolProbity score				
Clashscore				
Poor rotamers (%)				
Ramachandran plot				
Favored (%)				

Supplementary Table 24: Refinement statistics 23. Statistics are shown only for the atomic models that were refined in the highest-resolution map.

LMB Krios G2	CTE-LIA12 (300’ R6) (EMDB 18364)	CTE-LIA13 (300’ R6) (EMDB 18284) (PDB 8Q9K)	CTE-LIA15 (300’ R6) (EMDB 18365)
**Data acquisition**			
Electron gun	FEG	FEG	FEG
Detector	Falcon 4	Falcon 4	Falcon 4
Energy filter slit (eV)	na	na	na
Magnification	105,000	105,000	105,000
Voltage (kV)	300	300	300
Electron dose (e-/Å^2)	30	30	30
Defocus range (μM)	1.0 to 2.5	1.0 to 2.5	1.0 to 2.5
Pixel size (Å)	0.824	0.824	0.824
**Data processing**			
Initial particle images (no.)	181708	181708	181708
Final particle images (no.)	20658	15464	6252
Helical twist (º)	-1.2	179.353	-1.25
Helical rise (Å)	4.79	2.39	4.84
Symmetry imposed	C1	C1	C1
Map resolution FSC 0.143 (Å)	3.86	3.20	5.27
**Refinement**			
Initial model used (PDB code)		de novo	
Model resolution FSC 0.5 (Å)		3.0	
Map sharpening *B* factor (Å^2)		-37.66	
Model composition			
Non-hydrogen atoms		3090	
Protein residues		408	
Ligands		0	
B factors (Å^2)			
Protein		96.76	
Ligand		na	
R.m.s. deviations			
Bond lengths (Å)		0.010	
Bond angles (°)		2.063	
Validation			
MolProbity score		1.28	
Clashscore		1.75	
Poor rotamers (%)		0.00	
Ramachandran plot			
Favored (%)		94.95	
Allowed (%)		5.05	
Disallowed (%)		0	

Supplementary Table 25: Refinement statistics 24. Statistics are shown only for the atomic models that were refined in the highest-resolution map.

LMB Krios G2	CTE-LIA16<br>(360' R1)<br>(EMDB 18366)
**Data acquisition**	
Electron gun	FEG
Detector	Falcon 4
Energy filter slit (eV)	na
Magnification	105,000
Voltage (kV)	300
Electron dose (e-/Å^2)	30
Defocus range (μM)	1.0 to 2.5
Pixel size (Å)	0.824
**Data processing**	
Initial particle images (no.)	233495
Final particle images (no.)	7426
Helical twist (º)	119.54
Helical rise (Å)	1.90
Symmetry imposed	C1
Map resolution FSC 0.143 (Å)	4.33
**Refinement**	
Initial model used (PDB code)	
Model resolution FSC 0.5 (Å)	
Map sharpening *B* factor (Å^2)	
Model composition	
Non-hydrogen atoms	
Protein residues	
Ligands	
B factors (Å^2)	
Protein	
Ligand	
R.m.s. deviations	
Bond lengths (Å)	
Bond angles (°)	
Validation	
MolProbity score	
Clashscore	
Poor rotamers (%)	
Ramachandran plot	
Favored (%)	
Allowed (%)	
Disallowed (%)	

Supplementary Table 26: Refinement statistics 25. Statistics are shown only for the atomic models that were refined in the highest-resolution map.

LMB Krios G2	CTE-LIA17 (720' R5) (EMDB 18287) (PDB 8Q9O)
**Data acquisition**	
Electron gun	FEG
Detector	Falcon 4
Energy filter slit (eV)	na
Magnification	105,000
Voltage (kV)	300
Electron dose (e-/Å^2)	30
Defocus range (μM)	1.0 to 2.5
Pixel size (Å)	0.824
**Data processing**	
Initial particle images (no.)	43926
Final particle images (no.)	19988
Helical twist (°)	179.262
Helical rise (Å)	2.39
Symmetry imposed	C1
Map resolution FSC 0.143 (Å)	3.1
**Refinement**	
Initial model used (PDB code)	de novo
Model resolution FSC 0.5 (Å)	2.9
Map sharpening *B* factor (Å^2)	-49.83
Model composition	
Non-hydrogen atoms	3444
Protein residues	450
Ligands	0
B factors (Å^2)	
Protein	115
Ligand	na
R.m.s. deviations	
Bond lengths (Å)	0.011
Bond angles (°)	1.941
Validation	
MolProbity score	1.36
Clashscore	1.14
Poor rotamers (%)	3.03
Ramachandran plot	
Favored (%)	97.03
Allowed (%)	2.97
Disallowed (%)	0

Supplementary Table 27: Refinement statistics 26. Statistics are shown only for the atomic models that were refined in the highest-resolution map.

LMB Krios G2	CTE-MIA20 (720' R1) (EMDB 18357)	CTE-type I (720' R1) (EMDB 18286) (PDB 8Q9M)
**Data acquisition**		
Electron gun	FEG	FEG
Detector	Falcon 4	Falcon 4
Energy filter slit (eV)	na	na
Magnification	105,000	105,000
Voltage (kV)	300	300
Electron dose (e-/Å^2)	30	30
Defocus range (μM)	1.0 to 2.5	1.0 to 2.5
Pixel size (Å)	0.824	0.824
**Data processing**		
Initial particle images (no.)	36037	36037
Final particle images (no.)	3205	25924
Helical twist (°)	179.49	179.43
Helical rise (Å)	2.45	2.376
Symmetry imposed	C1	C1
Map resolution FSC 0.143 (Å)	5.02	2.65
**Refinement**		
Initial model used (PDB code)		de novo
Model resolution FSC 0.5 (Å)		2.5
Map sharpening *B* factor (Å^2)		-43.39
Model composition		
Non-hydrogen atoms		3444
Protein residues		450
Ligands		0
B factors (Å^2)		
Protein		115
Ligand		na
R.m.s. deviations		
Bond lengths (Å)		0.01
Bond angles (°)		1.859
Validation		
MolProbity score		0.82
Clashscore		1.14
Poor rotamers (%)		0.00
Ramachandran plot		
Favored (%)		98.17
Allowed (%)		1.83
Disallowed (%)		0

Supplementary Table 28: Refinement statistics 27. Statistics are shown only for the atomic models that were refined in the highest-resolution map.

LMB Krios G2	CTE-type II (720’ R3) (EMDB 18448) (PDB 8QJJ)
**Data acquisition**	
Electron gun	FEG
Detector	Falcon 4
Energy filter slit (eV)	na
Magnification	105,000
Voltage (kV)	300
Electron dose (e-/Å^2)	30
Defocus range (μM)	1.0 to 2.5
Pixel size (Å)	0.824
**Data processing**	
Initial particle images (no.)	32580
Final particle images (no.)	2280
Helical twist (°)	-1.19
Helical rise (Å)	4.78
Symmetry imposed	C1
Map resolution FSC 0.143 (Å)	3.35
**Refinement**	
Initial model used (PDB code)	6nwq
Model resolution FSC 0.5 (Å)	3.2
Map sharpening *B* factor (Å^2)	-45.1
Model composition	
Non-hydrogen atoms	3444
Protein residues	450
Ligands	0
B factors (Å^2)	
Protein	115
Ligand	na
R.m.s. deviations	
Bond lengths (Å)	0.011
Bond angles (°)	1.993
Validation	
MolProbity score	1.28
Clashscore	0.91
Poor rotamers (%)	0
Ramachandran plot	
Favored (%)	97.72
Allowed (%)	2.28
Disallowed (%)	0

Supplementary Table 29: Refinement statistics 28. Statistics are shown only for the atomic models that were refined in the highest-resolution map.

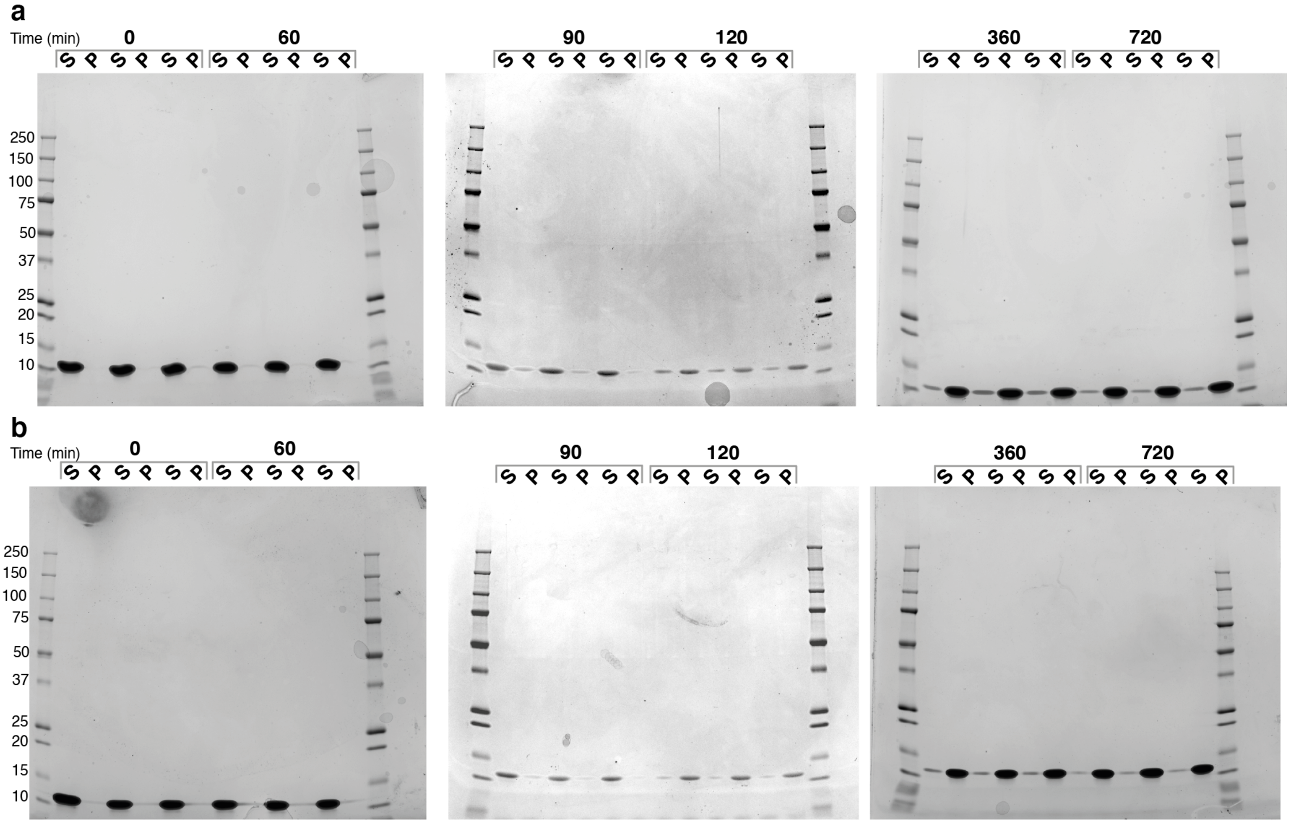

Supplementary Figure 53: SDS-PAGE gels for the quantification of pelletable tau. Coomassie stained sodium dodecyl-sulfate polyacrylamide gel electrophoresis (SDS-PAGE, 4-20% Tris-glycine) of pelletable material in the assembly reactions. 1.5 microliter of supernatant or pellet was loaded onto each well, **a.** for the AD reaction and **b.** for the CTE reaction. S: supernatant, P: pellet. Time is denoted in minutes.
