# Supplementary material for: Disease-specific tau filaments assemble via polymorphic intermediates
Source: Nature. 2023 Nov 29;625(7993):119–25. doi: 10.1038/s41586-023-06788-w (PMC10764278; doi:10.1038/s41586-023-06788-w)
Supplement: Supplementary file 2 — Reporting Summary [file 41586_2023_6788_MOESM2_ESM.pdf]

nature portfolio

Corresponding author(s): Sjors Scheres & Michel Goedert

Last updated by author(s): Oct 13, 2023

# Reporting Summary

Nature Portfolio wishes to improve the reproducibility of the work that we publish. This form provides structure for consistency and transparency in reporting. For further information on Nature Portfolio policies, see our Editorial Policies and the Editorial Policy Checklist.

## Statistics

For all statistical analyses, confirm that the following items are present in the figure legend, table legend, main text, or Methods section.

| n/a | Confirmed | |
|---|---|---|
| ☐ | ☒ | The exact sample size (*n*) for each experimental group/condition, given as a discrete number and unit of measurement |
| ☐ | ☒ | A statement on whether measurements were taken from distinct samples or whether the same sample was measured repeatedly |
| ☒ | ☐ | The statistical test(s) used AND whether they are one- or two-sided<br>*Only common tests should be described solely by name; describe more complex techniques in the Methods section.* |
| ☒ | ☐ | A description of all covariates tested |
| ☒ | ☐ | A description of any assumptions or corrections, such as tests of normality and adjustment for multiple comparisons |
| ☒ | ☐ | A full description of the statistical parameters including central tendency (e.g. means) or other basic estimates (e.g. regression coefficient) AND variation (e.g. standard deviation) or associated estimates of uncertainty (e.g. confidence intervals) |
| ☒ | ☐ | For null hypothesis testing, the test statistic (e.g. *F*, *t*, *r*) with confidence intervals, effect sizes, degrees of freedom and *P* value noted<br>*Give P values as exact values whenever suitable.* |
| ☒ | ☐ | For Bayesian analysis, information on the choice of priors and Markov chain Monte Carlo settings |
| ☒ | ☐ | For hierarchical and complex designs, identification of the appropriate level for tests and full reporting of outcomes |
| ☒ | ☐ | Estimates of effect sizes (e.g. Cohen's *d*, Pearson's *r*), indicating how they were calculated |

*Our web collection on statistics for biologists contains articles on many of the points above.*

## Software and code

Policy information about availability of computer code

| | |
|---|---|
| Data collection | For cryo-EM: EPU (v2.3.079) (Thermofisher). For NMR: Topspin (v3.2 and 4) (Bruker), |
| Data analysis | RELION (v4 and v5), CTFFIND (v4.1), COOT (v0.9.8.7), ChimeraX (v1.6.1), ImageJ (v2.1.0/1.53c), Prism (v9), GUSSI (v1.4.2), Sednterp (v3.0.4), SEDFIT-16.1c, Topspin (v3.2, 3.6.3 and 4.0) ), NMRpipe (v10.9), qMDD (v3.1), NMRFAM-Sparky (v1.470) , MARS (v1.2), Mathematics (v13.1), FiTSuite (v0.1). In addition, we developed and used cryoEM image processing scripts in python called FilamentTools, which can be downloaded from https://github.com/dbli2000/FilamentTools. It has no version number. |

For manuscripts utilizing custom algorithms or software that are central to the research but not yet described in published literature, software must be made available to editors and reviewers. We strongly encourage code deposition in a community repository (e.g. GitHub). See the Nature Portfolio guidelines for submitting code & software for further information.

## Data

Policy information about availability of data

All manuscripts must include a data availability statement. This statement should provide the following information, where applicable:

- Accession codes, unique identifiers, or web links for publicly available datasets
- A description of any restrictions on data availability
- For clinical datasets or third party data, please ensure that the statement adheres to our policy

All structures described have been submitted to the EMDB and PDB. Accession codes have been listed.

## Research involving human participants, their data, or biological material

Policy information about studies with human participants or human data. See also policy information about sex, gender (identity/presentation), and sexual orientation and race, ethnicity and racism.

| | |
|---|---|
| Reporting on sex and gender | NA |
| Reporting on race, ethnicity, or other socially relevant groupings | NA |
| Population characteristics | NA |
| Recruitment | NA |
| Ethics oversight | NA |

Note that full information on the approval of the study protocol must also be provided in the manuscript.

# Field-specific reporting

Please select the one below that is the best fit for your research. If you are not sure, read the appropriate sections before making your selection.

☒ Life sciences ☐ Behavioural & social sciences ☐ Ecological, evolutionary & environmental sciences

For a reference copy of the document with all sections, see nature.com/documents/nr-reporting-summary-flat.pdf

# Life sciences study design

All studies must disclose on these points even when the disclosure is negative.

| | |
|---|---|
| Sample size | Six samples were made for the CTE reaction and five for AD reactions which were analysed by cryo-EM. Aliquots were taken at different time intervals to sample the assembly reaction (which is shown in the supplementary information and in Figure 4 in the main text). These sample sizes were chosen such that at least 3 samples were available for cryo-EM at each time point (as not enough volume is present in each sample to take aliquots for all time points and not all cryo-EM grids have suitable ice for imaging).<br>Three samples from the CTE reaction and three samples from the AD reactions were used for continuous ThT monitoring to determine the kinetics of amyloid assembly in the reaction.<br>Three samples of each condition were used to determine the amount of pelletable tau which was analysed by SDS-PAGE and shown in the supplementary information.<br>These samples were also used for the off-line monitoring of ThT fluorescence. This was to test whether ThT molecule affected the kinetics of the assembly reaction which is discussed in the main text of the paper. |
| Data exclusions | No data has been excluded. An overview of all data is provided in the Supplementary Information file. |
| Replication | Experiments were performed 5 and 6 times to establish the pathway of intermediate folding, collecting data points at different time points. Not all cryo-EM grids contained suitable ice for imaging and there was not enough volume in them to sample all time points, requiring five or six replicate reactions to achieve at least 3 cryo-EM data sets for all time points for both reactions. For continuous kinetic studies (ThT measuring), experiments were performed three times. For pelletable material, experiments were performed three times. |
| Randomization | Because there is no assignment of data points to distinct groups, randomization was not applicable to this study. Perhaps the only relevant randomization is that of determining random half-sets of particles for resolution assessment in cryo-EM reconstructions. This randomization was done using random number generators inside the RELION program. |
| Blinding | No blinding was performed, as the risk for bias by the experimentalist was deemed irrelevant for this study. |

# Reporting for specific materials, systems and methods

We require information from authors about some types of materials, experimental systems and methods used in many studies. Here, indicate whether each material, system or method listed is relevant to your study. If you are not sure if a list item applies to your research, read the appropriate section before selecting a response.

## Materials & experimental systems

| n/a | Involved in the study |
| --- | --- |
| ☒ | ☐ Antibodies |
| ☒ | ☐ Eukaryotic cell lines |
| ☒ | ☐ Palaeontology and archaeology |
| ☒ | ☐ Animals and other organisms |
| ☒ | ☐ Clinical data |
| ☒ | ☐ Dual use research of concern |
| ☒ | ☐ Plants |

## Methods

| n/a | Involved in the study |
| --- | --- |
| ☒ | ☐ ChIP-seq |
| ☒ | ☐ Flow cytometry |
| ☒ | ☐ MRI-based neuroimaging |
